# Metal–Organic Frameworks: Multifunctional Materials for High-Performance Zn-Halogen Batteries

**DOI:** 10.1007/s40820-026-02068-0

**Published:** 2026-02-06

**Authors:** Ayesha Arif, Xinrui Yan, Adil Mansoor, Tazeen Fatima, Tayyaba Najam, Hassan Akhtar, Muhammad Sufyan Javed, Manzar Sohail, Muhammad Altaf Nazir, Jiantao Zai, Xiaowei Yang, Syed Shoaib Ahmad Shah

**Affiliations:** 1 Catalysis and Surface Chemistry Laboratory, Department of Chemistry, School of Natural Sciences, National University of Sciences and Technology (NUST), Islamabad, 44000 Pakistan; 2 Shanghai Electrochemical Energy Devices Research Center, School of Chemistry and Chemical Engineering, Shanghai Jiao Tong University, Shanghai, 200240 People’s Republic of China; 3 Shenzhen Key Laboratory of Advanced Thin Films and Applications, College of Physics and Optoelectronic Engineering, Shenzhen University, Shenzhen, Guangdong 518060 People’s Republic of China; 4 Institute of Chemistry, The Islamia University of Bahawalpur, Bahawalpur, 63100 Pakistan; 5 National Synchrotron Radiation Laboratory, Key Laboratory of Precision and Intelligent Chemistry, School of Nuclear Science and Technology, University of Science and Technology of China, Hefei, Anhui 230029 People’s Republic of China; 6 Institute of Carbon Neutrality, Zhejiang Wanli University, Ningbo, 315100 People’s Republic of China

**Keywords:** Metal–organic frameworks (MOFs), Zinc-halogen batteries, Shuttle effect, Polyhalide confinement, Energy storage

## Abstract

Aqueous zinc batteries are gaining attention as promising alternatives to Li-ion systems, owing to the increased need for safe and cost-effective energy storage. Aqueous Zn-halogen batteries are particularly important because of their low cost and the abundance of precursors. However, critical challenges, such as the shuttle effect, sluggish redox kinetics, and dendrite growth, impede their practical development. Metal–organic frameworks (MOFs) with high porosity, ease of functionalization, and stability offer a multifunctional approach to overcome these limitations. This review systematically examines the advancements in MOF-based Zn-halogen batteries, focusing on their roles in different components of the battery, including the cathode, anode, and separator. This review also highlights the key design strategies for MOF-based materials and then examines the structure–performance relationships through advanced characterization and computational insights. The remaining challenges and future directions are also outlined. Overall, this review provides a roadmap for developing advanced MOF-based Zn-halogen batteries that combine high energy density and long-term durability for next-generation energy storage applications.

## Highlights

- This review comprehensively summarizes the application of metal-organic frameworks (MOFs) in aqueous Zn-halogen batteries, covering their roles as cathodes, anodes, and separators.
- The mechanism of MOFs in suppressing the shuttle effect via nanoconfinement, inhibiting dendrite growth by regulating ion flux, and enhancing redox kinetics through catalytic sites are thoroughly discussed.
- The structure-performance relationships of MOFs in Zn-halogen batteries are elucidated, linking their porosity, metal nodes, and linker functionalities to overall battery performance.

## Graphical abstract

**Figure:**
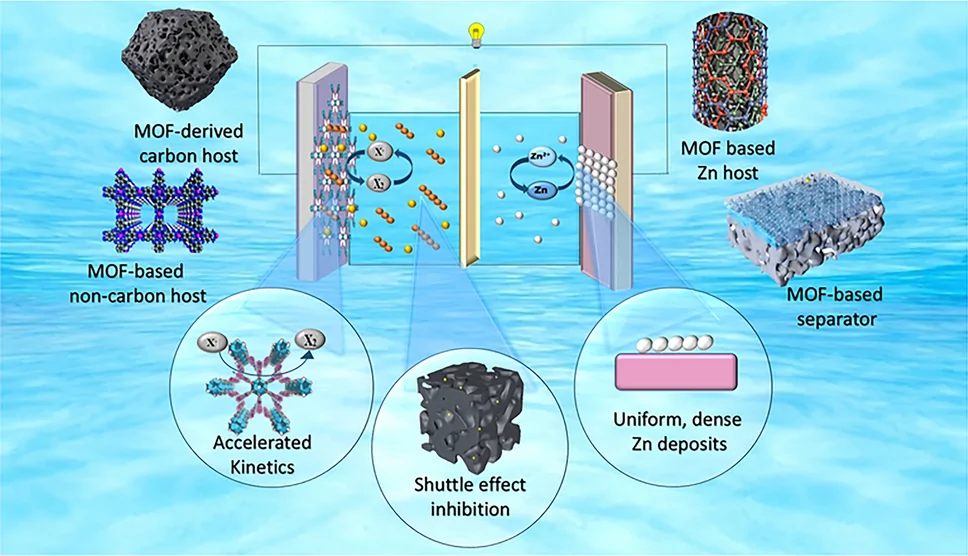

## Introduction

The global shift toward renewable energy sources, along with the growing demand for efficient energy storage systems, has accelerated research in advanced battery technologies [1]. Lithium-ion batteries (LIBs) have achieved tremendous commercial success because of their high energy density, long cycle life, and high rate capability [2,3]. However, the limited availability of lithium resources and safety risks associated with flammable organic electrolytes have urged scientists to explore cost-effective and safer alternatives for large-scale energy storage [4]. Aqueous metal batteries have attracted considerable attention in recent years, primarily because of the intrinsic safety of water-based electrolytes [5,6]. Among them, rechargeable aqueous zinc-based batteries (ARZBs) are considered promising alternatives for grid-scale energy storage because of their low cost, abundant resources, and intrinsic safety [7–10]. Zn-based batteries have several advantages. Zn can be directly employed as an anode in aqueous batteries, offering a high theoretical capacity of 820 mAh g^−1^, excellent volumetric capacity of 5855 mAh cm^−3^, and low voltage of − 0.76 V vs a standard hydrogen electrode (SHE) [11,12]. In addition, Zn-based batteries can be assembled in an air atmosphere because Zn metal is insensitive to oxygen and humid atmospheres, reducing the additional costs arising from fabrication under inert conditions [13].

Various ARZBs have been developed in the past few years. Based on cathodic material and reaction mechanisms, there are different types of ARZBs [14]. Intercalation types, including Zn-MnO_2_ [15,16], Zn-V_2_O_5_ [17], Zn-MoO_3_ [18], and Prussian blue analogs [19], involve reversible Zn^2+^ intercalation and extraction. Alkaline Zn batteries such as Zn-Ag_2_O [20], Zn-Ni(OH)_2_ [21], and Zn-air [22–24] are based on the conversion reaction of cathodes. Zn-based redox flow batteries utilize redox active species in catholytes, including Zn-Fe [25] and Zn-halogens [26]. Among these, aqueous Zn-halogen batteries (AZHBs) such as Zn-Cl_2_ [27], Zn-Br_2_ [28], and Zn-I_2_ [29] have gained significant attention because of their high energy density, low cost, and excellent electrochemical performance. Aqueous zinc iodine batteries utilizing the iodine redox reaction I^−^/I^0^ can deliver a theoretical capacity of 211 mAh g^−1^ and up to 422 mAh g^−1^ when full I^−^/I^0^/I^+^ is used [30,31]. Similarly, aqueous Zn-Br_2_ can reach 335 mAh g^−1^ from Br^−^/Br^0^ redox reactions [32], whereas Zn-Cl_2_ exhibits a higher theoretical capacity of 755 mAh g^−1^ based on Cl^−^/Cl^0^ redox reactions [33]. Zn-Br_2_ batteries have shown substantial progress among Zn-halogen batteries, with the first commercialization in the 1970s by Exxon [34]. Despite advancements, Zn-Br_2_ still encounters challenges, particularly related to the volatility and shuttling of polybromide species across the separator, which can corrode the Zn anode and cause sluggish kinetics of the Br_2_ redox couple [35]. In comparison, Zn-I_2_ batteries (ZIBs) represent a more stable and safer system owing to iodine’s high boiling point and low volatility [36,37]. The research on Zn-Cl_2_ batteries (ZCBs) is limited. Despite the high theoretical capacity of 755 mAh g^−1^, the toxic and volatile nature of chlorine gas raises major concerns regarding safety and operation [38].

Based on their configuration, Zn-halogen batteries are categorized into flow [39] and static systems [40]. Static batteries store energy in solid or fixed electrodes, whereas flow batteries store energy in liquid electrolytes that are pumped into the electrochemical cells, allowing scalable capacity by simply increasing the electrolyte volume [41]. Halogen batteries face similar challenges in both static and flow configurations, such as halogen crossover, sluggish redox kinetics, dendrite growth over the Zn anode, hydrogen evolution reaction at the anode attributed to the limited electrolyte stability potential window, and corrosion at the anode [42,43]. Overcoming these problems requires the development of novel materials, efficient structural designs, and improved engineering methodologies to enable the practical implementation of aqueous zinc-halogen batteries.

Metal–organic frameworks (MOFs) have emerged as versatile and multifunctional materials in the design of Zn-halogen batteries, offering distinct advantages over other porous materials like traditional carbons or metal oxides. While materials such as activated carbon provide high surface area, their amorphous nature and poorly defined pore environments offer limited control over host–guest interactions [44]. In contrast, MOFs provide large surface area, tunable porosity, diverse functionalities, and atomically dispersed catalytic centers [45–47]. This allows for the creation of a tailored environment that not only physically confines halogen species but also chemically binds them via specific functional sites. These properties make MOFs ideal materials for halogen confinement, improving redox kinetics, enabling uniform Zn deposition, and facilitating selective ion diffusion [42,48,49]. Addressing various challenges, MOFs can act as versatile candidates for various roles in Zn-halogen batteries, ranging from cathode materials [50,51] and separators [52] to anode protectors [53].

This review provides a comprehensive analysis of the design strategies, mechanistic insights, and electrochemical performance of MOF-based materials for Zn-halogen batteries. It highlights advancements in the role of MOFs in halogen confinement chemistry, redox catalysis, Zn deposition, and multifunctional separators, while addressing unresolved challenges and future opportunities. By correlating the MOF structure with the battery performance metrics, we aim to guide the development of next-generation advanced aqueous Zn-halogen batteries.

## Fundamentals of Zn-Halogen Batteries

Aqueous Zn-halogen consists of four main components: the anode, cathode, electrolyte, and separator. Both electrodes undergo electrochemical reactions during the battery working process. During charging, zinc ions in the electrolyte are reduced and deposited on the zinc anode in the form of metallic Zn, whereas the reverse reaction occurs during discharge [54].

1 $${\mathrm{Anode}}:\;\;\;{\mathrm{Zn}}^{{2 + }} + {\mathrm{2e}}^{ - } \leftrightarrow {\mathrm{Zn}}_{{({\mathrm{s}})}} \;\;{\mathrm{E}}^{{\mathrm{o}}} = - 0.{\mathrm{76}}\,{\mathrm{V}}\;{\mathrm{vs}}.\;{\mathrm{SHE}}$$

Halogens have a higher working potential in Zn-halogen batteries and act as cathodes. The electrochemical mechanism involves a reversible redox process between a neutral halogen (Xº) and its halide ion (X^−^) through electron transfer, where X typically represents Cl, Br, and I [55].

2 $${\mathrm{nX}}^{{\mathrm{o}}} + {\mathrm{ne}}^{ - } \leftrightarrow {\mathrm{nX}}^{ - }$$

**Fig. 1:**
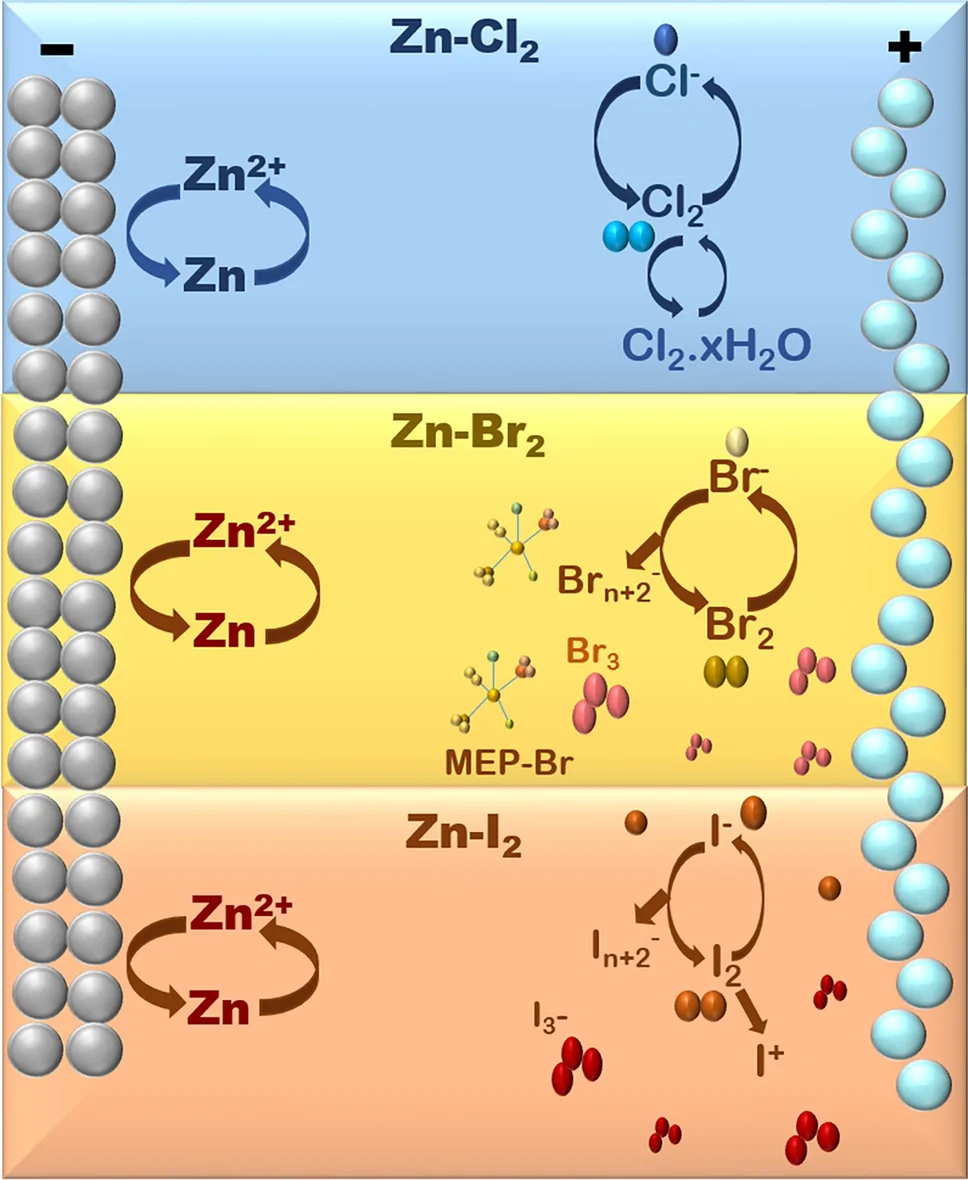
Schematic of the basic chemistry of Zn-halogen batteries

The basic chemistry of different Zn-halogen batteries is shown in Fig. 1, while Table 1 provides a comparative overview of the electrochemical properties, advantages, and key challenges of Zn-halogen battery systems.

**Table 1:** Comparative overview of the fundamental electrochemical properties for different aqueous zinc-halogen battery systems

Sr. No	Property	Zn-I_2_	Zn-Br_2_	Zn-Cl_2_
1	Cathode reaction	2I^−^_(aq)_ ↔ I_2(s)_ + 2e^−^	2Br^−^ ↔ Br_2_ + 2e^−^	Cl_2(g)_ + 2e^−^ ↔ 2Cl^−^_(aq)_
2	Reaction phase	Liquid–solid	Liquid–liquid	Gas–liquid–solid
3	Standard electrode potential (vs SHE)	0.54 V	1.07 V	1.36 V
4	Full cell potential (vs SHE)	1.29 V	1.83 V	2.12 V
5	Theoretical capacity	211 mAh g^−1^ (for 2e^−^ transfer)	335 mAh g^−1^	755 mAh g^−1^
6	Advantages	Low cost, environmental friendly	Abundant, moderate voltage	High energy density
7	Challenges	Low I_2_ conductivity, polyiodide shuttling, sluggish redox kinetics	Br_2_ crossover and corrosion, volatility, shuttle effect	Cl_2_ gas toxicity and volatility, weak adsorption, shuttle effect
8	References	[69]	[50]	[27]

### Zinc-Iodine Battery

Zinc-iodine batteries are also promising systems for grid-scale energy storage owing to their high safety, low cost, and environmental friendliness [54,56]. It uses iodine as a redox active cathode material. During charging, iodide ions (I^−^) are oxidized to form iodine (I_2_), which readily reacts with excess iodide to produce polyiodides such as I_3_^−^. The reverse process occurs during discharge [57]. Zn-I_2_ batteries using electrolytes containing I_3_^−^ or I^−^ operate through the interconversion between I_3_^−^ and I^−^ [58].

3 $${\mathrm{Cathode}}:\;\;\;{\mathrm{2I}}^{ - } \leftrightarrow {\mathrm{I}}_{{2({\mathrm{s}})}} + {\mathrm{2e}}^{ - } \;\;{\mathrm{E}} = 0.{\mathrm{536V}}\;{\mathrm{vs}}.\;{\mathrm{SHE}}$$

4 $${\mathrm{I}}_{{{2}({\mathrm{s}})}} + {\mathrm{I}}^{ - } \leftrightarrow {\mathrm{I}}_{{3}}^{ - }$$

5 $${\mathrm{I}}_{3}^{ - } + {\mathrm{2e}}^{ - } \leftrightarrow {\mathrm{3I}}^{ - } \;\;{\mathrm{E}} = 0.{\mathrm{536}}\;{\mathrm{V}}\;{\mathrm{vs}}.\;{\mathrm{SHE}}$$

6 $${\mathrm{Overall}}:\;\;\;{\mathrm{Zn}} + {\mathrm{I}}_{2} \leftrightarrow {\mathrm{Zn}}^{{2 + }} + {\mathrm{2I}}^{ - } \;\;{\mathrm{E}} = 1.{\mathrm{298V}}\;{\mathrm{vs}}.\;{\mathrm{SHE}}$$

7 $${\mathrm{Zn}} + {\mathrm{I}}_{3}^{ - } \leftrightarrow {\mathrm{Zn}}^{{2 + }} + {\mathrm{3I}}^{ - } \;\;{\mathrm{E}} = 1.{\mathrm{298}}\;{\mathrm{V}}\;{\mathrm{vs}} \cdot \;{\mathrm{SHE}}$$

Electrolyte involving I_3_^−^/I^−^ redox couple

8 $${\mathrm{3I}}^{ - } \leftrightarrow {\mathrm{I}}_{3}^{ - } + {\mathrm{2e}}^{ - } \;\;{\mathrm{E}} = - 0.{\mathrm{536V}}\;{\mathrm{vs}}.\;{\mathrm{SHE}}$$

9 $${\mathrm{Overall}}:\;\;\;{\mathrm{Zn}} + {\mathrm{I}}^{ - } \leftrightarrow {\mathrm{Zn}}^{{2 + }} + {\mathrm{I}}_{3}^{ - } \;\;{\mathrm{E}} = - 1.{\mathrm{299}}\;{\mathrm{V}}\;{\mathrm{vs}}.\;{\mathrm{SHE}}$$

**Fig. 2:**
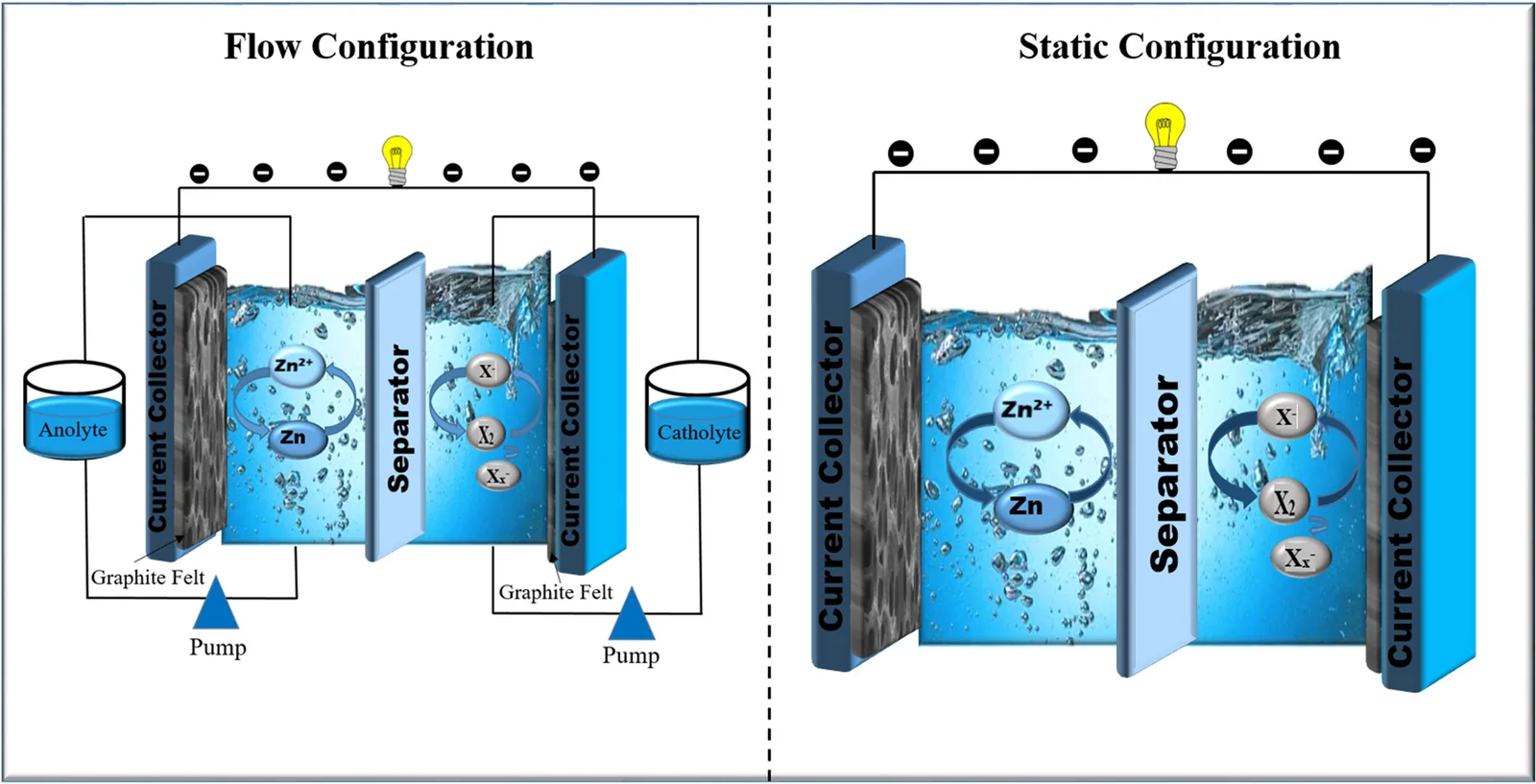
Schematic illustration of flow and static battery configurations

**Fig. 3:**
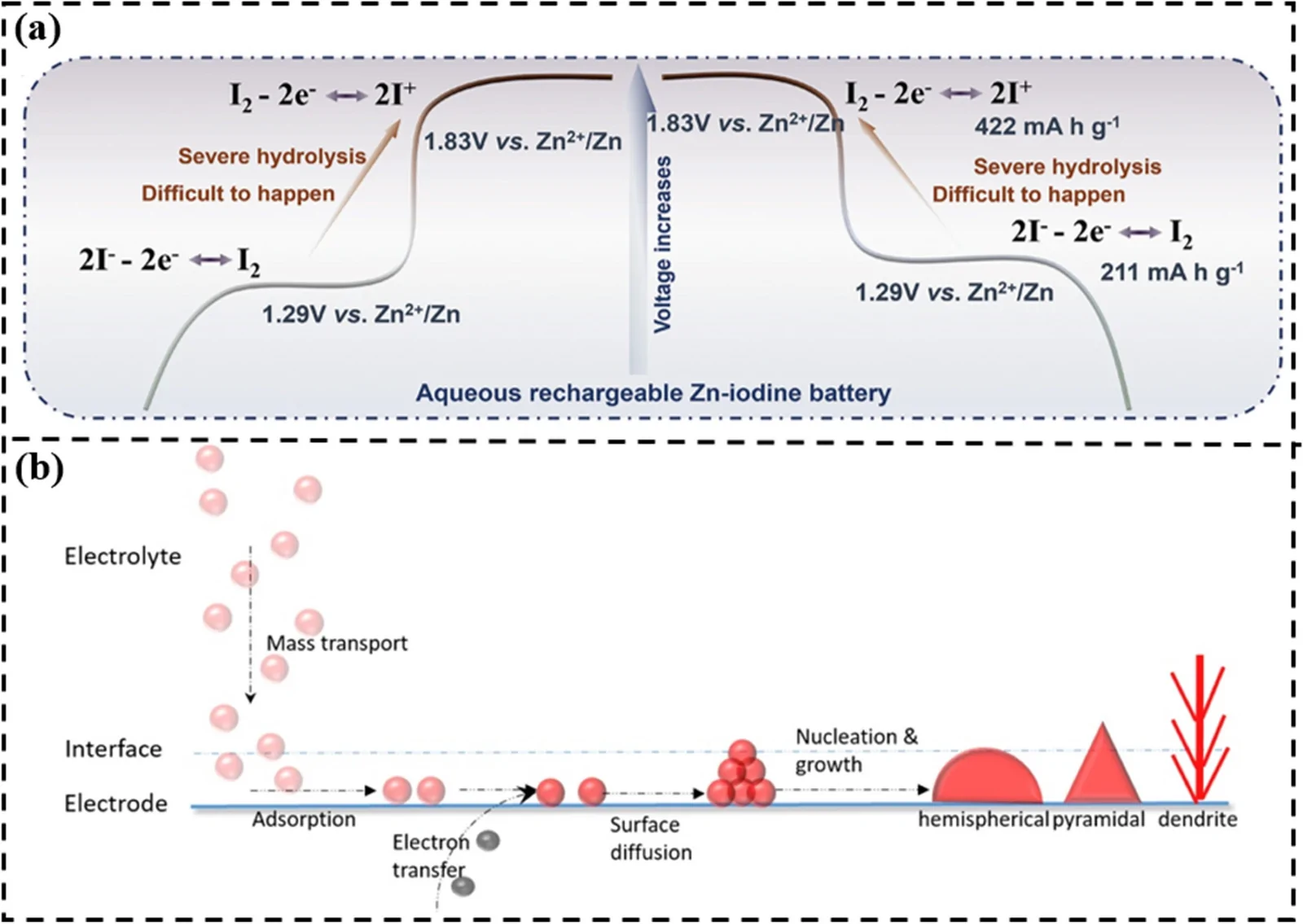
**a** Iodine redox chemistry during charging/discharging [54]. Copyright 2023, Wiley–VCH. **b** Mechanism of dendritic growth [75]. Copyright 2020, Elsevier

The conventional redox couple is a two-step redox reaction (I^−^ ↔ I_3_^−^ ↔ I_2_), which operates at 0.536 V (vs. SHE), providing a theoretical capacity of 211 mAh g^−1^ [59], while I_3_^−^/I^−^ redox couple is a one-step reaction, occurring entirely in electrolyte that also operates at 0.536 V (vs. SHE), but delivers 141 mAh g^−1^ theoretical capacity [60] (Fig. 2). Both redox couples involve a two-electron transfer electrochemical mechanism. Notably, an extended redox couple involving 2I^+^/I_2_/2I^−^ (four electron transfer process) with a high redox potential of 0.99 V vs. SHE and 1.83 V in the full cell corresponding to a theoretical capacity of 422 mAh g^−1^ has been identified, surpassing that of most conventional cathodes [31,61]. However, the facile hydrolysis of I^+^ in aqueous electrolytes limits the reversibility of this redox couple. The development of new electrolytes with low water activity and modifying electrolytes with anionic or organic additives, such as F^−^, Cl^−^, Br^−^, or CN^−^, can activate the 2I^+^/I_2_/2I^−^ redox couple [30]. In a recent study, Kong et al. used ZnI_2_ and ZnBr_2_ ionic additives in a ZnSO_4_ electrolyte to observe a four-electron transfer process, and the assembled Zn-I_2_ provided specific capacity up to 452.6 mAh g^−1^ [31]. 2e^−^ and 4e^−^ transfer iodine redox chemistry during the charging and discharging process is represented in Fig. 3a. Another iodine conversion pathway involving a nine-electron transfer process by NaMnI(VII)O_6_ → I_2_ was also reported in the acid–salt water dual electrolytes [62]. However, this conversion is not reversible, limiting its applicability. A novel high potential I_2_/IO_3_^−^ redox couple was also observed in the overcharge process of Zn-I_2_ batteries, but the limited adsorption of IO_3_^−^ on carbon host and its relatively high solubility cause active material loss [63]. The multivalent nature of iodine enables multi-electron transfer processes, which could enable high energy density Zn-I_2_ batteries.

### Zinc-Bromine Battery

Zinc-bromine batteries are among the most widely explored and commercially used Zn-halogen systems owing to their high energy density, low cost, and scalability [64]. The standard cell voltage of the Zn-Br_2_ battery is 1.8 V, which is relatively high among aqueous battery systems [50]. The following reaction takes place at the cathode in ZBBs:

10 $${\mathrm{Cathode}}:\;\;\;{\mathrm{2Br}}^{ - } \leftrightarrow {\mathrm{Br}}_{2} + {\mathrm{2e}}^{ - } \;\;{\mathrm{E}}^{{\mathrm{o}}} = + 1.07\;{\mathrm{V}}\;{\mathrm{vs}}.\;{\mathrm{SHE}}$$

11 $${\mathrm{Overall}}:\;\;\;{\mathrm{Zn}} + {\mathrm{Br}}_{2} \leftrightarrow {\mathrm{ZnBr}}_{2} \;\;{\mathrm{E}}^{{\mathrm{o}}} = + 1.83\;{\mathrm{V}}\;{\mathrm{vs}}.\;{\mathrm{SHE}}$$

Bromine molecules (Br_2_) can further react to form polybromide ions such as Br_3_^−^, Br_5_^−^, and Br_7_^−^ at the cathode [26].

12 $${\mathrm{nBr}}_{{2}} + {\mathrm{Br}}^{ - } \leftrightarrow {\mathrm{Br}}^{ - }_{{{\mathrm{2n}} + {1} }} \left( {{\mathrm{n}} = {1},{2},{3}, \ldots } \right)$$

In addition, Br_2_ poses significant safety concerns owing to its volatile and toxic nature, which potentially releases harmful vapors into the atmosphere. To address this issue, complexing agents are often employed to stabilize Br_2_, which reduces its vapor pressure and chemical reactivity [65]. Quaternary ammonium salts are often employed as complexing agents for Br_2_ [66,67].

### Zinc-Chlorine Battery

Zn-Cl_2_ batteries are attractive because of their high theoretical capacity (755 mAh g^−1^) and potential (1.36 V vs. SHE) of Cl_2_ [55]. Typical battery chemistry involves oxidation of Cl^−^ at the cathode to produce Cl_2_ gas, which is stored in a tank where it continuously dissolves in water and is cooled to form solid crystalline hydrates (Cl_2_.xH_2_O) during charging. The discharging process involves heating of chlorine hydrates to produce Cl_2_ gas, which is transported to the cathode and gets reduced to Cl^−^ [35,68].

13 $${\mathrm{Cathode}}:{\mathrm{Cl}}_{{{2}({\mathrm{g}})}} + {\mathrm{2e}}^{ - } \leftrightarrow {\mathrm{2Cl}}^{ - }_{{({\mathrm{aq}})}} \,{\mathrm{E}} = {1}.{36}\,{\mathrm{V}}\,{\mathrm{vs}}.\,{\mathrm{SHE}}$$

14 $${\mathrm{Cl}}_{{{2}({\mathrm{g}})}} + {\mathrm{xH}}_{{2}} {\mathrm{O}} \leftrightarrow {\mathrm{Cl}}_{{2}} .{\mathrm{xH}}_{{2}} {\mathrm{O}}$$

15 $${\mathrm{Full}}\,{\mathrm{cell}}:{\mathrm{Cl}}_{{{2}({\mathrm{g}})}} + {\mathrm{Zn}}_{{({\mathrm{s}})}} \leftrightarrow {\mathrm{Zn}}^{{{2} + }}_{{({\mathrm{aq}})}} + {\mathrm{2Cl}}^{ - }_{{({\mathrm{aq}})}} \,{\mathrm{E}} = {2}.{12}\,{\mathrm{V}}\,{\mathrm{vs}}.\,{\mathrm{SHE}}$$

However, Zn-Cl_2_ systems are at an early stage of development, and only a few studies have reported on them. A recent study reports MnO_2_ as redox adsorbent to modulate the electrochemical performance of Zn-Cl_2_ battery. Density functional theory (DFT) calculations reveal that Cl_ads_@MnO_2_ intermediate acts as an effective electron donor, enabling faster electron reduction and improving overall electrode kinetics. As a result, the Zn-Cl_2_@MnO_2_ system delivers a higher discharge voltage of 2.0 V at 2.5 mA cm^−2^ and significantly improved cycling stability of 1000 cycles with an average CE of 91.6% [33]. A recent study by Zhang et al. introduced a high concentration choline chloride (30 m ChCl) aqueous electrolyte to overcome the limited electrochemical stability window and poor chlorine redox utilization in conventional Zn-Cl_2_ batteries. Using N-doped activated carbon cathode and choline chloride aqueous electrolyte, the system delivered a high discharge voltage of 2.2 V, 112.8 mAh g^−1^ capacity, and stable cycling over 3700 cycles [27]. Although still in the early stages of development, Zn-Cl_2_ batteries exhibit promising electrochemical characteristics and could evolve into a competitive technology for future large-scale energy storage.

### Battery Configurations

Zinc-halogen batteries are typically categorized into two structural configurations: flow [39] and static systems [40]. A schematic representation of the flow and static Zn-halogen battery configurations is shown in Fig. 2. Each configuration offers distinct advantages that make it suitable for specific applications. Flow battery systems require two electrolyte reservoirs and pumps, in addition to basic components for the supply of active species to the cathode (catholyte) and anode (anolyte). Flow systems provide enhanced active species mixing through continuous circulation and stirring, which ensures homogeneity, accelerates reaction kinetics, and enhances the electrochemical performance [64]. Static systems have gained attention recently owing to their simplicity and low cost, as they do not require external reservoirs or circulating pumps [26]. Biswas et al. compared the cost of flow and flowless battery configurations using the levelized cost of energy stored ($/kWh/cycle/%) and reported approximately $0.017 for the static configuration versus $0.052 for a flow battery [70].

### Challenges in Zn-Halogen Batteries

**Fig. 4:**
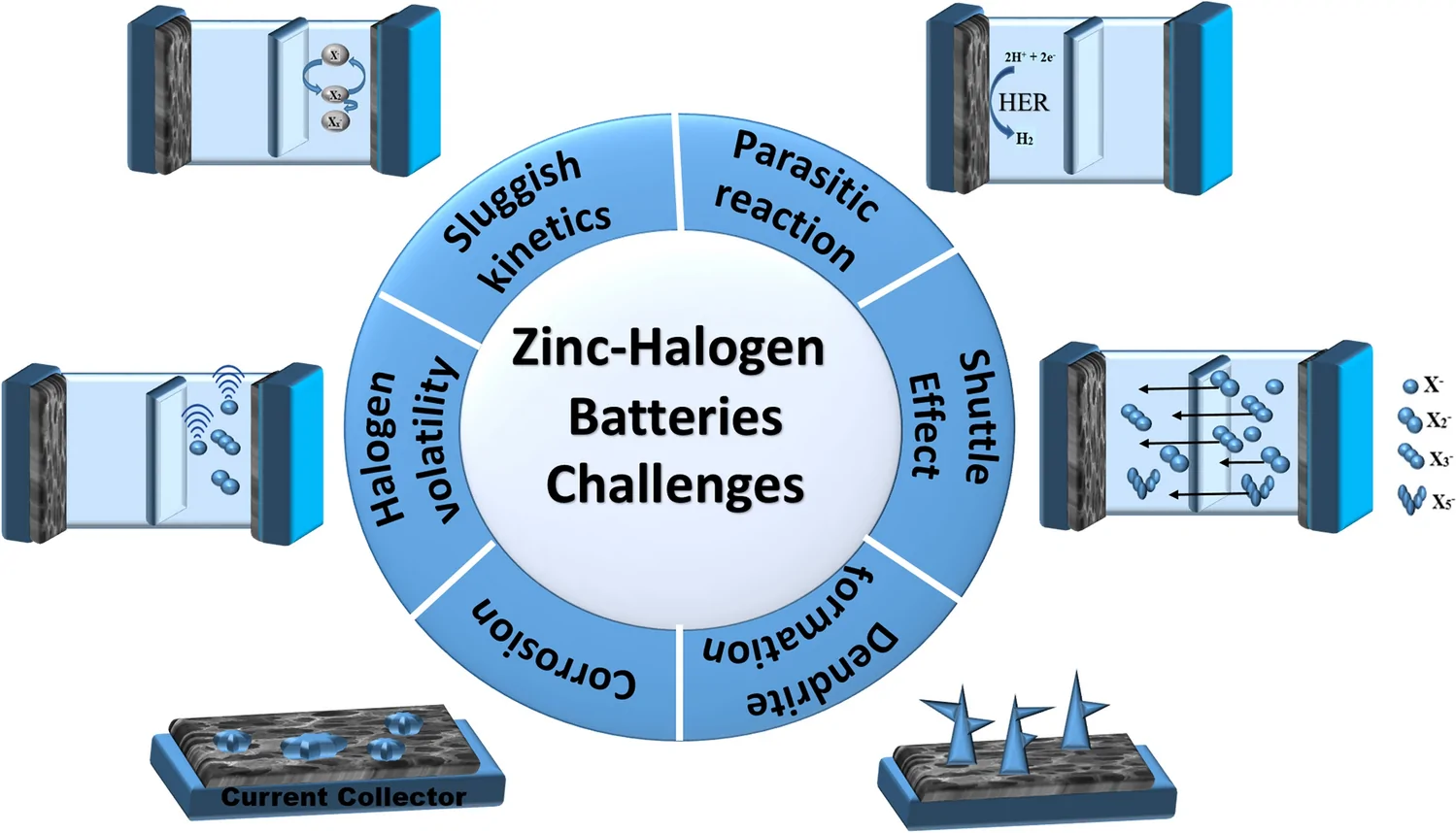
Schematic illustration of key challenges in Zn-halogen batteries

Despite promising energy storage alternatives, Zn-halogen batteries face several fundamental challenges that hinder their practical applications. A schematic illustration of various challenges associated with Zn-halogen batteries is shown in Fig. 4.

#### Shuttle Effect and Halogen Crossover

Shuttling, similar to Li–S batteries, is the most prominent challenge in Zn-halogen batteries [71]. In aqueous zinc-halogen batteries, iodine (I_2_) and bromine (Br_2_) react with halide ions to produce polyiodides (I_3_^−^) and polybromides (Br_3_^−^/Br_5_^−^), respectively [35,72]. These polyhalides migrate from the cathode to the anode in the presence of an electric field and a concentration gradient. This uncontrolled shuttling leads to self-discharge, corrodes the Zn surface, and reduces Coulombic efficiency [73]. This issue is more persistent in Zn-I_2_ batteries because of the high solubility of triiodide (I_3_^−^) [74]. However, the corrosiveness and volatility of Br_2_ are more challenging issues in Zn-Br_2_ batteries, which can corrode the separator and other parts [35].

#### Sluggish Kinetics

In Zn-halogen batteries, the performance is often limited by sluggish kinetics at the cathode. These slow kinetics lead to poor rate performance, polarization losses, reduced voltage efficiency (VE), and low energy efficiency (EE) [72]. The I_2_/I^−^ and redox couples in Zn-I_2_ batteries, and the Br_2_/Br^−^ redox couple in Zn-Br_2_ batteries, suffer from inherently slow electron transfer rates [48,73]. In the Zn-I_2_ battery, the intrinsic insulating nature of the iodine species greatly affects the efficient transfer of electrons [73], while in Zn-Br_2_ batteries, the high volatility and corrosiveness further hinder fast redox reactions [26].

#### Dendrite Formation

The Zn anode, like other anodes (e.g., Li and Na), exhibits the challenge of dendrite formation [6], which is a result of the cumulative process of non-uniform zinc electrodeposition on the Zn anode [75]. During repeated charge–discharge cycles, uneven Zn nucleation and growth on the anode surface led to the formation of needle-like dendritic structures, as shown in Fig. 3b. This occurs because of the higher overpotential required for nucleation compared with growth, which promotes non-uniform deposition [76]. The tip effect further enhances dendrite formation by favoring the deposition of zinc ions at protrusions [77]. Uneven current density and non-uniform electric field are the main driving factors of dendrite formation. These dendrites can penetrate the separator, resulting in a short circuit [39]. In addition, dendrite formation continuously consumes the active material as well as the electrolyte for dendrite formation, which results in a reduced cycle life of the batteries [78].

#### Hydrogen Evolution Reaction

The hydrogen evolution reaction is a prominent side reaction at the Zn anode in Zn-halogen batteries [79]. During the charging process, particularly at higher overpotentials, water can be electrochemically reduced at the Zn anode, leading to the generation of hydrogen gas [26,80]. The HER can be locally accelerated at higher current densities and near the tips of growing dendrites owing to the enhanced electric field and rapid Zn^2+^ consumption at the electrode–electrolyte interface [72,81]. This not only consumes electrons that would otherwise be used for Zn deposition but also results in the loss of electrolyte, active material, and increased internal pressure [82,83].

#### Corrosion

Zinc corrosion, which is closely associated with the HER, is another harmful process in Zn-halogen batteries, in which Zn is irreversibly converted to electrically insulating and inactive precipitates such as Zn(OH)_2_ [84]. These corrosion products form passivation layers that limit ion transport, decrease the effective electroactive surface area, and increase internal resistance [64,85]. In addition, the shuttle effect in Zn-halogen batteries also corrodes the zinc anode [86,87].

## MOFs as Cathode Materials in Zn-Halogen Batteries

**Fig. 5:**
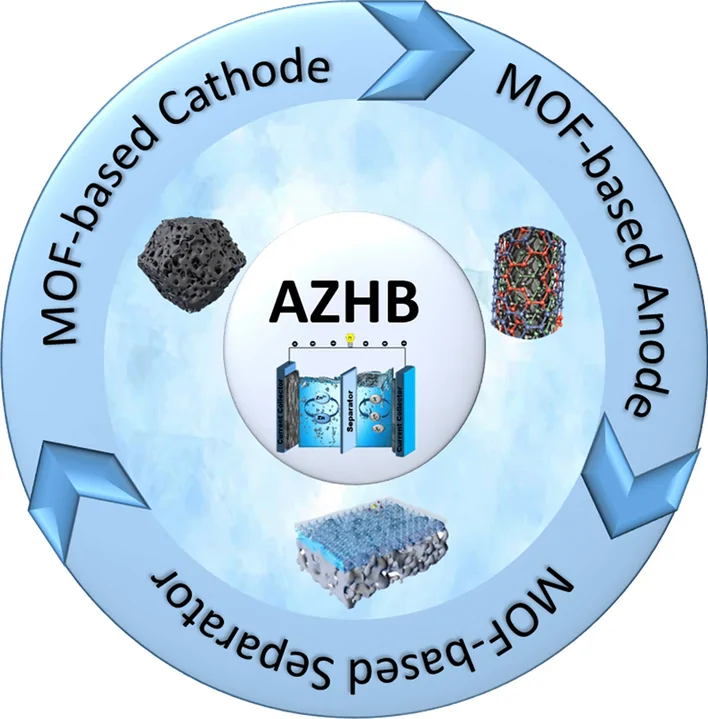
Schematic representation of an aqueous zinc–halogen battery (AZHB) system integrating MOF-based components

Cathode plays an important role in determining the capacity, voltage, and stability of zinc-halogen batteries [88,89]. Halogen-based conversion-type cathodes involving multi-electron transfer processes offer high redox potential, relatively high theoretical capacity, low cost, and safety [90]. These conversion-type energy storage systems face challenges analogous to Li–S systems such as the shuttle effect [91,92]. However, the smaller size and higher solubility of polyhalides as compared to polysulfides demand a host with precise control over pores and functionality. MOFs provide this unique opportunity by providing confinement and catalysis into a single structure [93]. Unlike conventional materials that require post-synthetic modifications, MOFs offer crystalline and tunable scaffolds where pore geometry, chemical binding sites, and catalytic centers can be engineered at the molecular level [94,95]. This intrinsic designability of MOFs has made them a powerful material for Zn-halogen batteries, which can handle problems of various components of battery such as cathode, anode, and separator (Fig. 5).

### Design Principles for MOF-Based Cathodes in Zn-Halogen Batteries

**Fig. 6:**
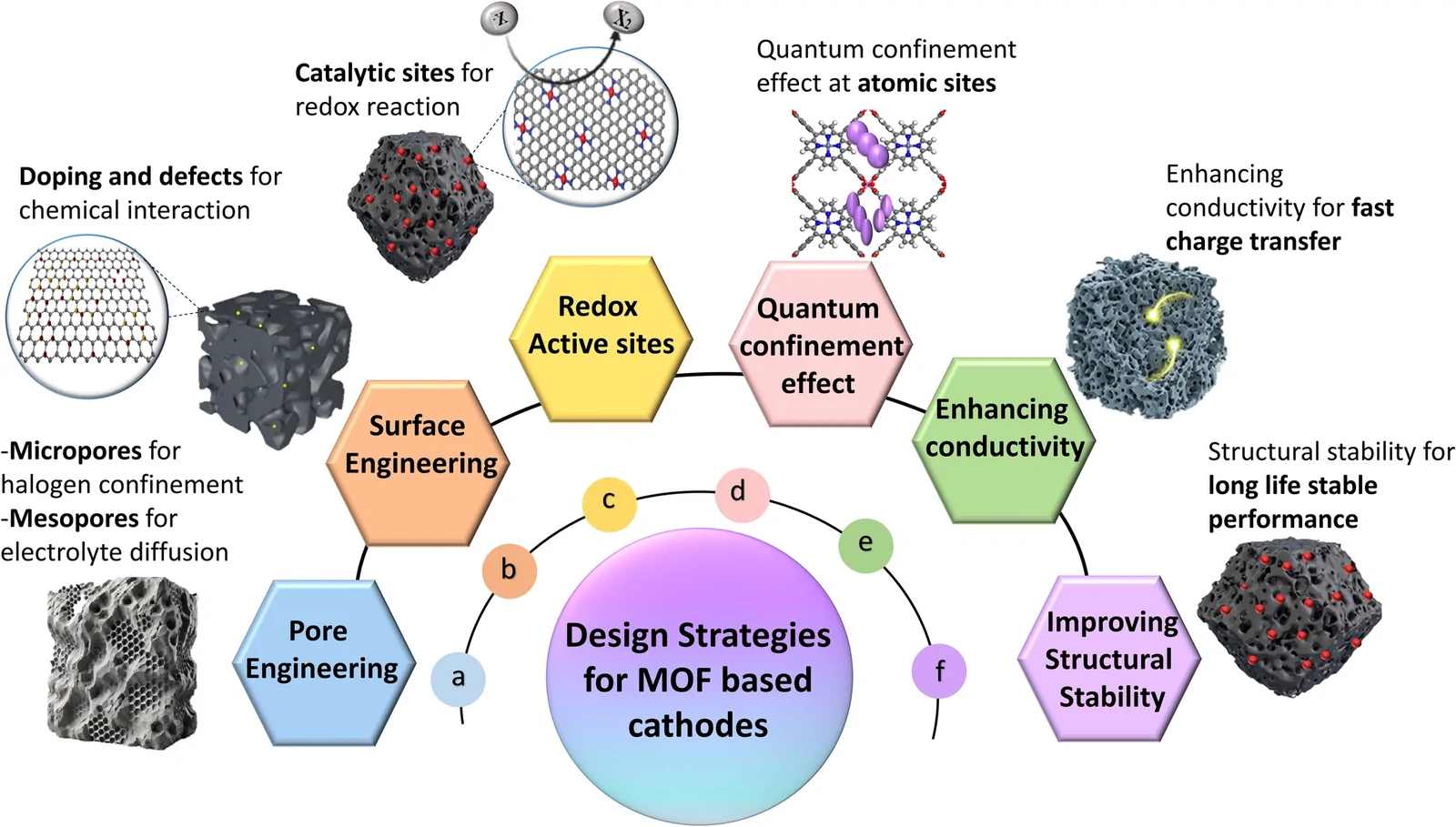
Schematic illustration of key design strategies for MOF-based cathodes

The following are the key design principles that enable MOFs to achieve efficient halogen storage, fast electrochemical reactions, and long-term stability. The key design principles of MOF-based cathodes are summarized in Fig. 6.

#### Tuning Pore Chemistry

The shuttle effect due to the dissolution of halogens in the electrolyte and their movement toward the anode is a major problem at the cathode side of Zn-halogen batteries [74]. Pore engineering plays an important role in physically confining polyhalides within well-defined porous structures [96]. Hierarchical porosity comprising micro-, meso-, and macropores can be tailored to meet the specific requirements of halogen confinement [45,97,98]. The size, type, distribution, and geometry of the pores directly influence the confinement of halogen species. Micropores entrap unwanted polyhalide species (Br_3_^−^ or I_3_^−^) via physical interactions, while mesopores and macropores facilitate rapid electrolyte penetration, providing rapid access to active sites for halogen redox conversion, as highlighted in Fig. 6a [99]. The close pore size matching with halogens to be trapped could facilitate their confinement within pores. Furthermore, synthetic conditions, such as pre-activation in air at low temperatures, introduce significant changes in the surface area and pore size distribution, enabling optimization for specific halogen species. The calcination temperature also has a prominent effect on the pore size distribution. By carefully optimizing the calcination temperature, pores of a suitable size can be developed that can effectively confine iodine species. In conclusion, pore engineering with a suitable size and shape is a vital strategy for the physical confinement of halogen species to mitigate the shuttle effect.

#### Surface Engineering

Surface engineering, including heteroatom doping and surface defects, is an effective strategy for designing MOF-based hosts (Fig. 6b). Due to their high electronegativity, heteroatoms such as N and O polarize the carbon framework, creating localized charge densities that strongly bind halogen or polyiodide species [100]. These heteroatoms not only effectively confine halogen species but also catalyze the redox conversion of halogens [45,101]. The chemisorption at these sites occurs via formation of covalent interactions that enhance the confinement of halogen species, mitigating the shuttle effect, and minimizing self-discharge [45,51]. The synthesis conditions and the type of MOF used have a significant effect on the heteroatomic doping content [102]. A surface containing oxygen functionalities further improves the hydrophilicity of the electrode, resulting in better contact between the electrode and the electrolyte [100]. Optimization of the heteroatom content of porous carbon is crucial for designing Zn-halogen batteries, as it was found that high N doping decreases the surface area, while low N-content can cause framework collapse and weaker interactions with polyhalides [45].

Surface defects and unsaturated coordination sites can modify the local electronic environment, generating localized unstable regions with strong interfacial interactions to enhance adsorption. When these features act in concert with adjacent metal sites, they not only boost the catalytic efficiency but also effectively inhibit undesirable side reactions [103,104].

#### Introduction of Redox Active Sites

Active site engineering is crucial for enhancing the redox kinetics of halogen redox conversion in Zn-halogen batteries, enabling higher active material utilization and improved electrochemical reversibility (Fig. 6c) [31]. By tuning the chemical composition and electronic structure of MOF-derived materials, catalytic sites can be strategically introduced to lower the activation energy for halogen redox reactions and stabilize the intermediate species [51,57,105]. N-doped sites and transition metal centers can effectively catalyze redox reactions and strongly restrict polyhalide species [57,106]. Among the N-dopants, graphitic-N exhibits a low activation barrier and accelerates the redox conversion of halogen species owing to its charge transfer process [106]. Transition metals embedded in N-doped carbon frameworks are particularly effective because they offer active centers that facilitate rapid electron transfer, catalyzing redox conversion. The interaction between the d-orbital of the transition metal and the p-orbital of halogens was also found to influence the binding strength of halogen species and their catalytic conversion [107]. Single-atom catalysts (SACs) have emerged as promising catalysts in the field of rechargeable batteries. The well-defined, uniform structure of the SACs maximized atomic utilization, leading to high performance and conversion efficiency [108]. Additionally, MOFs containing unsaturated metal coordination sites due to high-temperature pyrolysis can also catalyze redox conversion of halogens [48]. In summary, active site engineering in MOF-derived cathodes enhances redox kinetics, increasing the utilization of halogens. This makes it a vital strategy for designing high-performance Zn-halogen batteries.

#### Leveraging Quantum Confinement Effects at Atomic Sites

Beyond pore engineering and introduction of redox active centers, a more profound design principle involves exploiting quantum confinement effects at atomically dispersed metal centers (Fig. 6d) [93,109]. This concept extends beyond the conventional definition of SACs by emphasizing that the specific crystalline and electronic environment of the host framework such as a porphyrinic MOF is critical. When metal atoms are isolated within a defined coordination environment, such as an M-N_4_ moiety in a MOF, the quantum scale confinement induces a significant upward shift of the metal’s *d*-band center. This effect strengthens the adsorption of iodine species via optimized *d-p*-orbital hybridization and significantly reduces the kinetic barrier for polyhalide conversion reactions by stabilizing key intermediates [93]. Therefore, deliberately engineering the host matrix to induce these quantum size effects represents a critical strategy for boosting the kinetics and suppressing the shuttle effect.

#### Enhancing Conductivity in MOF-Based Halogen Hosts

The intrinsically low conductivity of pristine MOFs poses a significant challenge for efficient electron transfer in redox conversion. Efficient charge transfer is essential for improved kinetics in Zn-halogen batteries (Fig. 6e). Various strategies have been reported for enhancing the electrical conductivity of MOFs. One effective approach involves the thermal carbonization of MOFs, which transforms them into a porous carbon network with embedded heteroatoms, significantly enhancing conductivity [45,57,110]. The degree of graphitization increases with increasing carbonization temperature, which contributes to enhanced conductivity [98]. The high graphitic carbon content and porous structure effectively contribute to an increase in the conductivity [110]. Graphitic-N also further improves charge transfer owing to the delocalized π-electron system [45,106]. MOF-derived porous carbons with a high graphitization degree provide efficient electron transport pathways, reduce charge transfer resistance, and minimize voltage polarization [110]. The integration of metals in porous carbon networks also inherently improves electronic conductivity during halogen redox cycling [57]. Another approach is the integration of conductive networks such as polymers within MOF to improve the charge transfer. For example, the intercatenation approach was used to weave MOFs with conductive polymer networks, which significantly improved their electrical conductivity and electrochemical performance. By changing the reactive functional group (− NH_2_) to interpenetrating polypyrrole chains, the conductive network created new electron transport pathways, which increased the conductivity of MIL-68-PPy to 0.00189 µS cm^−1^, 1.27 times increase than that of MIL-68-NH_2_ (0.00149 µS cm^−1^). DFT analysis of such systems has revealed an efficient electron transfer process between the conductive polymer and MOF framework [59]. The incorporation of conductive polymer networks within MOF highlights an important strategy for enhancing conductivity. Another important strategy is the development of conductive 2D conjugated MOFs (NiPPc MOF), which contain in-plane π-delocalization to enhance electron transfer [50]. These approaches highlight important strategies for enhancing conductivity in MOF-based systems.

#### Improving Structural Stability of MOFs

The structural stability of the MOF-derived hosts in Zn-halogen batteries is crucial for ensuring their long-term cyclability (Fig. 6f). Structural degradation, including the collapse of porous framework and active material loss, can lead to capacity fading and poor reversibility during long-term cycling in a Zn-halogen system [48]. Structural stability can be improved through several modification strategies. Introducing hierarchical porosity enhances both the structural and electrochemical stability. The porous structure confines the halogen species and buffers volume changes during redox cycling [45,101,110]. Thermal treatment and doping also play an important role in stabilizing the structure. A higher degree of graphitization reduces structural defects that can propagate under stress conditions and may affect the performance, whereas heteroatom doping can strengthen the carbon framework and anchor halogen species more strongly [98,110]. MOF/polymer composites-derived carbon nanofibers exhibit intrinsic mechanical robustness, resulting in high structural stability and reversibility [99,111]. Furthermore, the composition of the MOF significantly affects its stability. For example, Zr-based MOFs have shown superior chemical stability in weakly acidic electrolytes compared to other metal-based MOF [112].

Collectively, the structural stability is directly linked to the pore structure and chemical composition. Therefore, the structural engineering of MOF-based cathodes is essential for the development of high-capacity and durable Zn-halogen batteries.

Several MOF-based cathodes have been reported based on these design principles and have shown superior performance in Zn-I_2_ and Zn-Br_2_ batteries.

### MOF-Based Materials in Zn-Halogen Batteries

MOF-based materials are widely reported as promising cathodes for Zn-halogen batteries [113]. Through thermal treatment or chemical transformation, they can be converted into a wide range of derivatives, which are primarily classified as carbon-based or non-carbon-based compounds. Carbon-based derivatives, such as nitrogen-doped porous carbons and carbon nanofibers, have strong electrical conductivity, abundant active sites, and high halogen adsorption, effectively suppressing the shuttle effect and increasing redox kinetics [57,98,111]. Non-carbon-based derivatives such as heterostructured composites provide additional catalytic activity, chemical stability, and ion transport channels, thus enhancing the electrochemical performance and cycle stability of Zn-halogen batteries [59,114].

#### MOF-Derived Carbon Hosts

MOF-derived carbon materials serve as multifunctional cathodes for Zn-halogen batteries [57,110,115]. These materials uniquely combine key design strategies to address the challenges of halogen-shuttling and sluggish kinetics. The carbon hosts synthesized by high-temperature pyrolysis of MOF precursors possess a well-defined porous structure, enhanced conductivity, and structural stability [110]. During high-temperature pyrolysis, organic linkers in the MOF carbonize to form a carbonaceous matrix, while metal ions such as Zn evaporate at high temperatures, creating pores [96]. Many MOF-derived carbon cathodes have been explored as efficient hosts for Zn-halogen batteries.

##### Pore-Engineered and Heteroatom-Doped Porous Carbons

**Fig. 7:**
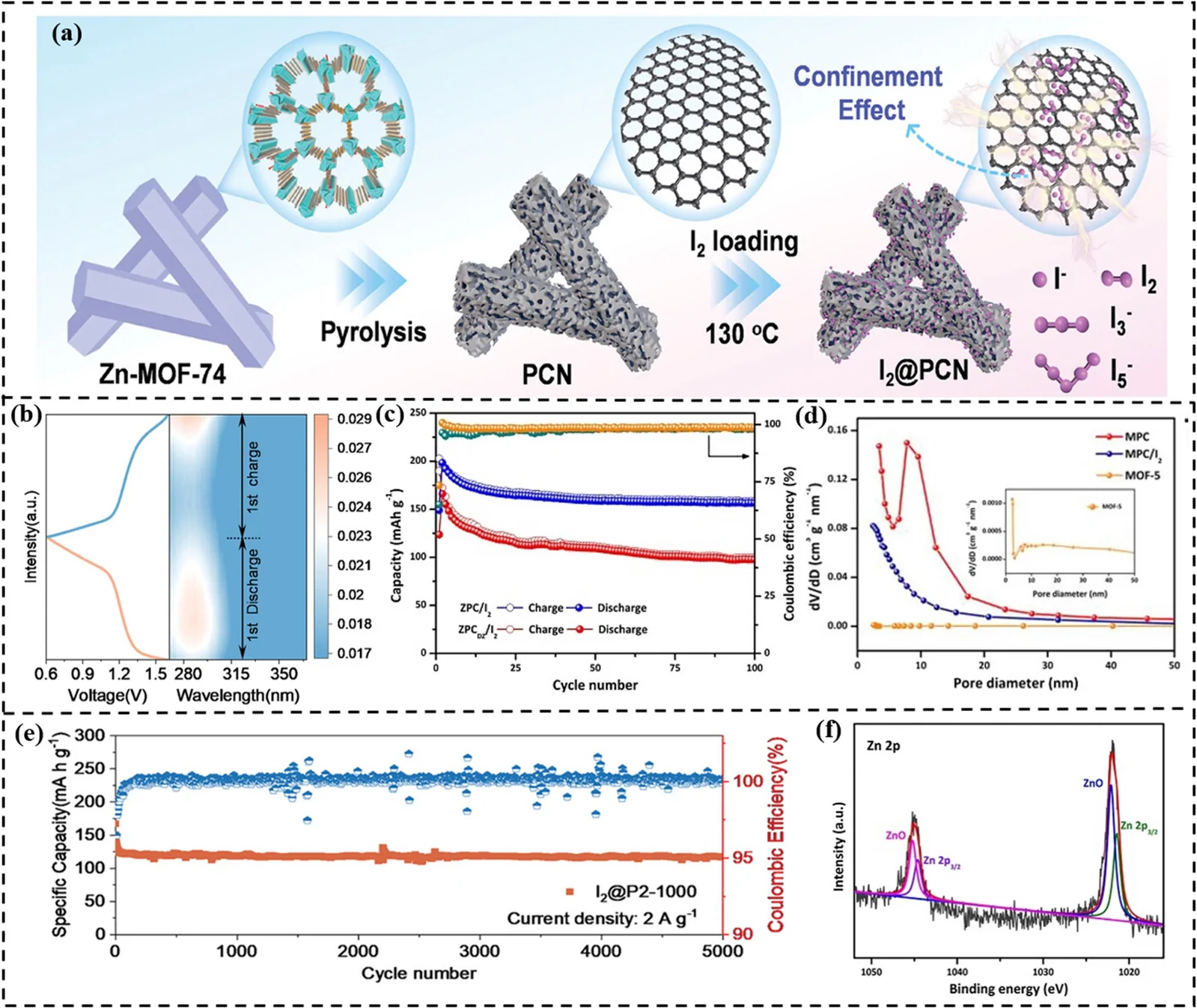
**a** Schematic illustration for preparation of I_2_@PCN [96]. **b** In situ UV–vis spectra of I_2_@P2-1000 in ZnSO_4_ solution [96]. Copyright 2024, Wiley–VCH. **c** cycling stability test of ZPC [97]. Copyright 2022, Elsevier. **d** Pore size distribution of MOF-5, MPC and MPC/I_2_ [116]. Copyright 2023, Elsevier. **e** Cycling stability test of I_2_@P2-1000 [96]. Copyright 2024, Wiley–VCH. **f** XPS spectra of Zn in ZPC/I_2_ [97]. Copyright 2022, Elsevier

The strategic design of pore architecture is critical for physical confinement of halogens. For example, Chai et al. proposed micropores of size 0.66 and 1.25 nm that closely matched the diameter of I^−^ (0.21 nm), I_2_ (0.27 nm), and I_3_^−^ (0.58 nm), facilitating physical confinement of iodine species [110], while pore sizes in the range of 2–10 nm effectively contributed to the effective entrapment of Br species, enhancing Br utilization [100]. Li et al. studied the size confinement strategy effect in Zn-MOF-74-derived porous carbon nanorods (PCN) as iodine hosts (I_2_@PCN) (Fig. 7a) [96]. The rod diameter was tuned by adjusting H_2_O/DMF solvent ratio, while the graphitization degree and conductivity were improved by calcination at three different temperatures: 900, 1000, and 1100 °C. The P2-1000 samples prepared by calcining at 1000 °C contained well-distributed mesopores with an average size of 4.5 nm and a total pore volume of 0.870 cm^3^ g^−1^, while P2-900 and P2-1100 exhibited reduced pore volumes of 0.661 and 0.579  cm^3^ g^−1^, respectively. The porous network of P2-1000 effectively encapsulated the iodine species, restricting their diffusion into the electrolyte and minimizing the shuttle effect. This was confirmed by UV/Vis spectroscopy results, where no peaks of I_3_^−^ at 285 and 353 nm were observed (Fig. 7b). Iodine adsorption ability tests were performed, in which different samples (P2-900, P2-1000, and P2-1100) were immersed in a saturated iodine electrolyte. P2-1000 was rapidly decolorized from a brown iodine solution within 24 h, showing superior ability for iodine confinement. As a result, I_2_@P2-1000 cathode exhibited discharge capacity of 179.9 mAh g^−1^ at 100 mA g^−1^ and long-term cyclability of 5000 cycles (Fig. 7e) [96].

Beyond physical confinement in a porous framework, heteroatom doping is crucial for enhancing chemisorption and catalytic activity. Heteroatom-doped porous carbons contain heteroatoms such as N and P incorporated into the porous carbon framework. ZIF-8-derived porous carbons naturally contain a high content of nitrogen functionalities due to the decomposition of 2-methylimidazole linker. For example, in comparison with MOF-5, porous carbons derived from ZIF-8 such as ZPC/I_2_ showed better performance owing to self-N-doping. ZPC/I_2_ cathode used in Zn-I_2_ battery delivered specific capacity of 192 mAh g^−1^ at a current of 100 mA g^−1^ and retained 156 mAh g^−1^ with capacity retention of 79% after 100 cycles (Fig. 7c). This is attributed to abundant micro-, mesopores (7.9 nm) and active sites (N, ZnO) which effectively suppressed shuttle effect and enhanced conductivity [97]. XPS spectra of Zn are shown in Fig. 7f, which shows the presence of ZnO, whereas MOF-5-derived mesoporous carbon-loaded iodine (MPC/I_2_) cathode showed a reversible capacity of 137 mAh g^−1^ after 300 cycles at 0.1 A g^−1^. The mesopores of MPC/I_2_ provided some confinement, but it was insufficient to fully suppress the polyiodide shuttle effect. The pore size distribution of MPC/I_2_ is shown in Fig. 7d [116]. Furthermore, MOF-5-derived carbon only exhibited mesopores, while ZIF-8-derived carbon has both micro- and mesopores that matched the size of the iodine species, effectively suppressing the shuttle effect while allowing electrolyte penetration.

**Fig. 8:**
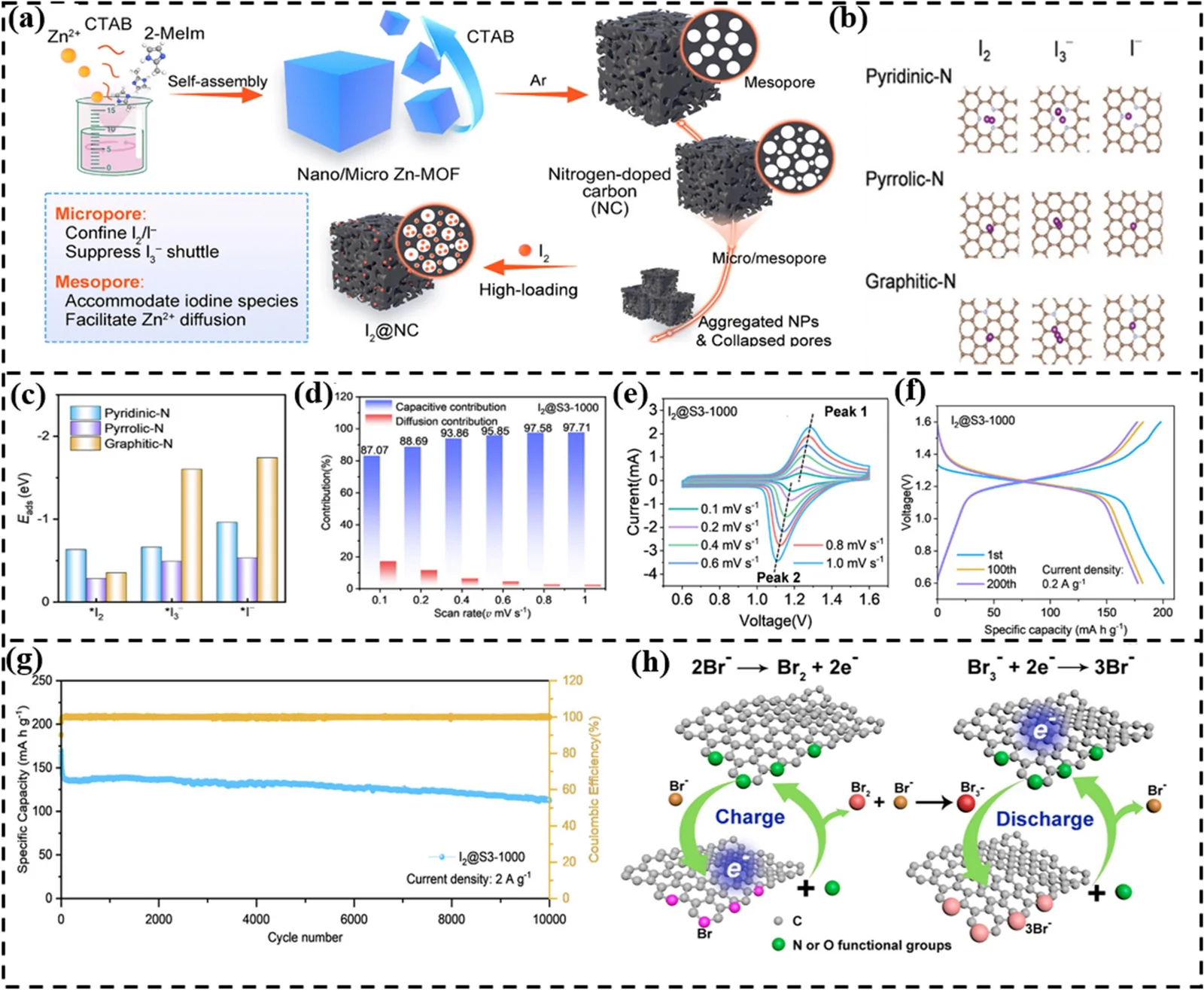
**a** Schematic diagram for the synthesis of Zn-MOF-derived NC, **b** model showing interaction of iodine species with different types of nitrogen, **c** adsorption energies of different iodine species calculated by DFT, **d** percentage capacitive contribution, **e** CV curve, **f** charge–discharge curve, **g** cycling performance of I_2_@S3-1000 [45]. Copyright 2025, Wiley–VCH, **h** catalytic mechanism of the bromine redox reaction on PNSC [100]. Copyright 2018, Elsevier

In another study, nano-/micro Zn-MOF-derived nitrogen-doped porous carbon (NC) was used as the cathode in a Zn-I_2_ battery (Fig. 8a). The inherent porous structure ensured high conductivity, facilitating fast electron transfer and a high iodine loading of 43.7 wt%. DFT analysis revealed the distinct roles of different N-types (Fig. 8b): Graphitic-N exhibited strongest adsorption for I^−^ and I_3_^−^ due to its planar coordination while pyridinic-N served as the primary capture site (Fig. 8c). This synergistic effect, where pyridinic-N captures and adjacent graphitic-N stabilizes and catalyzes conversion, resulted in excellent performance. The I_2_@S3-1000 cathode delivered a high initial discharge capacity of 200.5 mAh g^−1^, maintained 177.7 mAh g^−1^ after 200 cycles (Fig. 8f), and showed enhanced kinetics with 97.71% capacitive contribution (Fig. 8d), as determined from its CV curve (Fig. 8e). It also demonstrated remarkable long-term stability, retaining 112.4 mAh g^−1^ after 10,000 cycles at 2 A g^−1^ with a minimal decay rate of 0.004% per cycle (Fig. 8g) [45].

**Fig. 9:**
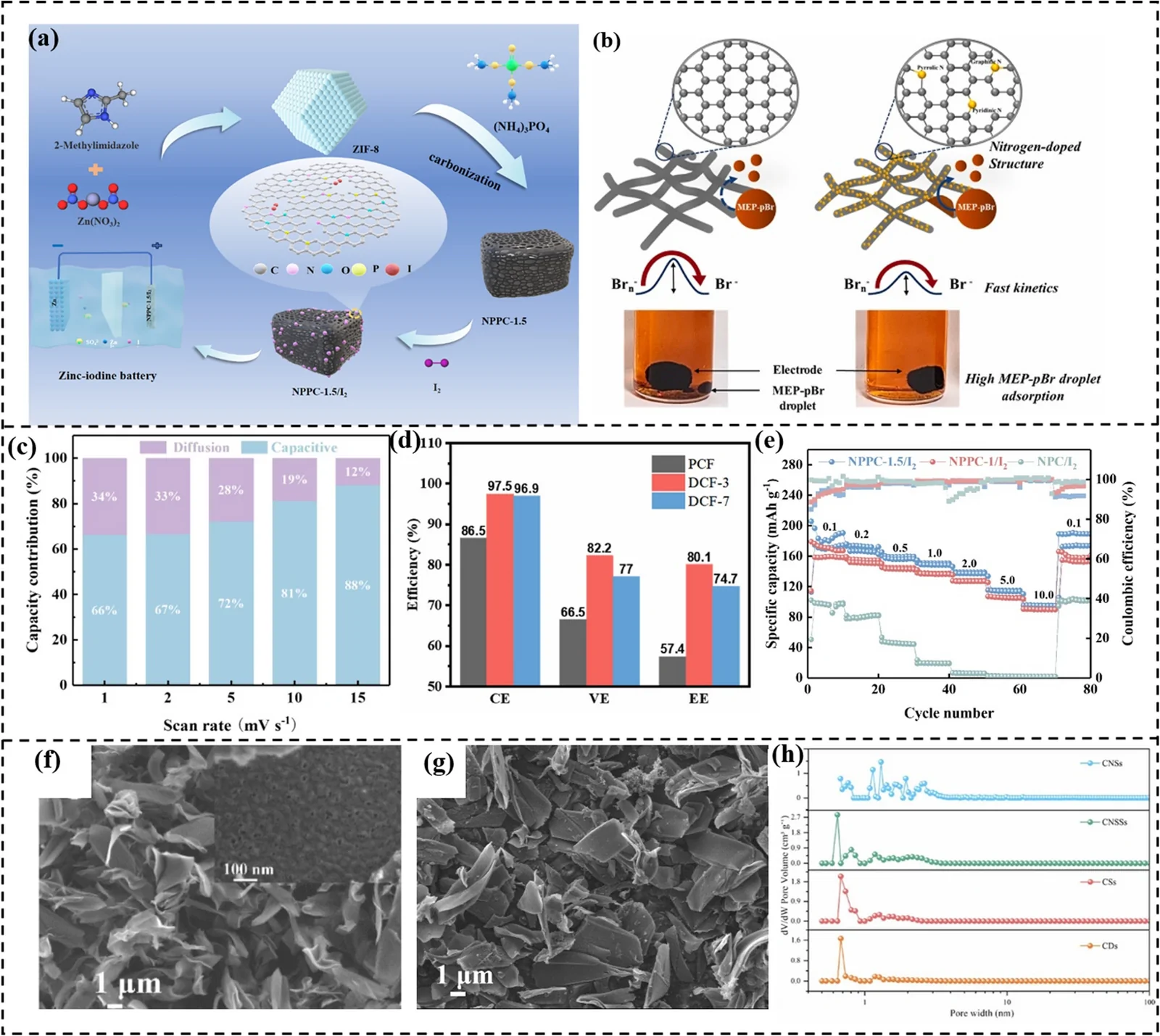
**a **Schematic illustration showing synthesis of NPPC-1.5/I_2_ [101]. Copyright 2024, Elsevier. **b** Schematic illustration showing adsorption of MEP-pBr [66]. Copyright 2024, Elsevier. **c** Capacitive contribution percentage of NPPC-1.5/I_2_ cathodes at different scan rates [101]. Copyright 2024, Elsevier. **d** CE, VE, EE of PCF, DCF-3 and DCF-7 [66]. Copyright 2024, Elsevier. **e** Rate performance of NPPC-1.5/I_2 _[101]. Copyright 2024, Elsevier. **f** Morphology of PNSC before cycling and **g** after cycling [101]. Copyright 2018, Elsevier. **h** Pore size distribution of CNSs, CNSSs, CSs, CDs [102]. Copyright 2023, Elsevier

N-doped porous carbons also showed good performance for Zn-Br_2_ batteries. Wang et al. synthesized porous nano-sheet carbon (PNSC) from zeolite-type MOFs for Zn-Br_2_ redox flow battery. PNSC contained abundant micropores, mesopores, and nitrogen content (pyridinic-N, graphitic-N, and pyrrolic-N), which polarize carbon to enhance bromine adsorption (due to the high electronegativity of N) and catalyze redox activity for Br conversion. In addition, O at the surface produces a stable electrode–electrolyte interface. The schematic mechanism of the bromine redox reaction catalysis on the PNSC is shown in Fig. 8h. The nanosheet morphology significantly shortened the electron pathway and enhanced electronic conductivity. The post-synthetic CO_2_ activation process generated in-plane pores and a highly porous, loosely packed structure, which created 3D pathways, leading to improved ion diffusion throughout the framework. Synergistic effect of porosity, N-doping, and improved electron/ionic conductivity. PNSC achieved 83% VE and 82% EE at a current density of 80 mA cm^−2^. The nanosheet morphology was also preserved after 200 cycles, indicating its structural stability as shown in Fig. 9f, g [100]. In Zn-Br_2_ flow batteries, bromine sequestration agents (BSA) are used to reduce Br crossover and volatility, but this limits mass transport and significantly increases the overpotential. The adsorption of BSA-pBr droplets on the electrode surface improves battery performance [117]. Lee et al. demonstrated that N-doped defective carbon felts (DCF) derived from ZIF-8 could facilitate the adsorption of N-methyl N-ethyl pyrrolidinium MEP-pBr complexes on the electrode surface (Fig. 9b). DCF with graphitic-N-rich surfaces significantly enhanced the adsorption of MEP-pBr droplets, significantly boosting the kinetics and decrease the crossover of bromine bearing species. This modification simultaneously mitigated bromine-induced corrosion. Consequently, the battery performance improved with 79% EE over 900 cycles at 100 mA cm^−2^ (Fig. 9d) [66].

The synergistic effect of multi-heteroatom doping is shown in N, P co-doped porous carbon (NPPC) using ZIF-8 as a precursor and (NH_4_)_3_PO_4_ as an activating agent (Fig. 9a). (NH_4_)_3_PO_4_ decomposed to form H_3_PO_4_ and NH_3_, where H_3_PO_4_ acts as a pore-forming agent and a doping source for phosphorus, whereas NH_3_ reacts with carbon during calcination to produce N functionalities. The as-prepared NPPC contained high nitrogen (21.4 wt%) and phosphorous (18.4 wt%) doping contents, which effectively trapped polyiodide species by chemisorption, thereby increasing the hydrophilicity of the electrode. NPPC-1.5/I_2_ delivered a specific capacity of 175 mAh g^−1^ at 0.1 A g^−1^ and 95 mAh g^−1^ at 10 A g^−1^, demonstrating good rate performance, as shown in Fig. 9e. The increasing capacitive contribution of NPPC-1.5/I_2_ with an increasing scan rate also highlights its good kinetics (Fig. 9c). The cycling stability test confirmed the superior performance of NPPC-1.5/I_2_ as it retained 97% of its initial capacity after 6000 cycles at 10 A g^−1^ [101]. However, nano-/micro-Zn-MOF-derived I_2_@S3-1000 exhibited better performance than NPPC-1.5/I_2_.

A change in the synthesis method changes the morphology of the resulting material, which affects its properties and performance. Sun et al. synthesized heavily heteroatom-doped carbon with tunable microstructures. Different morphologies of porous carbons (carbon nanosheets CNSs, carbon nanoshells CNSSs, carbon skeletons CSs, and carbon dodecahedrons CDs) were derived by changing additives. The direct pyrolysis of ZIF-8 preserved the dodecahedral morphology, whereas the addition of CaCl_2_ formed CSs. The CsCl-NaCl eutectic salt produced hollow CNSSs, while the combination of both CaCl_2_ and CsCl-NaCl salts produced CNSs. The resulting morphologies maintained a high heteroatomic content in the range of 20.8–33.8 wt%. CNSs possessed both micropores and mesopores that were favorable for ion storage and ion diffusion, whereas CNSSs, CDs, and CSs exhibited only micropores (Fig. 9 h). As a result, CNSs/I_2_ showed a high capacity of 313.6 mAh g^−1^ at 0.5 C and demonstrated excellent rate capability by maintaining 170.5 mAh g^−1^ at a very high current density of 100 C. CNSs/I_2_ also showed good cycling stability with 85% retention rate after 6000 cycles at 20 C. The devices based on CNSs/I_2_ can achieve a specific energy of 219.5 Wh kg^−1^ at a specific power of 70 W kg^−1^ [102].

**Fig. 10:**
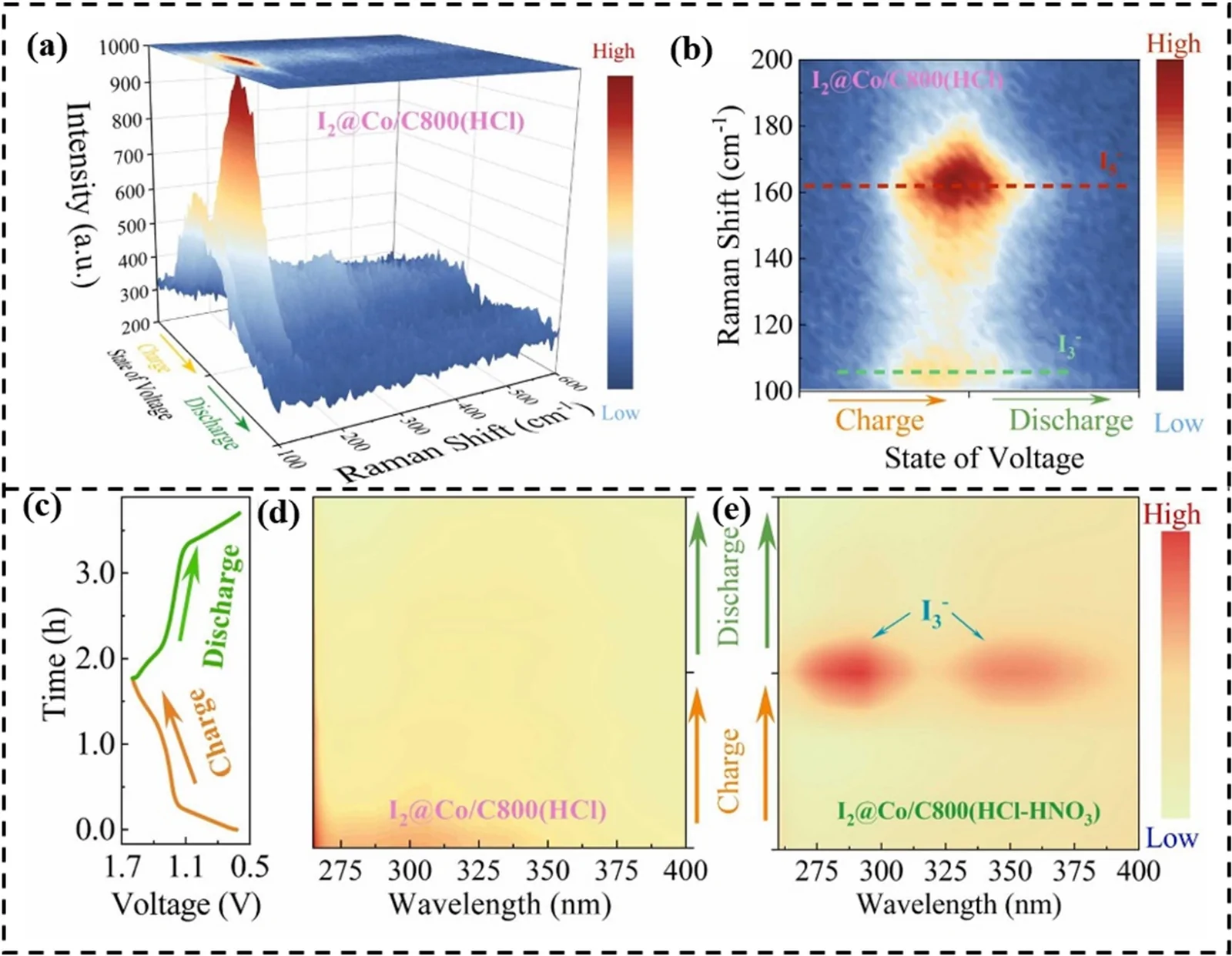
**a, b** In situ Raman spectrum during the charge/discharge process with cathode I_2_@Co/C800(HCl), **c** the charge/discharge profiles during the in situ UV–vis absorption spectroscopy experiment, **d, e** in situ UV–vis absorption spectroscopy for charge/discharge process with cathode: **d** I_2_@Co/C800(HCl) and **e** I_2_@Co/C800(HCl-HNO_3_) [118]. Copyright 2024, Elsevier

The strategic incorporation of both metal species and nitrogen dopants in MOF-derived carbon matrices creates a synergistic effect that enhances the electrochemical performance of Zn-halogen batteries. For example, Co/N co-doped carbon derived from ZIF-67 after carbonization and acid treatment (I_2_@Co/C800(HCl)) served as a bifunctional host in the Zn-I_2_ battery. MOF-derived carbon provided a high surface area and porosity, N-sites provided strong polar adsorption ability to polyiodides, and Co sites effectively catalyzed iodine redox reaction. In situ Raman analysis of the electrode, as shown in Fig. 10a, b, shows two peaks at 110 and 160 cm^−1^ during charging, attributed to I_3_^−^ and I_5_^−^ anions, respectively, while in situ UV/Vis analysis of the electrolyte (Fig. 10c-e) indicates no I_3_^−^ peak in the electrolyte for I_2_@Co/C800(HCl), but I_2_@Co/C800(HCl-HNO_3_) exhibited an I_3_^−^ peak. This shows complete inhibition of the shuttle effect in I_2_@Co/C800(HCl). As a result, the Zn-I_2_ battery exhibited a specific capacity of 152 mAh g^−1^ at 5 C and cycling stability of 24,000 cycles with 80% capacity retention [118].

**Fig. 11:**
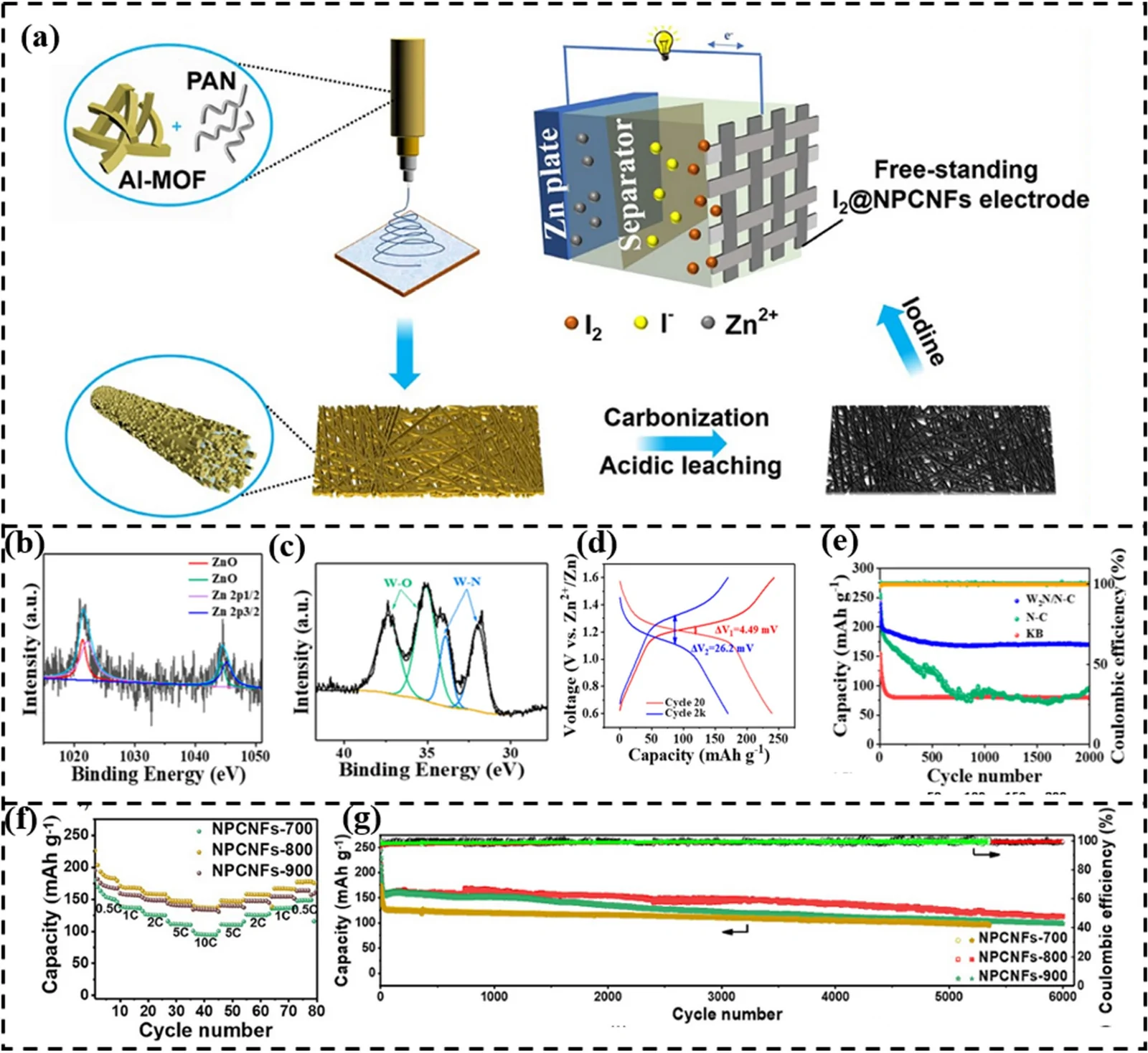
**a** Schematic illustration of synthesis of NPCNFs electrode [99]. Copyright 2022, Springer Nature. High-resolution XPS spectra of: **b** Zn 2*p*, **c** W 4*f* of W_2_N/N–C, **d** GCD curve and **e** cycling test of inW_2_N/N–C [119]. Copyright 2023, American Chemical Society. **f** Rate capability test and **g** cycling stability test of different NPCNFs [99]. Copyright 2022, Springer Nature

Metal nitride nanoparticles incorporated into a porous carbon framework offer robust active sites for halogens. For instance, tungsten nitride nanoparticles within N-doped carbon (W_2_N/N–C) were synthesized in situ by carbonizing phosphotungstic acid-loaded ZIF-8 (PTA@ZIF-8). The ZIF-8 enabled a structure with hierarchical porosity and N-doping for effective iodine confinement. Residual Zn and ZnO species, confirmed by XPS (Fig. 11b), enhanced conductivity and chemisorption. Furthermore, the metal–nitrogen bonds induced d-band shrinkage (Fig. 11c), lowering the activation energy for iodine redox reactions. As a result, the I_2_@W_2_N/N–C cathode delivered a high discharge capacity of 200.9 mAh  g^−1^ at 5 C (Fig. 11d) and retained 85% of its capacity over 2000 cycles (Fig. 11e) [119].

##### Advanced Carbon Architectures and Defect Engineering

Beyond heteroatom doping, the morphology of MOF-derived carbons also significantly impact their performance. Engineering advanced carbon architectures such as 1D carbon nanofibers, hollow hierarchical porous carbon and defect-rich network facilitates mass transport, enhance conductivity, and provide more robust active sites. For example, electrospun MOF-derived carbon nanofibers (CNFs) have emerged as a promising class of materials owing to their hierarchical porous structure, superior conductivity, and structural stability [120]. Electrospinning of MOF/polymer composites followed by carbonization yields self-supported CNFs that address key challenges, such as sluggish kinetics, shuttle effect, and poor long-term stability. For example, N-doped porous carbon nanofibers (NPCNFs) derived from Al-MOF/PAN composites (Fig. 11a) demonstrate a highly ordered porous network consisting of abundant micropores and mesopores and a nitrogen-rich carbon matrix. The resulting I_2_@NPCNFs-800 efficiently confined iodine intermediates within pores through physical interactions, while N-sites, especially pyridinic and graphitic N, further chemically confined iodine species, suppressing the shuttle effect and increasing active material utilization. I_2_@NPCNFs-800, thus demonstrated small voltage polarization, high reduction potential (1.25 V), good rate performance (184.3 mAh g^−1^ at 0.5 C and 138.9 mAh g^−1^ at 10 C), and stable cycling with 99.7% CE after 6000 cycles. The rate capability and cycling stability tests of NPCNFs electrodes carbonized at different temperatures are shown in Fig. 11f, g [99]. Wan et al. also synthesized Fe/N co-doped carbon nanofibers derived from Fe/Zn-ZIF/PAN/PVP composite for Zn-Br_2_ systems. The resulting structure exhibited a high surface area and well-developed multilevel pore structure comprising both micro- and mesopores, attributed to the decomposition of Zn and PVP during carbonization. The porous structure increases the accessibility of active sites and enhances mass transfer and electrolyte contact, thereby increasing bromine redox conversion. The fibrous structure also exhibited enhanced conductivity, minimal charge transfer resistance, and improved mechanical robustness. Fe/Zn-ZIF/PAN/PVP with 4% Fe exhibited a high CE of 98%, VE of 82.4%, and EE of 81% [111].

**Fig. 12:**
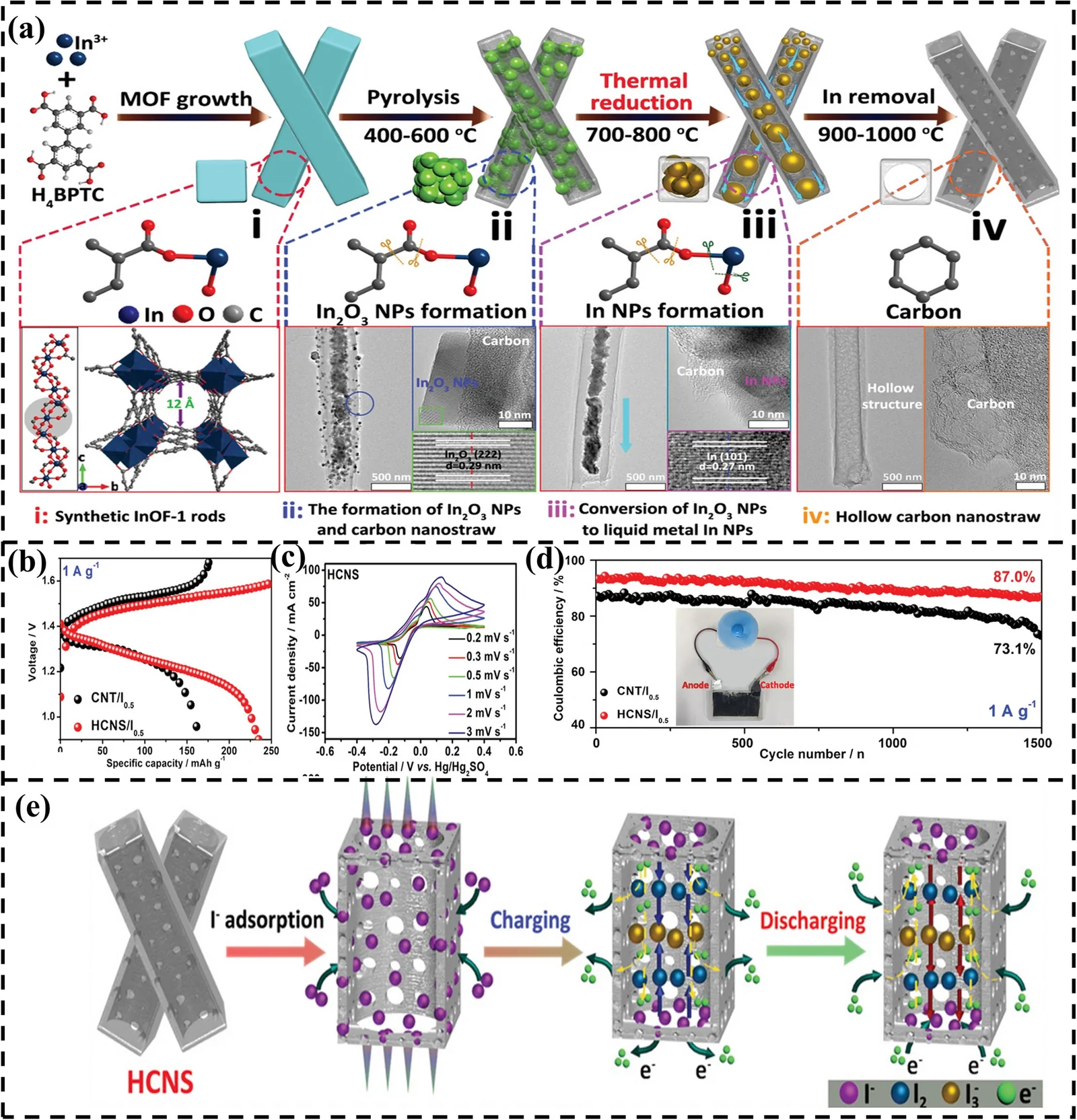
**a** Schematic representation of the synthesis process for hierarchically porous HCNS derived from InOF-1, showing their distinct carbonization stages at varying temperatures, **b** voltage profiles of HCNS/I_0.5_ under different current densities. **c** CV profile of HCNS, **d** cycling stability test of HCNS at 1 A g^−1^, **e** schematic illustration of charge storage mechanism in HCNS during charging/discharging process [110]. Copyright 2022, Wiley–VCH

Hierarchical hollow carbon nanostraws (HCNS) present an advanced structural platform for addressing challenges in Zn-halogen batteries. They are hollow and have porous walls with nanostraw morphology. Chai et al. synthesized a unique hollow carbon structure by high-temperature carbonization of InOF-1 nanorods, where In_2_O_3_ nanoparticles were initially formed and subsequently reduced to metallic indium. These metal nanoparticles gradually fuse and migrate outward, eventually volatilizing (owing to the low melting point of indium), resulting in the formation of hollow carbon nanostraw characterized by a hollow core and porous walls. A schematic illustration of the synthesis of HCNS with their morphologies at different stages is shown in Fig. 12a. The resulting HCNS possessed additional micro- and mesopores in addition to the original pores of the MOF, which physically confines the iodine species while the hollow structure offers an efficient pathway for electron/ion transport and buffer volume changes during redox reactions. The charge storage mechanism in HCNS during the charging/discharging process is shown in Fig. 12e. In addition, the conductive carbon framework facilitates a rapid charge transfer and high active material utilization. As a result, HCNS exhibited a very low charge transfer resistance (R_ct_, 1.9 Ω). The zinc iodine battery assembled using HCNS/I_0.5_ demonstrated a high capacity of 234.1 mAh g^−1^ at 1 A g^−1^ and a CE of 87% after 1500 cycles (Fig. 12d). The GCD profile of HCNS in comparison with CNT is shown in Fig. 12b, whereas Fig. 12c shows the CV profile of HCNS at different scan rates [110].

Defect-engineered carbon hosts are an advanced class of MOF-derived materials that effectively suppress the shuttle effect. Ye et al. reported defect-engineered ZIF-8-derived carbon host (Ni/Zn bimetallic anchored on self-N-doped carbon) synthesized by pre-activation in air at low temperature (300 °C) followed by high-temperature carbonization (NZ-aNC). This pre-treatment disrupted metal coordination bonds, creating defects, and enlarged pores for effective iodine confinement. The defect sites, including oxygen vacancies, broken Zn-N bonds, and N-doped edge sites, formed highly active regions that strongly anchored iodine species and synergistically worked with embedded metal atoms to catalyze the redox process. This design yielded superior performance with a high specific capacity of 219 mAh g^−1^ at 5 A g^−1^ and ultralong cycling stability with 95% capacity retention over 20,000 cycles [98]. These findings highlight an important strategy for introducing defects in the carbon framework to enhance the performance of Zn-halogen batteries.

##### Single-Atom Catalysts (SACs)

**Fig. 13:**
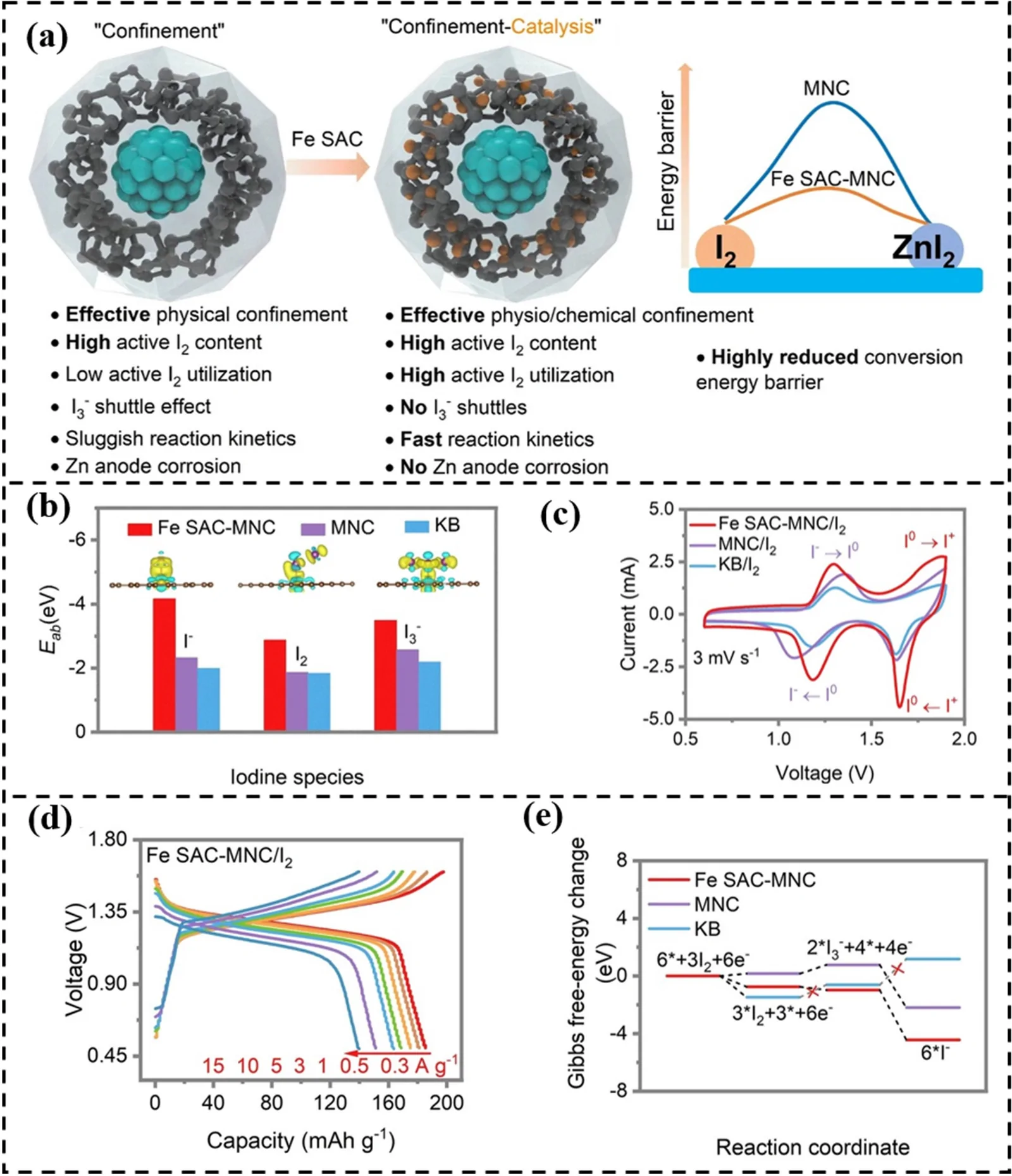
**a** Schematic illustration of confinement catalysis approach of Fe SAC-MNC. **b** Adsorption energies of iodine species on Fe SAC-MNC, **c** CV curve, **d** charge–discharge curve and **e** Gibbs free energy of Fe SAC-MNC [105]. Copyright 2023, Springer Nature

SACs embedded in porous carbon frameworks have emerged as promising electrocatalysts because of their high atomic utilization, well-defined active sites, and tunable coordination environments [121]. In Zn-halogen batteries, MOFs-derived atomically dispersed SACs provide dual benefits: They exhibit strong affinity toward polyhalide species, suppressing the shuttle effect while catalyzing redox conversion by lowering the activation energy barrier and improving reversibility [51,105]. During pyrolysis, metal atoms from the MOF nodes or introduced precursors can be stabilized as single-atom sites within N-doped carbon matrices derived from MOFs such as ZIF-8 [74,122]. Fe SAC embedded in porous N-doped carbon (Fe SAC-MNC) derived from nanoemulsion UiO-66-NH_2_ was used as the cathode for the Zn-I_2_ battery. It served as a confinement–catalysis host with abundant pores in the carbon framework for physical confinement. A schematic illustration of the confinement–catalysis approach used in the Fe SAC-MNC is shown in Fig. 13a. The cathode exhibited highly reversible iodine adsorption kinetics as represented by the adsorption energies of the iodine species in Fig. 13b. The Fe single atoms lowered the activation energy barrier, catalyzing the redox conversion of iodine, as evident in the Gibbs free energy diagram of Fe SAC-MNC in Fig. 13e. The carbon framework and metallic Fe also contributed to the enhanced conductivity, lowering the charge transfer resistance. As a result, the Fe SAC-MNC exhibited a high capacity of 188.2 mAh g^−1^ at 0.3 A g^−1^, good rate performance with a capacity of 139.6 mAh g^−1^ at 15 A g^−1^, and excellent cycling stability over 50,000 cycles with 80.5% capacity retention. Figure 13d represents its GCD profile at various current densities [105].

**Fig. 14:**
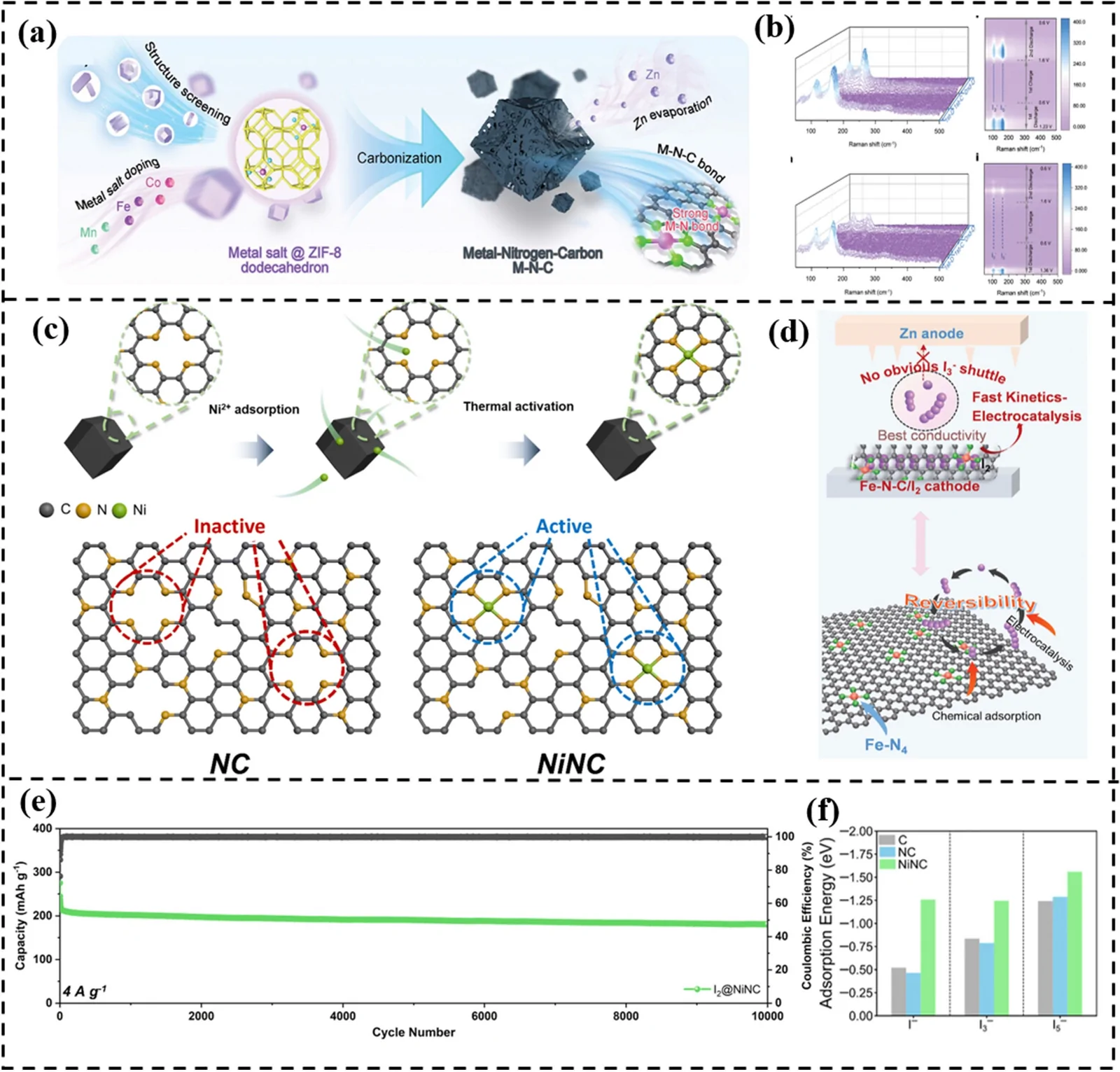
**a** Schematic diagram representing preparation of Fe–N-C [57], **b** in situ Raman spectra of M9/I_2_ [57]. Copyright 2024, Wiley–VCH. **c** Schematic illustration of preparation of Ni single atoms anchored on N-doped carbon [123]. Copyright 2024, Royal Society of Chemistry. **d** Schematic illustration of iodine redox reaction on Fe–N-C [57]. Copyright 2024, Wiley–VCH. **e** Cycle test of NiNC and **f** adsorption energies of iodine species on NiNC [123]. Copyright 2024, Royal Society of Chemistry

Guo et al. developed ZIF-8-derived porous Fe–N-C, in which Fe-N_4_ was atomically dispersed. The host exhibited physicochemical interactions with iodine species, with Fe-N_4_ exhibiting a strong tendency for covalent bonding with iodine species. The resulting cathode demonstrated (M9/I_2_) a high capacity of 161.9 mAh g^−1^ at 2 A g^−1^ for 10,000 cycles. A conceptual diagram representing the synthesis of Fe–N-C from ZIF-8 is shown in Fig. 14a, d shows the iodine redox reaction chemistry on Fe–N–C, and Fig. 14b shows the in situ Raman spectra of M9/I_2_, which represents strong confinement of iodine species on M9/I_2_ [57]. Furthermore, ZIF-8-derived carbon possessed a higher surface area, micro-, and mesopores that improved the conductivity and confinement [57,105]. Lee et al. synthesized Ni single-atoms incorporated into N-doped carbon (NiNC), which exhibited abundant micropores and atomically dispersed Ni sites coordinated to pyridinic-N sites (Ni-N_4_), which otherwise acted as inactive centers. The synthesis of the NiNC by thermal activation of dormant N sites is shown in Fig. 14c. NiNC showed superior chemisorption of iodine species, reducing the shuttling effect. The superior adsorption energies of iodine species on NiNC compared to those on NC and C are shown in Fig. 14f. As a result, Ni-SAs produced by activation of dormant nitrogen sites (I_2_@NiNC) can deliver a reversible specific capacity of 239 mAh g^−1^ at 0.5 A g^−1^ and 193 mAh g^−1^ at 6 A g^−1^, demonstrating their rate performance. The cycling stability test of I_2_@NiNC, illustrated in Fig. 14e, showed 82% capacity after 10,000 cycles at 4 A g^−1^ [123].

**Fig. 15:**
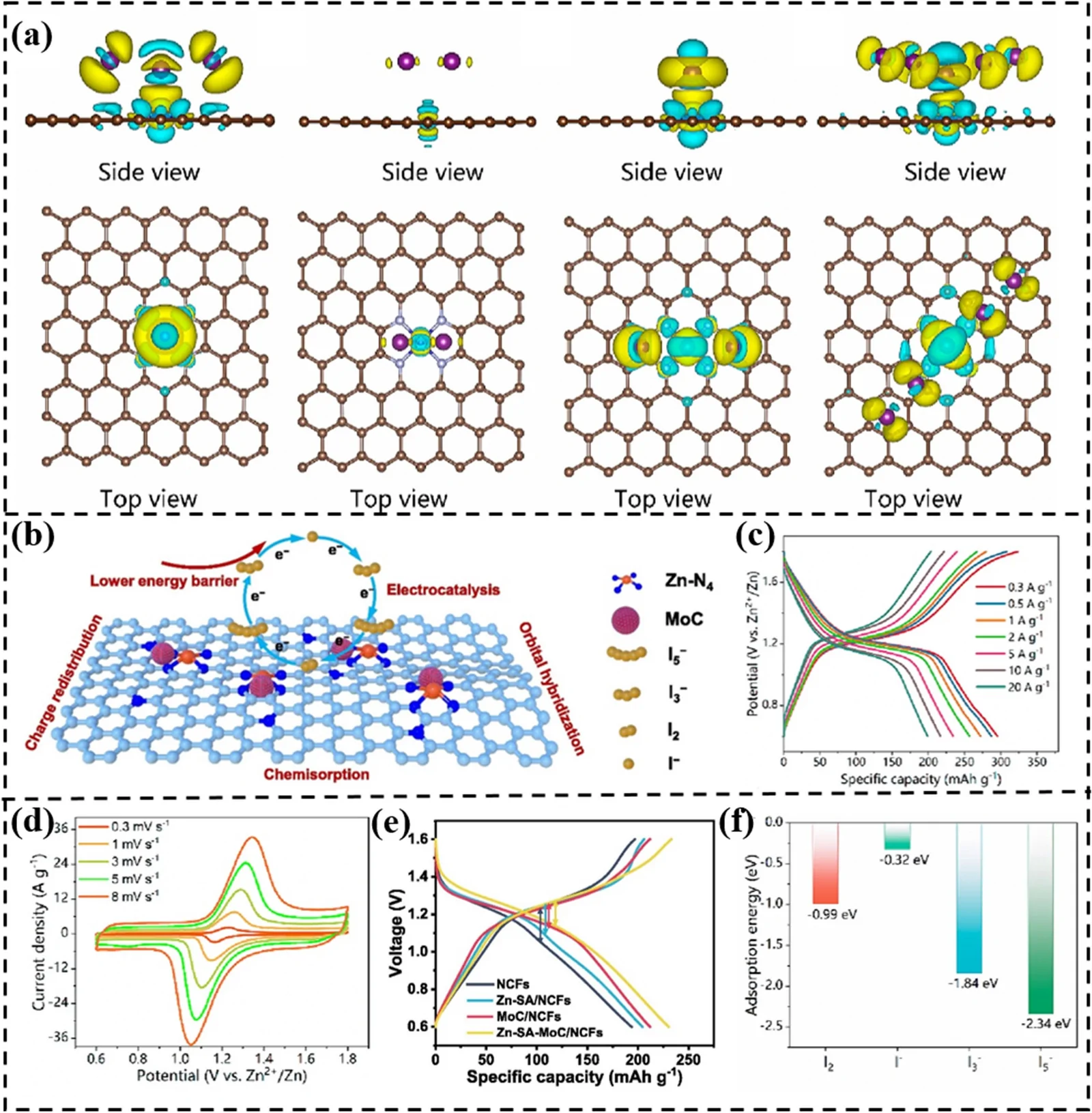
**a** Charge density difference diagram of I_2_, I^−^, I_3_^−^, and I_5_^−^ on Co site [74]. Copyright 2024, Elsevier. **b** Conceptual diagram of iodine redox reaction on Zn-SA-MoC/NCFs [122]. Copyright 2025, Elsevier. **c** Charge–discharge and d CV curve of Co-SAs@NPC/I_2_ [74]. Copyright 2024, Elsevier. **e** Charge–discharge curve of Zn-SA-MoC/NCFs [122]. Copyright 2025, Elsevier. **f** Adsorption energy of iodine species on Co site [74]. Copyright 2024, Elsevier

Cobalt-based SACs on N-doped carbon (Co-SACs@NPC), synthesized via molten salt pyrolysis, exhibited a crumpled 2D nanosheet morphology. Co-SACs@NPC were also coordinated through Co-N_4_ sites and exhibited a high specific surface area (1741 cm^2^ g^−1^), micropores (0.6–0.9 nm), and mesopores (2.2–3.8 nm). DFT results showed spontaneous adsorption and charge transfer between the Co sites and iodine species, as evident from the charge density difference diagram and adsorption energies of iodine species on Co sites in Fig. 15a,f, respectively. Co-SACs@NPC/I_2_ showed good catalytic activity and a low polarization voltage, promoting faster conversion and reversibility. The cathode also showed a high specific capacity of 295 mAh g^−1^, an excellent rate performance of 199 mAh g^−1^ at a current density of 20 A g^−1^, and long-term cycling stability over 10,000 cycles with a capacity retention of 86% and capacity decay of 0.0014%. The GCD curve of Co-SACs@NPC/I_2_ is shown in Fig. 15c, while Fig. 15d shows its CV curve [74]. Chen et al. prepared Zn SACs and molybdenum carbide nanoclusters embedded in nitrogen-doped carbon nanofibers (Zn-SA-MoC/NCFs). The resulting host exhibited a physicochemical confinement effect on the iodine species and enhanced the electron/ion transport, enabling faster iodine conversion without the shuttle effect. A schematic illustration of the iodine redox reaction on Zn-SA-MoC/NCFs is shown in Fig. 15b. DFT studies revealed that Zn SAs tune the Mo d-orbital and enhance the d–p-orbital hybridization between Mo and iodine, which enhances the adsorption and catalytic activity of MoC sites by reducing the energy barrier for iodine redox. As a result, Zn-SA-MoC/NCFs hybrid showed good performance with 230.6 mAh g^−1^ at 0.5 C (Fig. 15e) and 155.8 6 mAh g^−1^ at 10 C. It also exhibited a relatively good capacity retention of 90% over 20,000 cycles [122].

**Fig. 16:**
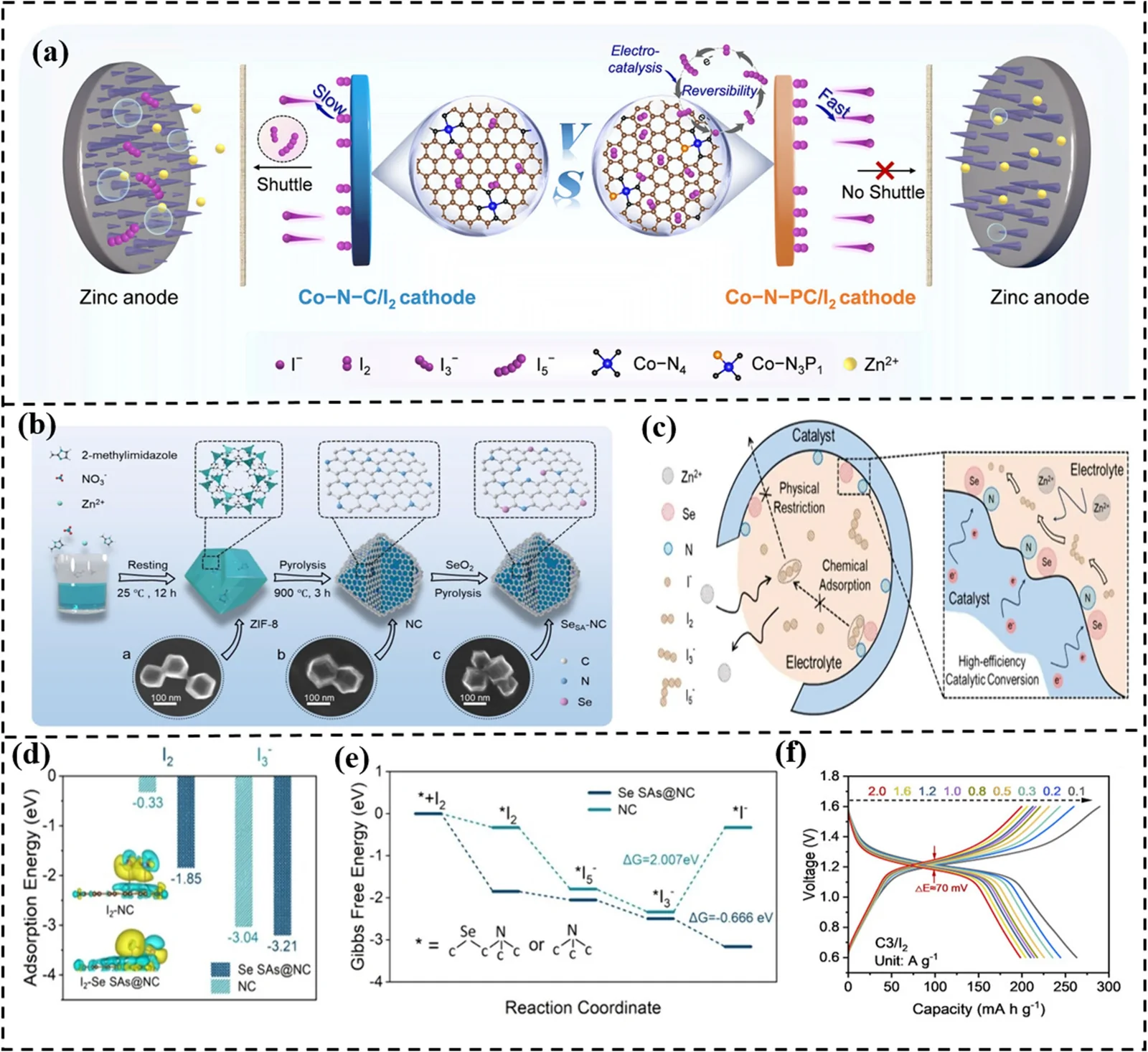
**a** Schematic comparison between Co-NC/I_2_ and Co–N-PC/I_2_ [124]. Copyright 2025, Wiley–VCH. **b** Schematic diagram of synthesis SeSA-NC-900 [51], **c** conceptual illustration for polyiodide adsorption and conversion on SeSA-NC-900 [51], **d** adsorption energy of iodine species on Se SAs@NC and NC [51], **e** Gibbs free energy diagram depicting the reduction reaction on Se SAs@NC and NC sites [51]. Copyright 2025, American Chemical Society. **f** GCD curve of Co–N-PC/I_2_ at various current densities [124]. Copyright 2025, Wiley–VCH

Semimetallic SACs can also act as robust active centers. For instance, Liu et al. used semimetallic selenium Se-SA instead of a transition metal because of its high polarizability and nucleophilicity, inducing spin/charge asymmetry. Se SAs dispersed on N-doped carbon (Se_SA_-NC) derived from ZIF-8 were reported, in which Se was coordinated in the form of C-Se-C. The larger atomic radius and lone pair of electrons on Se introduce a strong dipole and electron redistribution in the host matrix. This not only provides a superior chemical confinement of the iodine species but also enhances the redox kinetics by reducing the Gibbs free energy barrier for the iodine redox reaction. A schematic diagram of the synthesis of Se_SA_-NC from ZIF-8 and its SEM images are shown in Fig. 16b, while Fig. 16c shows the catalytic mechanism of the iodine redox reaction on Se_SA_-NC. The highly reversible binding of iodine species on Se_SA_-NC compared to NC is shown in Fig. 16d, which enables a low Gibbs free energy barrier for iodine conversion on Se_SA_-NC (Fig. 16e). Additionally, the mesoporous structure of the carbon matrix physically confines the iodine species and contributes to enhanced electronic/ionic conductivity. I_2_@Se_SA_-NC-900 cathode showed 216 mAh g^−1^ at 0.2 A g^−1^ and 182 mAh g^−1^ at 4 A g^−1^, indicative of its good rate performance. It also exhibited a relatively high capacity retention of 92% after prolonged cycling over 10,000 cycles at 1 A g^−1^ [51]. Recent work has demonstrated the superiority of asymmetric atomic sites over conventional symmetric configurations. Guo et al. reported asymmetric Co single-atom catalysts featuring Co-N_3_P_1_ coordination on a N, P-codoped carbon matrix, which showed superior performance as iodine hosts. The asymmetric coordination optimizes the electronic structure, enhancing polyiodide adsorption and reducing the energy barrier for iodine redox reactions (Fig. 16a). The Co–N-PC catalyst exhibited exceptional cyclability, retaining a specific capacity of 100.6 mAh g^−1^ after 50,000 cycles at 5 A g^−1^. GCD of optimized Co–N-PC (C3/I_2_) is shown in Fig. 16f [124]. This highlights asymmetric coordination engineering as a pivotal strategy for advancing Zn-I_2_ batteries. In conclusion, the synergistic effect of the mesoporous structure, N-doping, and atomically dispersed SACs sites effectively inhibited the shuttle effect, catalyzed redox conversion, and provided enhanced conductivity.

In Zn-halogen batteries, MOF-derived SACs are proved to be promising catalysts because of their dual benefits: They exhibit strong affinity toward polyhalide species, suppressing the shuttle effect while catalyzing redox conversion by lowering the activation energy barrier and improving reversibility.

MOF-derived systems have shown considerable performance in two-electron Zn-I_2_ batteries. However, the ultimate frontier in Zn-I_2_ batteries lies in transcending the conventional two-electron (I_2_/I^−^) redox to access the high capacity four-electron (I^−^/I^0^/I^+^) redox pathway. While traditional transition metal compounds have demonstrated significant catalytic effects in Zn-I_2_, their role is often confined to enhancing two-electron kinetics and they face inherent limitations in stabilizing the complex intermediates of the I^−^/I^0^/I^+^ pathway due to their heterogeneous active sites [125]. In contrast, MOFs and their derivatives have emerged as uniquely powerful platforms. The inherent porous framework of MOFs provides an intrinsic “confinement–catalysis” where the well-defined pores physically trap iodine intermediates, adjacent to atomic-scale catalytic sites, creating an efficient host design for a four-electron transfer redox pathway. For instance, UiO-66-NH_2_ nanoemulsion-derived Fe SAC-MNC showed potential for four-electron transfer Zn-I_2_ batteries (Fig. 14c) besides the two-electron process. It achieved 230 mAh g^−1^ at 0.5 A g^−1^ and 109.49 mAh g^−1^ at 10 A g^−1^ in a four-electron transfer Zn-I_2_ battery [105]. MOFs can also be molecularly engineered to cooperate with advanced electrolytes, enabling full four-electron systems. A recent study revealed a more holistic strategy of electrode–electrolyte synergy to enable a four-electron redox pathway. The system employs a 1-methyl-3-propylimidazolium iodide (MPII)/Zn(SO_3_CF_3_)_2_ electrolyte where MPII interacts with SO_3_CF_3_^−^ to generate I^+^, which enables a four-electron redox pathway. In-MOF simultaneously coordinate these I^+^ species through –C=O and C–OH groups and alloy with Zn^2+^ at In centers. Ex situ FTIR is used to validate I^+^ storage sites in In-MOF. This synergistic design delivers a very high capacity of 481 mAh g^−1^ at 1 A g^−1^ [126]. In summary, MOFs move beyond the catalytic role of transition metal compounds by offering a designable host environment that integrates confinement, catalysis, and electrolyte interaction. This makes them essential materials for realizing stable, high capacity four-electron Zn-I_2_ batteries.

#### Other MOF-Based Hosts

**Fig. 17:**
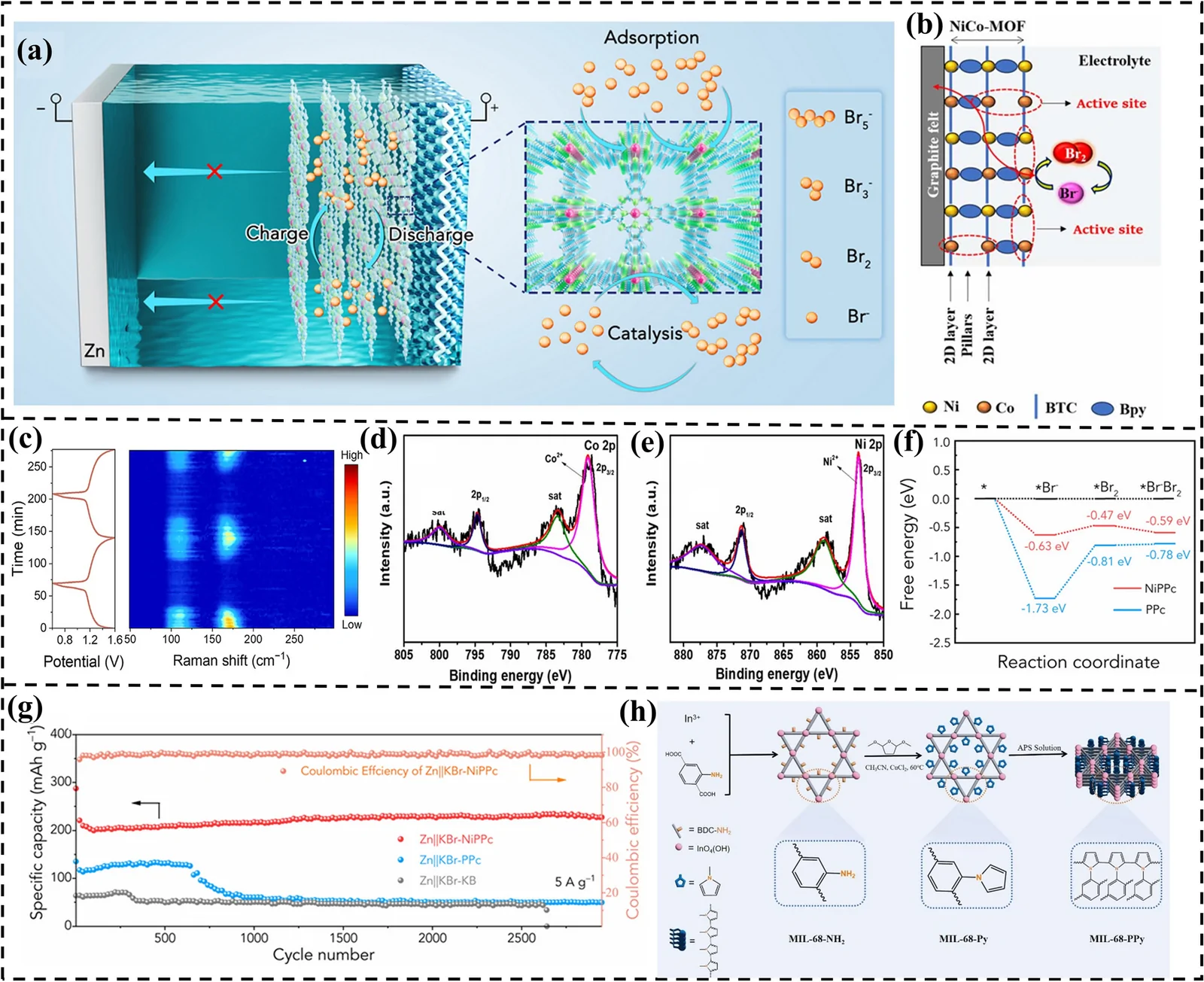
**a** Schematic illustration and working mechanism of NiPPc host [50]. Copyright 2023, Royal Society of Chemistry, **b** NiCo-MOF@GF [48]. Copyright 2024, Royal Society of Chemistry, **c** in situ Raman spectra of the I_2_@Al-TCPP(Fe) cathode at different discharge/charge states [93]. Copyright 2025, Wiley–VCH. **d**, **e** XPS spectra of Co and Ni in NiCo-MOF@GF [48]. Copyright 2024, Royal Society of Chemistry. **f** Gibbs free energy of Br redox reaction on NiPPc and NPc sites [50], **g** cycling stability test of Zn||KBr-NiPPc [50]. Copyright 2023, Royal Society of Chemistry. **h** Schematic diagram representing the synthesis of MIL-688-PPy [59]. Copyright 2025, Elsevier

Although most studies on Zn-halogen batteries have focused on MOF-derived carbon hosts, some recent advancements have highlighted the potential of conductive or compositely engineered MOF-based cathodes because of their conductivity, contributing to better performance. A transformative advancement in this field is the development of strategically engineered pristine MOFs as direct cathode hosts, bypassing pyrolysis to leverage their intrinsic crystalline order and well-defined catalytic sites. A notable example in this field is the work on Al-TCPP(Fe) MOF. This material exemplifies the synergistic integration of multiple design principles: Its well-ordered pore structure (2.4 nm) provides spatial confinement to physically trap polyiodides while the atomically dispersed Fe-N_4_ sites act as highly effective catalytic centers. The Fe-N_4_ moieties, benefitting from quantum size effect, enhance charge transfer and significantly lower the energy barrier for I_2_/I^−^ redox reaction, thereby accelerating the kinetics, as confirmed by DFT calculations. In situ Raman spectroscopy in 2 M ZnSO_4_ revealed consistently weak signals for polyiodides throughout cycling, highlighting their effective confinement (Fig. 17c). The Zn-I_2_ battery assembled with Al-TCPP(Fe) MOF as cathode exhibited a highly reversible capacity of 210.94 mAh g^−1^ at 1 C and achieves an ultralong lifespan of 54,000 cycles [93]. Two-dimensional (2D) conductive MOFs are another emerging class of materials that can improve the performance of Zn-halogen batteries [127]. Wei et al. reported porous 2D conjugated nickel polyphthalocyanine (NiPPc) MOF, which served as a multifunctional host in Zn-Br_2_ static battery. NiPPc offers unique adsorption–catalysis synergy containing atomically dispersed Ni-N_4_ catalytic sites to simultaneously catalyze redox conversion and immobilize polybromide species, as shown in Fig. 17a. The EXAFS fitting curve of NiPPc confirms the presence of Ni-N_4_ atomic sites, which effectively confined polybromide species and catalyzed bromine conversion. DFT studies also showed a lower Gibbs free energy barrier for the bromine redox reaction on NiPPc than on PPc, as shown in Fig. 17f. The π–π stacking in phthalocyanine improved charge transfer, contributing to faster kinetics, while the intrinsic porosity contributed to high bromine loading. Consequently, the NiPPc-based cathode exhibited a high capacity of 265 mAh g^−1^ at 2 A g^−1^ and retained 240 mAh g^−1^, 95% of its initial capacity, after 3,000 cycles at 5 A g^−1^ (Fig. 17g) [50].

Bimetallic MOFs also showed good performance owing to the presence of metallic active sites. Bimetallic NiCo-MOF-modified graphite felt (NiCo-MOF@GF) was developed by the hydrothermal method and mild thermal treatment (400 °C), serving as a cathode in a Zn-Br_2_ redox flow battery. NiCo-MOF@GF showed 2D nanosheet morphology and contained unsaturated Ni^2+^ or Co^2+^ coordination sites and enhanced electrochemically active surface area. High-resolution XPS spectra of Co and Ni are shown in Fig. 17d, e, respectively, which confirmed the presence of unsaturated Co^2+^ and Ni^2+^ sites. The unsaturated metal centers acted as robust active sites, and the electron coupling effect between Ni and Co further enhanced 2Br^−^/Br_2_ redox reaction. The as-prepared host also showed efficient charge transfer, which was confirmed by its low charge transfer resistance and overpotential, leading to faster redox kinetics. NiCo-MOF@GF maintained CE of 97.1%, VE of 81.1%, and EE of 78.5% at 40 mA cm^−2^ for about 200 cycles [48].

Another category is the composite of In-MOF with conductive polymers such as polypyrrole (MIL-68-PPy), which was formed by transforming the –NH_2_ groups of In-MOF (MIL-68-NH_2_) to pyrrole rings, which were subsequently polymerized by oxidative polymerization to form a covalently interlocked polypyrrole conductive network within the MOF. Oxidative polymerization using ammonium persulfate (APS) erodes the MOF surface from the inside out, generating abundant pores and oriented cracks. A schematic of the synthesis of MIL-68-PPy is shown in Fig. 17h. The conductive network enhances charge transport by providing new electron transport pathways, whereas mesoporosity facilitates iodine immobilization, inhibiting the shuttle effect. The conjugated π–π bonds in polypyrrole chains on the I_2_@MIL-68-PPy electrode facilitated reversible redox reactions. I_2_@MIL-68-PPy exhibited high specific capacities of 209 and 115 mAh g^−1^ after 1000 cycles [59].

In summary, the evolution of MOF-based cathodes for Zn-halogen batteries demonstrates a clear paradigm from simple porous confinements to strategically engineered single-atom catalytic sites. While pore engineering and heteroatom doping provide a foundational strategy for physical and chemical confinement, the most remarkable performance breakthroughs have been achieved through advanced carbon architectures engineering and SACs. The development of hierarchical structures like hollow carbon nanostraws, carbon nanofibers, and defect-engineered frameworks addresses multiple challenges simultaneously: providing abundant confinement space, ensuring efficient mass/electron transport, and offering mechanical stability during cycling. The emergence of SACs represents the most vital strategy where atomically dispersed sites in N-doped matrix provided ideal “confinement–catalysis” synergy. This is the most promising strategy in carbon-based MOFs, not only for optimizing conventional two-electron transfer, but also crucial for unlocking the high-capacity four-electron pathway. Notably, the recent success of pristine MOFs like Al-TCPP(Fe) demonstrates that molecular-level design in the crystalline state can achieve superior “confinement–catalysis” without the need for carbonization, opening a new avenue for host material design. This system has demonstrated excellent performance metrics including capacities exceeding 200 mAh g^−1^, exceptional rate capability, and extraordinary cycling stability.

Tables 2, 3 provide a systematic comparative analysis of the performance of the MOF-based cathodes used in Zn-I_2_ and Zn-Br_2_ batteries, respectively, along with their design strategies and electrochemical performances.

**Table 2:** MOF-derived cathodes used in Zn-I_2_ batteries, their design strategy, role, and performance

SrNo	MOF Source	Material Derived	Pores / SSA	Engineering Strategy	Key functional role	Capacity	Cycle Life	Reference
*Pore-engineered carbon*								
1	MOF-5	MPC/I₂	Mesoporous	Separator + porous cathode synergy	Confinement in pores	137 mAh g^−1^@0.1 A g^−1^, 112 mAh g^−1^@1 A g^−1^	300 cycles	[116]
2	Zn-MOF-74	I_2_@P2-1000	4.45 nm	Pyrolysis @1000 °C	Size confinement strategy	179.9 mAh g^−1^@100 mA g^−1^	100 cycles, 79% capacity retention	[96]
*Heteroatom doped carbon*								
3	ZIF-8	N, P co-doped carbon NPPC	Hierarchical porosity	(NH_4_)_3_PO_4_-assisted pyrolysis	Strong chemisorption of iodine at heteroatomic sites	175 mAh g^−1^@0.1 A g^−1^	6,000 cycles	[101]
4	ZIF-8	I_2_@S3-1000	3.3 nm; 538.2 m^2^ g^−1^	CTAB-regulated synthesis + N-doping	N-moieties enhances chemisorption + kinetics	112.4 mAh g^−1^@2 A g^−1^	10,000 cycles	[45]
5	ZIF-8	Carbon morphologies (CDs, CNSs, CNSSs, CSs)	Tunable pore size	Additive-assisted carbonization + doping	Heteroatomic species enhance chemisorption	313.6 mAh g^−1^@0.5 C	6,000 cycles@20 C	[102]
6	ZIF-67	Co/N co-doped porous carbon	High SSA	In situ carbonization	Strong I^−^ binding + redox kinetics	152.4 mAh g^−1^@5 C	24,000 cycles, 80% capcity retention	[118]
7	PTA@ZIF-8	W₂N/N-doped porous carbon	Hierarchical porous	Self-nitridation & carbonization	W-based catalytic & adsorptive support	200.9 mAh g^−1^@5 C	2,000 cycles	[119]
*Advanced carbon architectures*								
9	InOF-1	HCNS/I_0.5_	0.66 nm, 1.25 nm (micro); 2.71 nm, 9.98 nm(meso)	Carbonization + Ir fusion	I_2_ confinement + ion pathway	234.1 mAh g^−1^@1 A g^−1^	1, 500 cycles, 87% CE	[110]
10	Al-MOF	I_2_@NPCNFs-800	Microporous; 468 m^2^ g^−1^	Electrospinning + N-doping	Cathode host + ion/e⁻ transport	138.9 mAh g^−1^@10C	6,000 cycles	[99]
11	Ni/Zn ZIF-8	NZ-aNC	–	Ni/Zn co-doping, pre-activation	Defects enhanced I_2_ chemisorption + catalysis	219 mAh g^−1^@5 A g^−1^	20,000 cycles	[98]
*SACs*								
12	ZIF-8	Zn-SA-MoC/NCFs	5.7 nm	Zn SAs + Mo synergistic effect	Efficient catalysis + I_2_ trap at single-atom sites	230.6 mAh g^−1^@0.5 A g^−1^	20,000 cycles, 90% capacity retention	[122]
13	ZIF-8	I_2_@SeSA-NC-1100	Micro + Mesoporous	Semimetallic SACs	Polyiodide catalysis + adsorption	216 mAh g^−1^@0.2 A g^−1^	10,000 cycles, 92% CE	[51]
14	ZIF-8	Fe–N–C	2.82 nm	Fe SAC doping + pyrolysis	Redox catalysis + I_2_ chemisorption	214.9 mAh g^−1^@0.1 A g^−1^; 161.9@2 A g^−1^	10,000 cycles	[57]
15	UiO-66-NH_2_	Fe SAC-MNC	Mesopores	Fe SAC + nitrogen doping	SAC catalysis + confinement	188.2 mAh g^−1^@0.3 A g^−1^	50,000 cycles	
16	ZIF-8	Co-SAs@N-doped carbon	SSA: 1741 m^2^ g^−1^	Molten salt pyrolysis + Co SAC + 2D nanosheet morphology	SAC catalysis + I^−^ adsorption + enhanced ion transport	295 mAh g^−1^@0.3 A g^−1^	10,000 cycles	[74]
17	ZIF-8	Ni SAC in N-doped carbon	–	Ni SAC + activation pyrolysis	Reduced shuttle effect + superior adsorption energies	193 mAh g^−1^@6 A g^−1^	10,000 cycles	[123]
18	PPh_3_@Co-ZIF-8	Co–N-PC	2.4–2.6 nm	Asymmetric Co-N_3_P_1_ SACs	Asymmetric SACs enhance confinement + catalysis	207.9 mAh g^−1^@0.8 A g^−1^	50,000 cycles	[124]
*Other MOF-based hosts*								
19	MIL-68-NH_2_	I_2_@MIL-68-PPy	Large mesopores	Composite formation	Enhanced charge transfer	209 mAh g^−1^@0.5 A g^−1^	1000 cycles	[59]
20	Al-TPCC(Fe)	I_2_@Al-TCPP(Fe)	2.4 nm	Quantum size effect synergizes space limited domain	Superior redox catalysis + confinement at atomic sites due to quantum size confinement effect	210.95 mAh g^−1^ at 1 C	54,000 cycles	[93]

**Table 3:** MOF-derived cathodes used in Zn-Br_2_ batteries, their design strategy, role, and performance

Sr No	MOF Source	Material Derived	Pore Size / SSA	Engineering Strategy	Key Functional Role	Performance Summary	References
*MOF-derived porous carbon*							
1	Nanozeolite-type ZIF (NSZIF)	PNSC	2–10 nm	Pyrolysis + CO_2_ activation	Br₂/Br⁻ redox active host	VE 83%, EE 82% @80 mA cm^−2^, 200 cycles	[100]
2	ZIF-8	CZGF-1000	Micro and mesopores	In situ growth + carbonization @1000 °C	Br⁻/Zn^2^⁺ regulation and dendrite suppression	EE 90% @50 mA cm^−2^, EE 68% @250 mA cm^−2^, > 2000 cycles	[52]
3	ZIF-8	N-doped carbon felt	–	ZIF-8 in situ growth on graphite felt	Anchors Br⁻, enhances conductivity	900 cycles @100 mA cm^−2^	[66]
4	ZIF-8	N-doped graphite felt (NGF)	–	Electrochemical + chemical growth	Bipolar electrode for Zn and Br redox	1.8 V discharge, 88.1% VE @100 mA cm^−2^	[128]
5	Fe/Zn-ZIF	Fe–N-CNFs	2.5 nm	Electrospinning + carbonization	Redox sites + ion diffusion enhancer	EE 81%, VE 82.4%, CE 98.4% @80 mA cm^−2^	[111]
*Other MOF-based hosts*							
6	Ni/Co MOF	Ni/Co MOF@GF	–	Bimetallic MOF	Br₂ redox catalytic site	CE 97.1%, EE 78.5%, VE 81.1% @40 mA cm^−2^	[48]
7	NiPPc	Ni–N₄ polyphthalocyanine MOF	–	Ni-N_4_ sites	Br⁻/Br₃⁻ catalysis + adsorption	265 mAh g^−1^@2 A g^−1^, 240 mAh g^−1^@5 A g^−1^, 1.82 V plateau, 3000 cycles	[50]

### Practical Demonstration of MOF-Based Cathodes

**Fig. 18:**
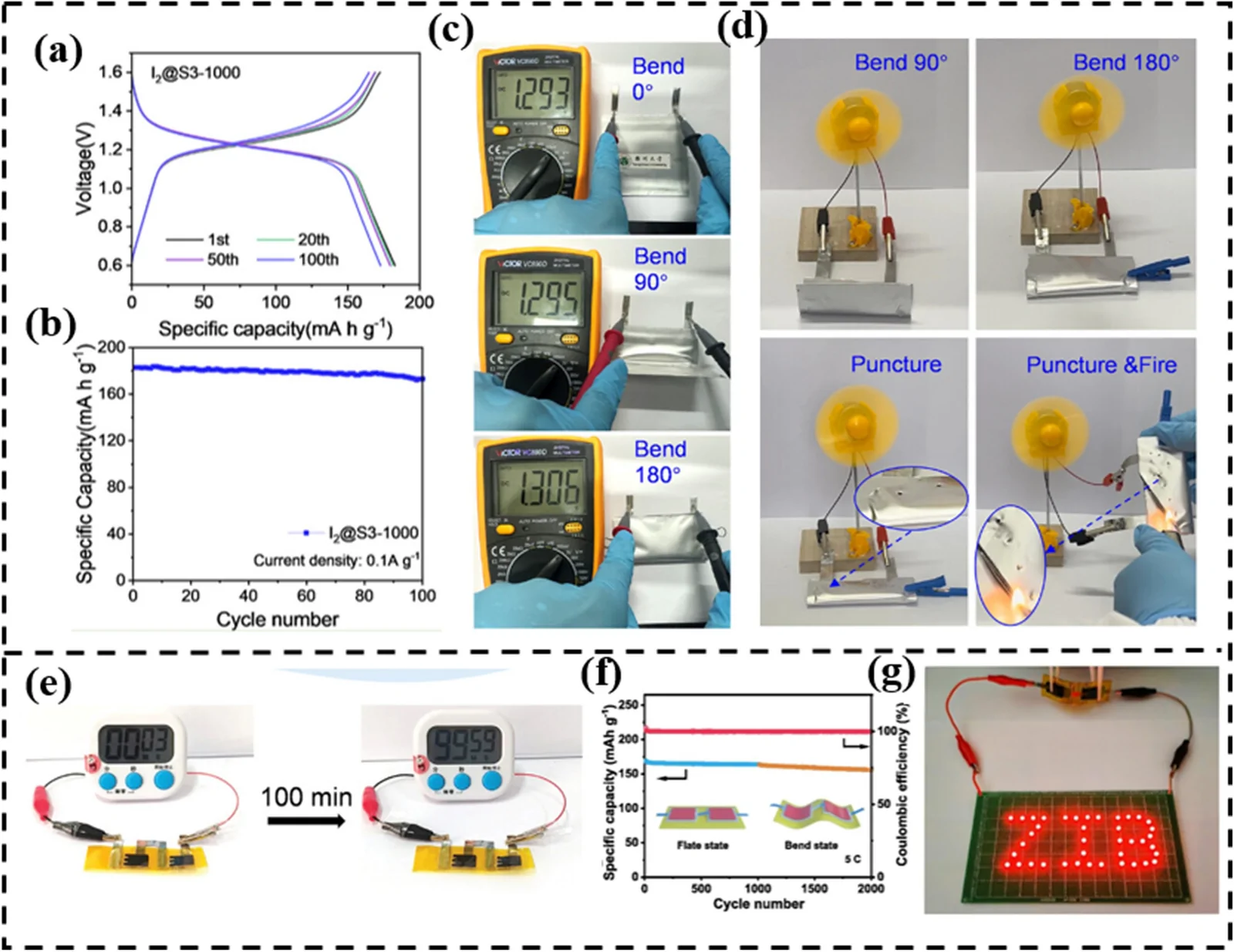
**a** GCD curve and **b** cycling stability test of the soft pack battery based on I_2_@S3-1000 cathode [45], **c, d** voltage stability of a single soft pack battery and driving a small electric fan powered by a soft pack battery at various conditions [45]. Copyright 2025, Wiley–VCH. **e** Image showing the operation of a timer (up to 100 min) powered by two microdevices [96]. Copyright 2024, Wiley–VCH. **f** Stability test of quasi solid-state Zn-SA-MoC/NCFs battery under flat and bending status [122]. **g** Photograph showing two batteries in series lightning 40 LEDs [122]. Copyright 2025, Elsevier

In addition to laboratory testing, several MOF-based cathodes have shown remarkable promise in practical applications such as flexible and wearable Zn-halogen batteries. For example, Fe–N-C derived from ZIF-8 has been used to fabricate a flexible soft pack and a microscale Zn-I_2_ battery, capable of powering a timer, LEDs, and small fan under bending conditions [57]. Zn-MOF-derived porous N-doped carbon cathode was also used to assemble a soft battery pack, and it delivered discharge/charge capacities of 182.8/172.4 mAh g^−1^ at 0.1 A g^−1^ and 172.9 mAh g^−1^ after 100 cycles, as represented in its charge/discharge curve (Fig. 18a) and cyclability test (Fig. 18b), respectively. The soft battery pack bent at various angles from 0 to 180° delivered a stable voltage of 1.29 V and continuously powered a small electric fan under bending, puncture, and fire conditions, as shown in Fig. 18c, d [45]. CNSs/I_2_-based Zn-I_2_ battery, charged at 3 C, continuously powered an NMU-shaped lamp comprising 21 LEDs for over 3 min [102]. Three soft pack batteries connected in series using Zn-MOF-74-derived porous carbon nanorods cathodes were used to charge an Android phone. A flexible quasi-solid-state battery assembled using the same cathode has powered an electronic timer for over 100 min under bending conditions, as shown in Fig. 18e [96]. A quasi-solid-state Zn-I_2_ battery fabricated using a fiber-based Zn-SA-MoC/NCFs cathode retained 92% of its capacity over 2,000 cycles under both flat and bent conditions, and two cells connected in series successfully powered 40 LEDs, as shown in Fig. 18f, g [122]. This demonstrates the real-world applications of MOF-based cathodes in Zn-halogen batteries.

## MOFs for Zn Anode Protection in Zn-Halogen Batteries

Despite the critical role of the cathode in Zn-halogen batteries, the performance and longevity of these systems are strongly influenced by the stability of the Zn anode [136]. Dendrite growth, side reactions, and corrosion, particularly in polyhalide-rich electrolytes, pose significant challenges to the reversibility and safety of Zn plating/stripping [136–138]. Therefore, engineering the anode surface either by designing a structured host or by applying coatings has become a key strategy for regulating Zn deposition behavior, suppressing side reactions, and enhancing cycling stability [139–141]. MOFs have emerged as promising materials to address these issues by acting as both a host and a protective coating for Zn anodes [53,142]. The summary of the MOF-derived anode hosts and coatings used in Zn-halogen batteries is provided in Table 4.

**Table 4:** MOF-derived anodes used in Zn-halogen batteries, their design strategy, role, and performance

Sr No	Material	System	Key functional role	Zn deposition behavior	CE	EE	Cycling	References
1	ZIF-8–500	Static Zn-I_2_	MOF-based host	Smooth, dendrite-free Zn, suppress HER	99.8%	197.9 Wh kg^−1^ at 215.6 W kg^−1^	97% retention over 1600 cycles	[129]
2	CZ-5	Zn-Br_2_ redox flow battery	MOF-based defective carbon host	Smooth, dense plating	97%@120 mA/cm^2^	67%@120 mA cm^−2^	5000 cycles @100 mA cm^−2^	[130]
3	CZGF-1000	Zn-Br_2_ redox flow battery	MOF-based N-doped carbon host	Dendrite-free deposition	99.2%	68%	800 cycles @250 mA cm^−2^	[52]
4	3D ZIF-8@MXene composite	Static Zn-I_2_	MOF-based composite coating	Dendrite-free deposition, suppress HER and corrosion	99.93%	–	86.1% retention over 2400 cycles	[53]

**Fig. 19:**
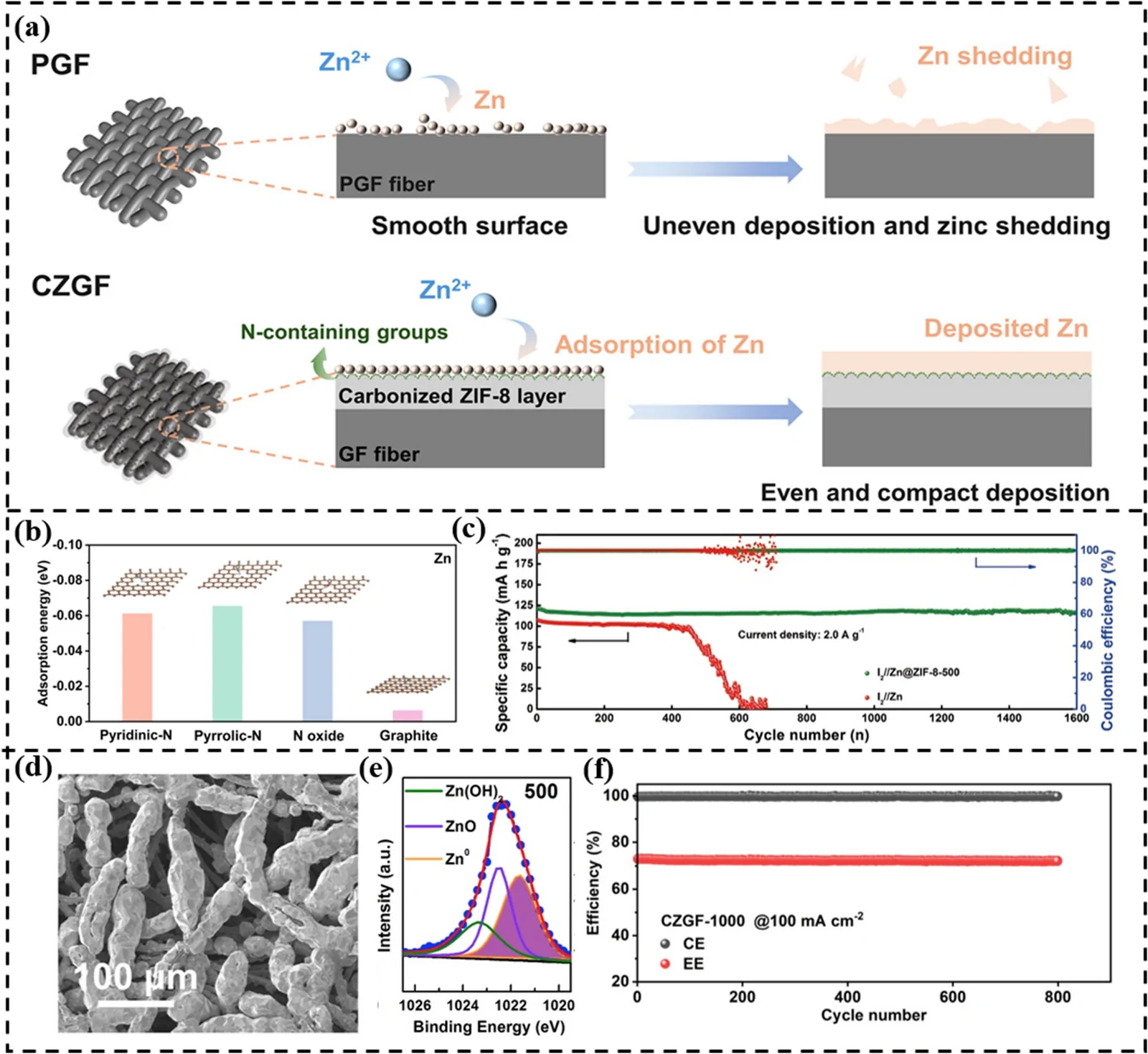
**a** Schematic diagram of uniform Zn deposition over CZGF, **b** adsorption energy of Zn over different N-sites [49]. Copyright 2025, American Chemical Society, **c** cycle performance of I_2_//Zn@ZIF-8–500 [129]. Copyright 2019, Elsevier. **d** SEM image of Zn deposition on CZGF-1000 after 12 min of charging at 100 mA cm^−2^ [49]. Copyright 2025, American Chemical Society. **e** XPS spectra of ZIF-8–500 [129]. Copyright 2019, Elsevier. **f** Cycling performance of CZGF-1000 [49]. Copyright 2025, American Chemical Society

In the host strategy, MOFs provide a confined environment to guide uniform Zn deposition. Wang et al. used ZIF-8 thermally treated at 500 °C (ZIF-8–500), which preserved micropores and contained trace metallic Zn^0^, as confirmed by high-resolution XPS spectra (Fig. 19e). These Zn^0^ sites acted as uniform nucleation centers, enabling dendrite-free Zn deposition with high reversibility (CE up to 99.8%). The ZIF-8–500 also suppressed the parasitic HER due to its high overpotential. A full cell, aqueous rechargeable I_2_//Zn@ZIF-8–500, demonstrated excellent performance with 97% capacity retention and 100% CE after 1,600 cycles, as shown in the cycling stability test in Fig. 19c. The anode exhibited a dense, smooth, and dendrite-free surface after cycling [129]. N-containing functionalities can provide more zincophilic sites, while enhancing Zn^2+^ adsorption and promoting uniform nucleation [143]. For instance, Li et al. prepared an anode by the in situ growth of ZIF-8 on graphite felt, followed by carbonization (CZGF). The carbonized ZIF-8 layer introduced N-containing functional groups, such as pyridinic N, pyrrolic N, graphitic N, and oxidized N, which significantly enhanced Zn adsorption through lone pair coordination and improved the uniformity of Zn nucleation. DFT calculations confirmed that these functional groups exhibited more negative Zn adsorption energy than pristine graphite, as shown in Fig. 19b. Additionally, the lone pairs of N enhance the uniform distribution of the electric field, reducing localized current, which may promote irregular Zn growth. Moreover, phase-field simulations revealed that the rough CZGF surface with engineered microprotrusions promoted uniform deposition and enhanced Zn^2+^ transfer at the interface. A schematic diagram of the uniform Zn deposition over the CZGF is shown in Fig. 19a. As a result, the optimized electrode CZGF-1000 exhibited exceptional cycling stability with no performance decay over 800 cycles at 100 mA cm^−2^, maintaining a CE of 99.2%, as shown in the cycling stability test in Fig. 19f. CZGF-1000 also shows a uniform dense morphology after cycling, as shown in the SEM image in Fig. 19d. The Zn-Br_2_ redox flow battery assembled using CZGF on both the positive and negative electrodes exhibited an EE of 68% at a high current density of 250 mAh cm^−2^ [52]. N doping is effective for promoting uniform Zn deposition, but it may catalyze parasitic HER reactions [129].

**Fig. 20:**
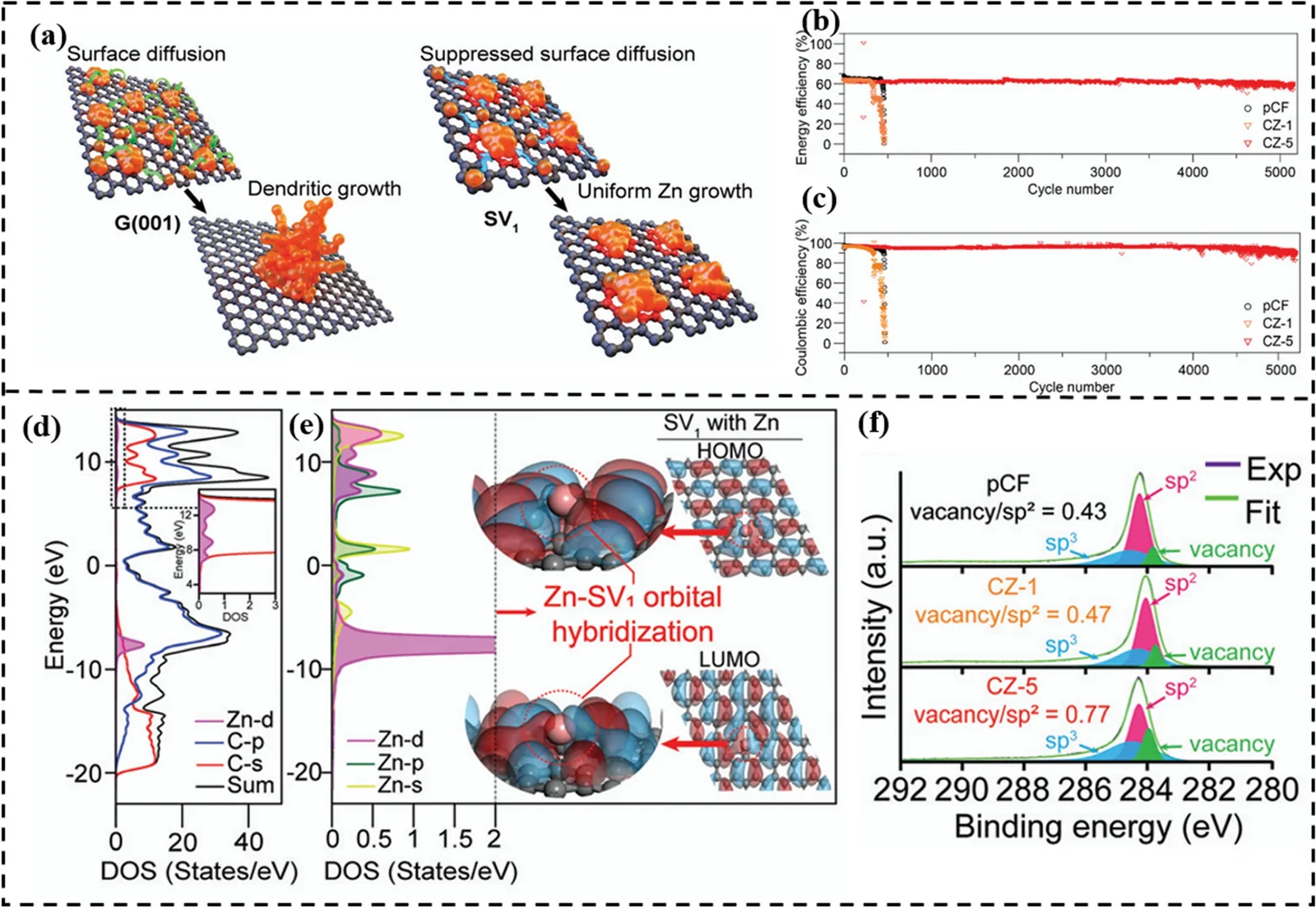
**a** Schematic representation of Zn growth on graphene surface and carbon containing single-vacancy defects [130]. **b** CE and **c** EE of CZ-5 [130]. **d** DFT calculations of DOS and **e** pDOS of orbital hybridization between Zn and single-vacancy defects. **f** XPS spectra of CZ-5 [130]. Copyright 2020, Royal Society of Chemistry

In more advanced host designs, MOF-derived defective carbon materials exhibit superior performance. ZIF-8-derived carbon, carbonized at optimized conditions (CZ-5 at 1000 °C for 5 h), developed single-vacancy defects that enhanced Zn adsorption and suppressed lateral surface diffusion, as represented by the schematic diagram in Fig. 20a. DFT simulations revealed strong orbital hybridization between Zn and the dangling bonds of the defects, which promoted uniform plating and eliminated dendritic growth. Density of states (DOS) and partial density of states (pDOS) calculations showing orbital hybridization between Zn and single-vacancy defects is shown in Fig. 20d, e, respectively, whereas Fig. 20f shows the XPS spectra of CZ-5. A stable cycling performance of 5,000 cycles was achieved at 100 mA cm^−2^ for CZ-5 while maintaining a CE up to 97% in a Zn-Br_2_ redox flow battery. EE and CE plots of CZ-5, CZ-1, and pCF over prolonged cycling are shown in Fig. 20b, c, respectively [130].

**Fig. 21:**
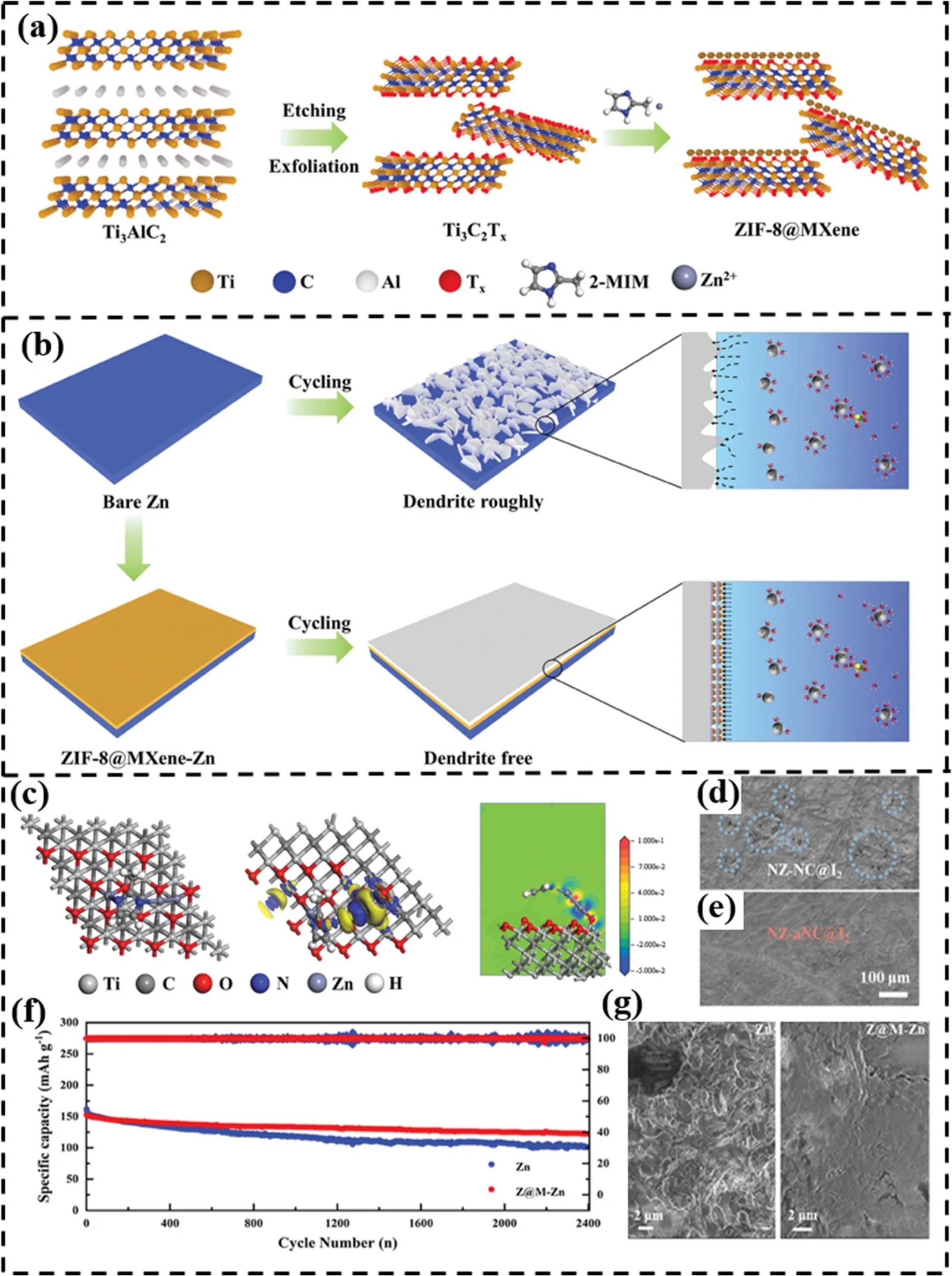
**a** Schematic synthesis of Z@M and **b** uniform Zn plating over Z@M [53], **c** geometrical optimization model and differential charge density of Zn adsorbed on Z@M [53]. Copyright 2025, Wiley–VCH. **d**, **e** SEM images of uniform Zn plating using NZ-aNC@I_2_ as cathode [98]. Copyright 2025, Wiley–VCH. **f** Cycling performance of Z@M-Zn. **g** SEM images of bare Zn and Z@M-Zn after rate test [53]. Copyright 2025, Wiley–VCH

In the coating strategy, MOFs can also function as a surface coating for the Zn anodes, where they modify the electrode–electrolyte interface. Liu et al. developed a 3D ZIF-8/MXene (Z@M) composite coating for a Zn-I_2_ battery, which combines the hydrophilic, conductive nature of MXene with the Zn^2+^ affinity of ZIF-8. This interface modification provided dual coordination sites (Ti–O and Zn-N) that enhanced Zn^2+^ directional nucleation, thereby preventing MXene restacking and effectively suppressing dendrite growth. The schematic illustration of the synthesis of Z@M is shown in Fig. 21a, whereas Fig. 21b shows the uniform Zn plating over Z@M. The hydrophilicity of Z@M also effectively decreased the contact angle with the electrolyte, reducing the undesirable HER and Zn corrosion. DFT results confirmed higher Zn adsorption energy on Z@M (2.80 vs. 0.32 eV for bare Zn). The model optimization and the differential charge density diagram of Zn adsorbed on Z@M are shown in Fig. 21c. As a result, Zn-Z@M exhibited 1050 h stable cycling at 1mA cm^−2^, and the aqueous Zn-I_2_ battery assembled using Zn-Z@M with porous carbon as the cathode exhibited a long life of 2400 cycles with a capacity retention of 86.1% (Fig. 21f). SEM images of bare Zn and Z@M-Zn after the rate capability test show a smooth surface of Z@M-Zn (Fig. 21g), indicating its uniform Zn deposition pattern as compared to bare Zn [53].

Zn anode corrosion is a critical challenge in aqueous Zn-halogen batteries, which is often exacerbated by the polyhalide shuttle effect, where active halogen species diffuse through the electrolyte and cause corrosion on the Zn surface. MOF-based halogen cathodes that effectively suppress shuttles exhibit minimized corrosion at the Zn anode. For example, NZ-aNC@I_2_ successfully suppressed the shuttle effect and no obvious corrosion was observed after 500 cycles. SEM image of uniform Zn plating using NZ-aNC@I_2_ as the cathode is shown in Fig. 21e, indicated by its smooth surface, whereas Fig. 21d shows SEM images indicating corrosion when NZ-NC@I_2_ was used as the cathode. This confirms excellent shuttle and corrosion inhibition by NZ-aNC@I_2_ [98].

Collectively, MOF-based materials offer a multifaceted approach to addressing anode limitations. These materials open new avenues for the development of high-performance dendrite-free Zn-halogen batteries with prolonged cycling and enhanced safety.

## MOFs for Separators in Zn-Halogen Batteries

The advancement of separators for Zn-halogen batteries is a key area of focus for improving Zn-halogen battery systems [144]. MOFs have gained significant attention as next-generation separator materials owing to their tunable pore structures and customizable chemical functionalities [145–147]. Unlike traditional polymer-based membranes, MOFs offer molecular design capabilities that effectively address the major issues of Zn-halogen, including the shuttle effect, dendrite formation, and corrosion. The key design principles for MOF-based separators are based on pore size engineering to allow selective ion transport [131], strategic functionalization for targeted chemical interactions [132], and incorporation of catalytic metal sites for active polyhalide utilization [52]. The performance metrics of MOF-based separators used in Zn-halogen batteries are summarized in Table 5.

**Table 5:** MOF-based separators used in Zn-halogen batteries, their design strategy, role, and performance

Sr No	Material	System	Design strategy	Key functional role	Electrochemical Performance	References
1	UD-66-PVDF-13.6	Zn-I_2_ redox flow battery	Binder-controlled restrained second growth method	UD-66 ordered pores impede active I_3_^−^ species and homogenize Zn deposition	94.5% CE #81% EE#800 cycles	[131]
2	Zn-BTC MOF membrane (Zn_3_(BTC)_2_)	Zn-I_2_ battery	Zn-BTC MOF-coated GF	Block shuttling effect and form highly aggregated contact ion pairs near Zn anode	84.6% capacity after 6000 cycles#99.65% CE	[42]
3	UiO-66-(COOH)_2_/GF	Zn-I_2_ battery	Glass fiber separator dip-coated with carboxyl-functionalized UiO-66 MOF	Carboxyl group zincophilic nature enhanced Zn^2+^ and flux while its negative charge blocked iodine shuttle	103.8 mAh g^−1^ after 35,000 cycles at 10 C	[132]
4	NF/U-AS	Zn-Br_2_ battery	Amine and sulfur functionalization of UiO-66	Suppress bromine crossover and promote uniform Zn deposition	At 1% DOC, 98% CE after 5000 cycles#At 10% DOC, 93.6% CE #At 40% DOC, 78.19% DOC	[133]
5	ZnO@GF	Zn-I_2_ battery	Polar–nonpolar synergy: nonpolar PC cathode host and polar ZnO-modified separator	PC loads I_2_, and ZnO adsorbs and catalyzes the reaction of polyiodide intermediates, preventing shuttling and enhancing reaction kinetics	209.9 mAh g^−1^ capacity at 0.1 A g^−1#^97.5 mAh g^−1^ capacity retained after 2600 cycles at 0.5 A g^−1^	[134]
6	ZnMn-NC/GF	Zn-I_2_ battery Zn-Br_2_ battery#	Entrapment–adsorption–catalysis strategy	Adsorb intermediates, Catalyze conversion, Block shuttle	Zn-I_2_ battery#30,000 cycles at 5 A g^−1^ with 95.3% capacity retention#Zn-Br_2_ battery#3,000 cycles at 5 A g^−1^	[52]
7	NH_2_-MIL-125/GF	Zn-I_2_ battery	Three-party synergistic strategy	Inhibits polyiodide shuttling, selective Zn^2+^ transport, and reduces interfacial resistance	215 mAh g^−1^ at 0.05 A g^−1#^88% capacity retention after 10,000 cycles at 2 A g^−1#^99.3% CE	[135]

**Fig. 22:**
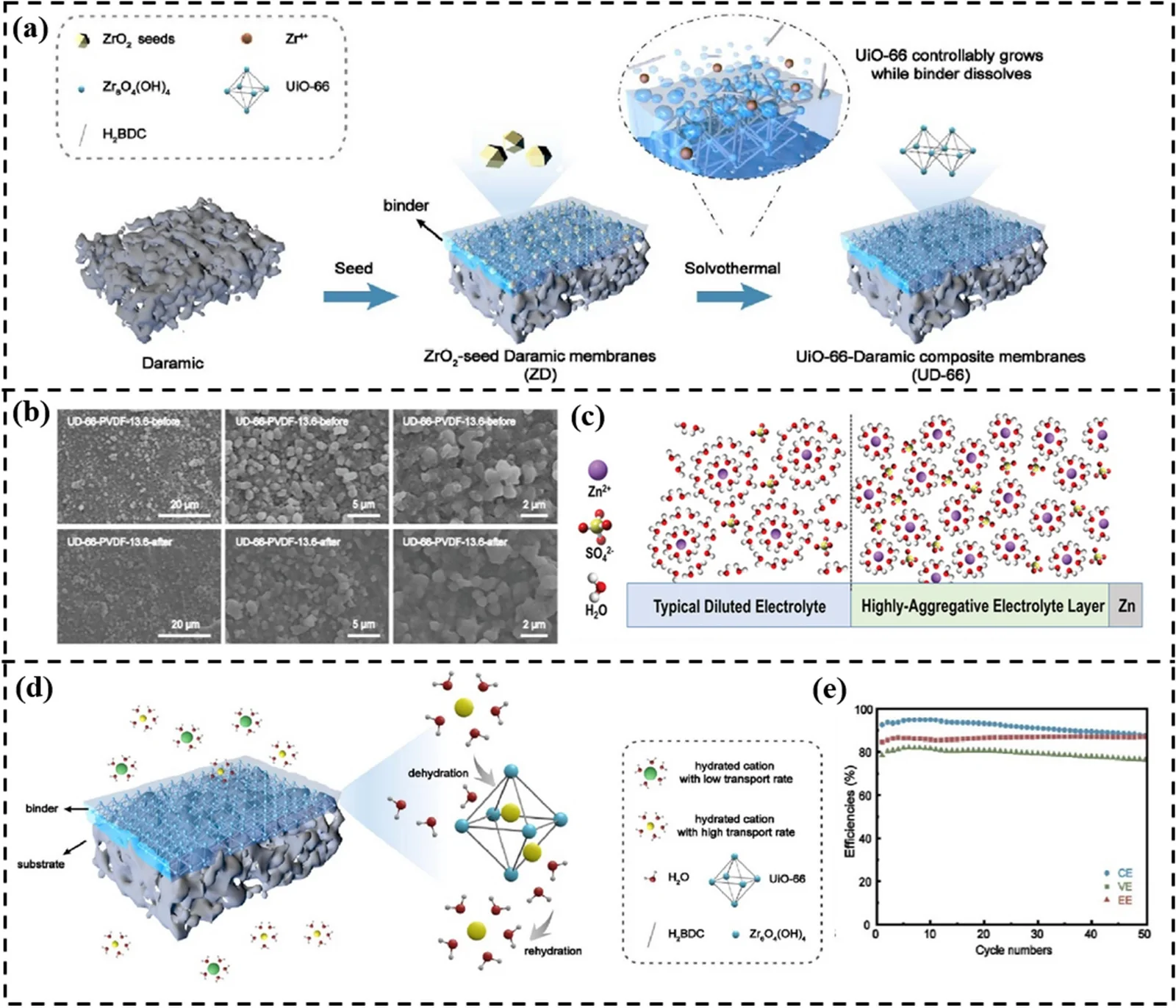
**a** Schematic diagram of Binder-controlled restrained second growth method [131], **b** SEM images of UD-66-PVDF-13.6 before and after cycling test [131]. Copyright 2021, Elsevier. **c** Highly aggregative electrolyte layer on Zn anode [42]. Copyright 2020, Wiley–VCH. **d** Schematic demonstration of passing of hydrated ion through UD-66 **e** cycling test of UD-66-PVDF-13.6 [131]. Copyright 2021, Elsevier

Wu et al. synthesized an ion-selective MOF UiO-66/-67 composite membrane by a binder-controlled restrained second growth method (BRSM), as shown in Fig. 22a. They proposed that the 6 or 8 Å windows in the UiO-66/-67 composite can effectively block I_3_^−^ owing to its large hydrated radius, successfully preventing its crossover. A schematic diagram of the passage of hydrated ions through UD-66 is shown in Fig. 22d. In addition, regular pore sizes contributed to the homogeneous Zn flux, minimizing dendrite formation. As a result, the optimized UD-66-PVDF-13.6 showed a high CE of 94.5%, EE of 81% at 80 mA cm^−2^, and long-term chemical/mechanical stability in the Zn-I_2_ redox flow battery. SEM images of UD-66-PVDF-13.6 before and after the cycling test are shown in Fig. 22b, whereas Fig. 22e shows its cycling test [131]. Zn-BTC MOF used as a separator in Zn-I_2_ battery also effectively suppressed the I_3_^−^ shuttle effect and reconstructed the electrolyte solvation structure near the anode, promoting highly aggregated contact ion pairs (CIP) and resulting in minimized HER, corrosion, and dendrites at Zn anode. A schematic diagram of a highly aggregative electrolyte layer near the Zn anode is shown in Fig. 22c that minimizes dendrite growth [42].

**Fig. 23:**
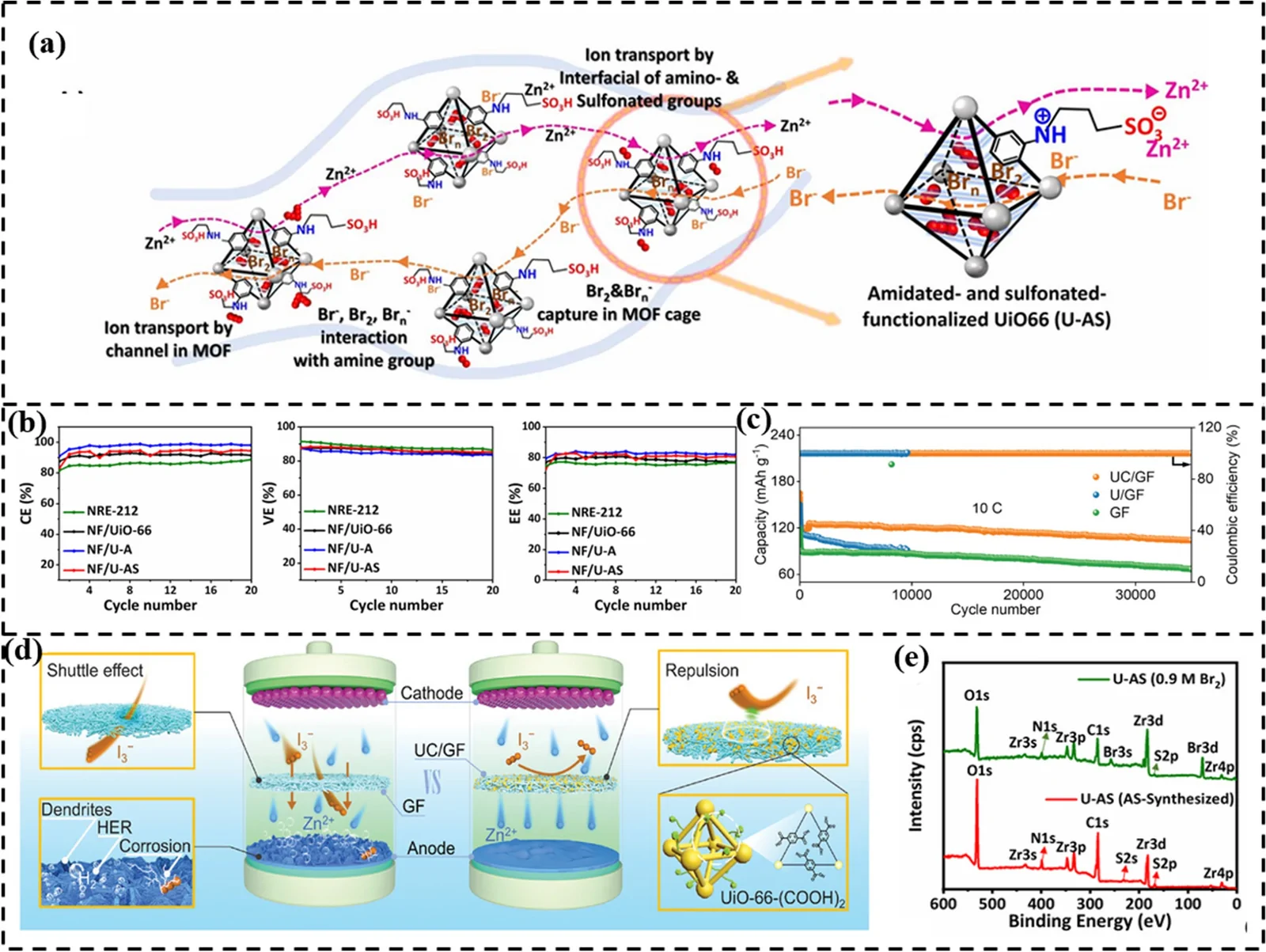
**a** Schematic illustration of ion transport and capture through U-AS membrane [133]. **b** CE, VE, and EE of NF/U-AS [133]. Copyright 2024, Royal Society of Chemistry. **c** Cycling test of UC/GF and **d** schematic diagram of Zn-I_2_ battery assembled with UC/GF separator [132]. Copyright 2024, Wiley–VCH. **e** XPS spectra of U-AS before and after hydrolytic stability test [133]. Copyright 2024, Royal Society of Chemistry

The strategic functionalization of MOFs presents a powerful design strategy for engineering high-performance separators for Zn-halogen batteries. For example, Yang et al. designed carboxyl-functionalized UiO-66 to modify a glass fiber (UC/GF) that behaved as a novel ionic separator. The hydrophilic and zincophilic properties of the carboxyl group enhance Zn^2+^ ion transport and promote the desolvation of hydrated zinc ions. This facilitated uniform Zn deposition while simultaneously suppressing HER and corrosion at the anode. Furthermore, the negatively charged carboxyl group exhibits electrostatic repulsion against polyiodide species, effectively suppressing their crossover and mitigating the shuttle effect. A schematic diagram of the Zn-I_2_ battery assembled using UC/GF as a separator is shown in Fig. 23d. The Zn-I_2_ battery assembled using UC/GF as a separator showed good performance while maintaining 103.8 mAh g^−1^ over 35,000 cycles at 10 C, as represented in Fig. 23c [132]. For Zn-Br_2_ flowless batteries, amidated and sulfonated UiO-66 supported on Nafion (NF/U-AS) was developed. Bromine crossover was suppressed via chemical binding of Br_2_/Br_n_^−^ to amine and physical confinement in MOF cages, while sulfonate groups facilitated balanced ion transport by forming abundant water channels, as shown in Fig. 23a. The NF/U-AS separator allowed Zn^2+^ ion transport and exhibited uniform Zn nucleation, while maintaining hydrolytic stability in corrosive environments (ZnBr_2_). XPS spectra of NF/U-AS before and after the hydrolytic stability test are shown in Fig. 23e, indicating no elemental leaching during immersion in ZnBr_2_. This multifunctional membrane maintained 98% CE for 5000 cycles at 10 mA cm^−2^ and stable operation at a 40% depth-of-charge DOC (1200 h with 79% CE). CE, VE, and EE performances of NF/U-AS in comparison with other combinations are shown in Fig. 23b [133].

**Fig. 24:**
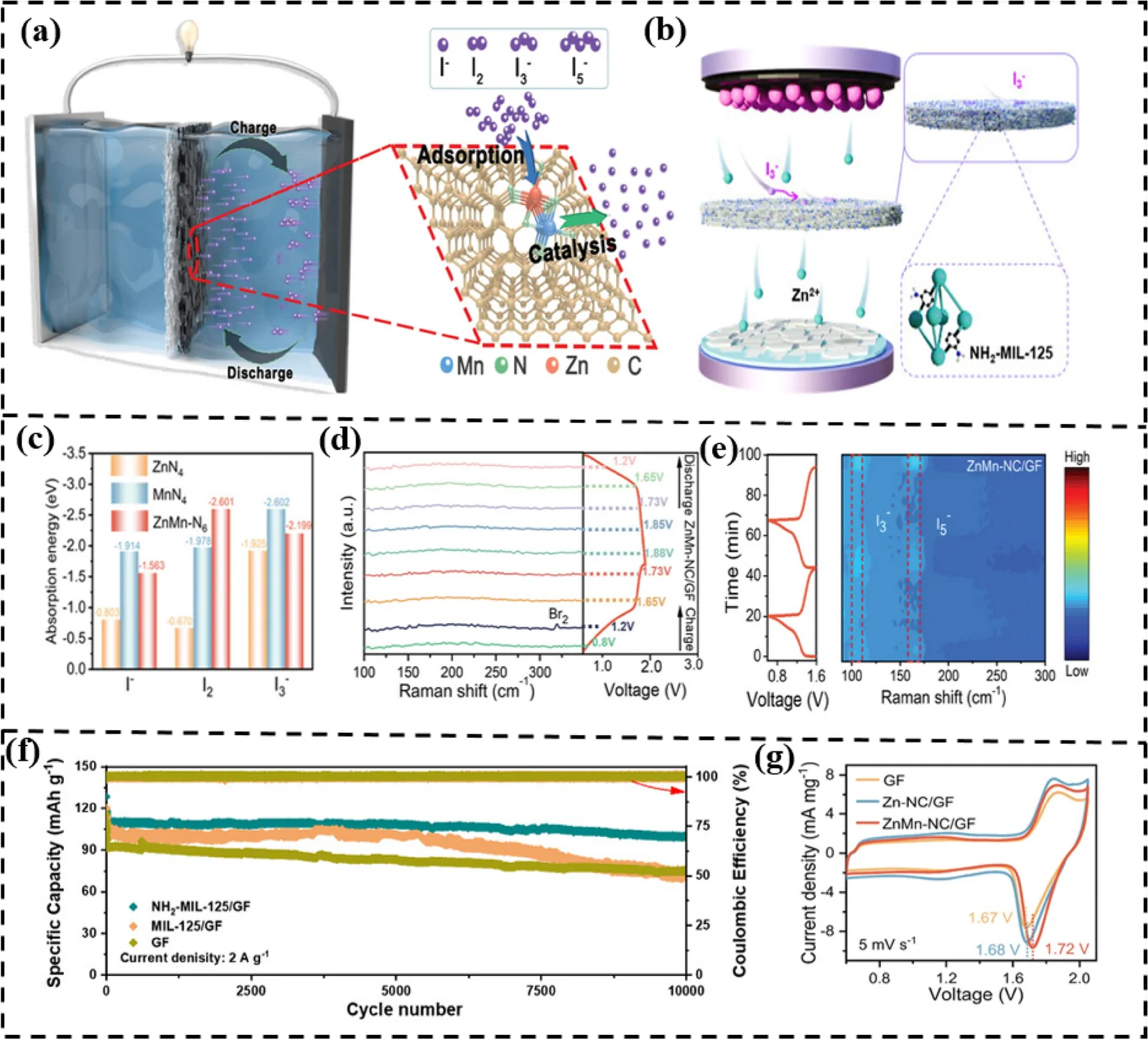
**a** Schematic demonstration of a battery assembled using ZnMn-NC/GF separator [52]. Copyright 2025, Wiley–VCH. **b** Full AZIB mechanism of Zn|NH_2_-MIL-125/GF|I_2_@TiO_2_/NPC [135]. Copyright 2025, Wiley–VCH. **c** Adsorption energy for iodine species on ZnN_4_, MnN_4_ and ZnMn-N_6_ sites [52]. In situ Raman spectra of: **d** Zn-Br_2_ and **e** Zn-I_2_battery [52]. Copyright 2025, Wiley–VCH. **f** Cycling performance of NH_2_-MIL-125/GF [135]. Copyright 2025, Wiley–VCH. **g** CV of Zn-Br_2_ battery assembled using ZnMn-NC/GF [52]. Copyright 2025, Wiley–VCH

The introduction of catalytic sites on the separator can enhance active material utilization and minimize self-discharge. Yang et al. developed an entrapment–adsorption–catalysis strategy on the separator by leveraging Zn-Mn atomic sites on modified glass fiber (ZnMn-NC/GF) and its performance was evaluated in Zn-halogen (Zn-I_2_ and Zn-Br_2_) battery systems. The operating mechanism of the battery assembled using the ZnMn-NC/GF separator is shown in Fig. 24a. Mn-N_4_ single sites were responsible for the adsorption of polyiodides/polybromides, while ZnMn-N_6_ active sites catalytically converted them back into active forms (I^−^/Br^−^) before reaching the Zn anode, enabling high utilization of cathode active species. The adsorption energies of iodine species on Mn-N_4_, Zn-N_4_, and ZnMn-N_6_ are shown in Fig. 24c, which indicate the strongest adsorption energies of I_3_^−^ and I^−^ on Mn-N_4_. The ZnMn-NC/GF separator significantly suppressed the shuttle effect. In situ Raman spectra of the Zn-Br_2_ and Zn-I_2_ batteries are shown in Fig. 24d, e, respectively, that were assembled using the ZnMn-NC/GF separator, where no noticeable peaks for polyiodide and polybromides were observed, except at the start of charging/discharging. As a result, the Zn-I_2_ battery assembled using ZnMn-NC/GF showed superior performance with a retention rate of 95.3% after 30,000 cycles at 5 A g^−1^, whereas the Zn-Br_2_ flowless battery could be operated for 3000 cycles at 5 A g^−1^. The CV curve of ZnMn-NC/GF in the Zn-Br_2_ battery is shown in Fig. 24g, which shows good reversibility and high voltage plateau [52].

Furthermore, Zhu et al. introduced a unique polar–nonpolar synergy for an aqueous Zn-I_2_ battery in which nonpolar porous carbon was used as an iodine host and ZIF-8-derived polar ZnO was used to modify the glass fiber (ZnO@GF). Porous carbon, owing to its large surface area and porosity, provides high iodine loading and improves cathode conductivity, whereas uniformly distributed ZnO on the separator facilitates rapid electron transport with polyiodides (I_3_^−^) through strong adsorption and catalytic effects, promoting reversible redox conversion of iodine species. This strategy simultaneously addresses several issues, including slow kinetics, polyiodide shuttling, and Zn corrosion [134].

Wang et al. reported a holistic “three-party synergistic” strategy, which demonstrated a comprehensive approach to synchronously stabilize the cathode, anode, and electrolyte in Zn-I_2_ batteries. The key innovation is the dual application of a single MOF precursor (NH_2_-MIL-125/GF), engineered into both a functional separator and a derived cathode host. The amine-functionalized MOF-coated separator electrostatically confines polyiodides, enables selective Zn^2+^ transport, and reduces interfacial resistance while the MOF-derived TiO_2_/NPC hierarchical host ensures efficient iodine encapsulation and conversion (Fig. 24b). This integrated system, complemented by anode interfacial engineering, yielded good full-cell (Zn|NH_2_-MIL-125/GF|I_2_@TiO_2_/NPC) performance, including a high capacity of 215 mAh g^−1^ and a remarkable 88% capacity retention over 10,000 cycles (Fig. 24f) [135].

**Fig. 25:**
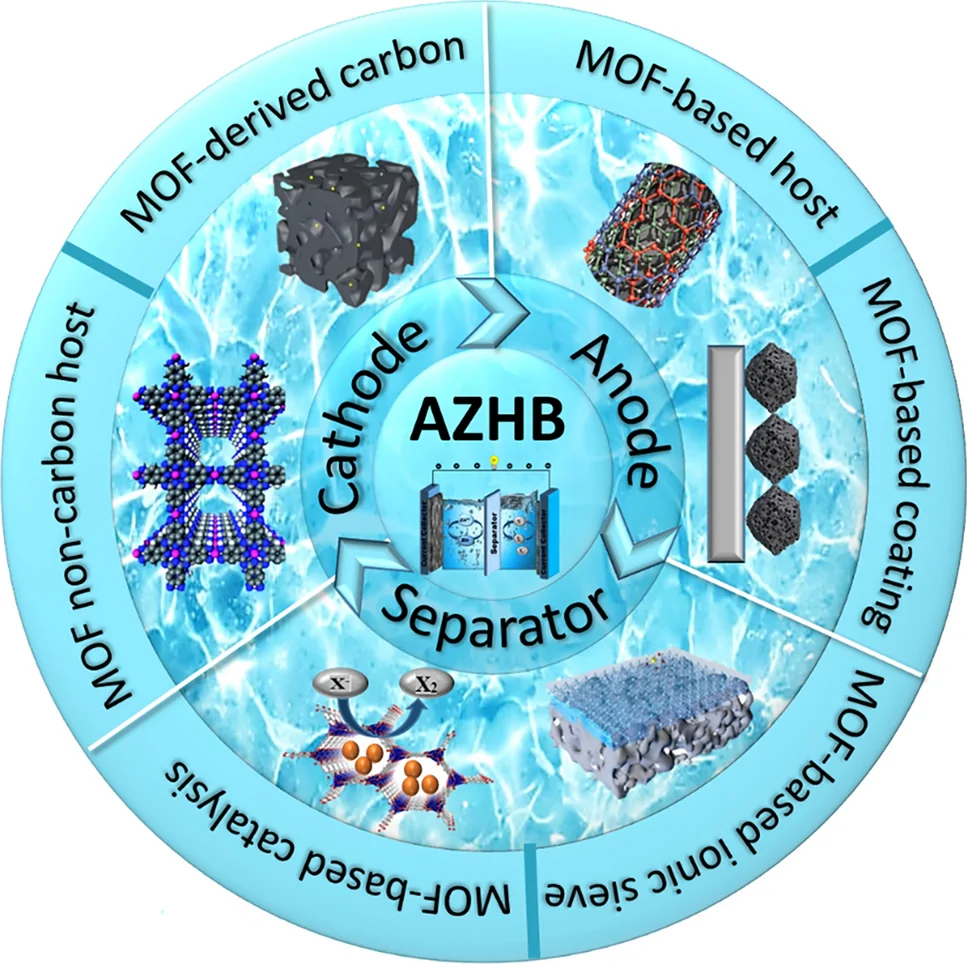
Schematic illustration of the diverse roles of MOFs in AZHBs

Overall, MOFs offer a uniquely versatile platform for advancing Zn-halogen batteries, with demonstrated success in enhancing performance through their roles as cathodes, anodes, interfacial modifiers, and separators. However, a recent study also reported on the role of MOF as an electrolyte additive in ZBFBs. This work utilized ZIF-62, not as a stable framework, but as a sacrificial precursor whose decomposition releases imidazole and benzimidazole ligands into the electrolyte. These ligands act synergistically with the conventional MEP-Br complexing agent, significantly enhancing the binding and sequestration of bromine species. In situ optical characterization also confirmed that this MOF-derived additive promotes the formation of a more uniform dispersion of bromine complexed oily droplets. This not only effectively suppresses the shuttle effect and improves reaction kinetics but also significantly mitigates the bromine-induced corrosion. ZBFBs employing this strategy achieved stability over 1500 cycles [148]. This demonstrates the potential of MOFs for electrolyte engineering. However, the research landscape on MOF-based strategies remains heavily skewed toward Zn-I_2_ systems, with significant fewer but rapidly expanding studies on Zn-Br_2_ configurations and almost no study on MOF-based Zn-Cl_2_ batteries. This gap is primarily due to the formidable challenges posed by chlorine electrochemistry, including the extreme corrosiveness of Cl_2_ and its intermediates, which can rapidly degrade conventional MOF frameworks and the difficulty in confining small, reactive chlorine molecules. Despite these hurdles, significant opportunities exist for pioneering MOFs in this nascent area. A schematic illustration of the multifunctional role of MOFs in AZHBs is shown in Fig. 25.

## Characterization Techniques for MOF-Based Zn-Halogen Batteries

**Fig. 26:**
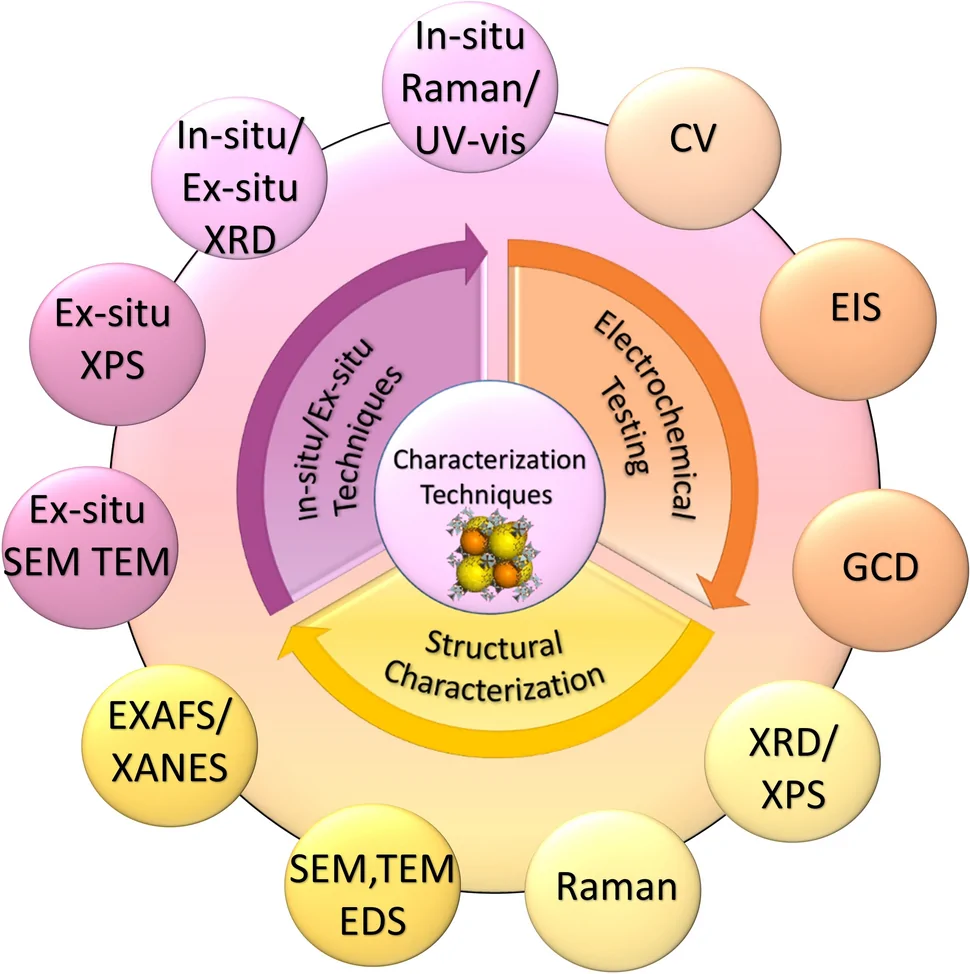
Schematic illustration of characterization techniques used for MOF-based Zn-halogen batteries

The development of MOF-based Zn-halogen batteries is significantly dependent on thorough characterization methods to determine their structural, morphological, and electrochemical features. In situ/ex situ are the advanced techniques to understand the mechanisms while electrochemical, and structural characterization techniques are conventional techniques for MOF-based Zn-halogen batteries, which are also summarized in Fig. 26. Collectively, these methods form critical structure–property correlations that guide the rational design of MOF-based electrodes and separators for high-performance Zn-halogen batteries**.**

### Advanced In Situ/Ex Situ Techniques

In situ and ex situ characterization techniques are crucial for understanding the electrochemical dynamic processes and structural evolution of MOF-based Zn-halogen batteries [45]. In situ methods allow for real-time monitoring of electrochemical reactions, phase transformations, and interfacial phenomena during battery cycling [57], whereas ex situ techniques are performed after electrochemical cycling to investigate long-term stability, degradation mechanisms, and compositional changes [48,59].

#### In Situ Raman Spectroscopy

**Fig. 27:**
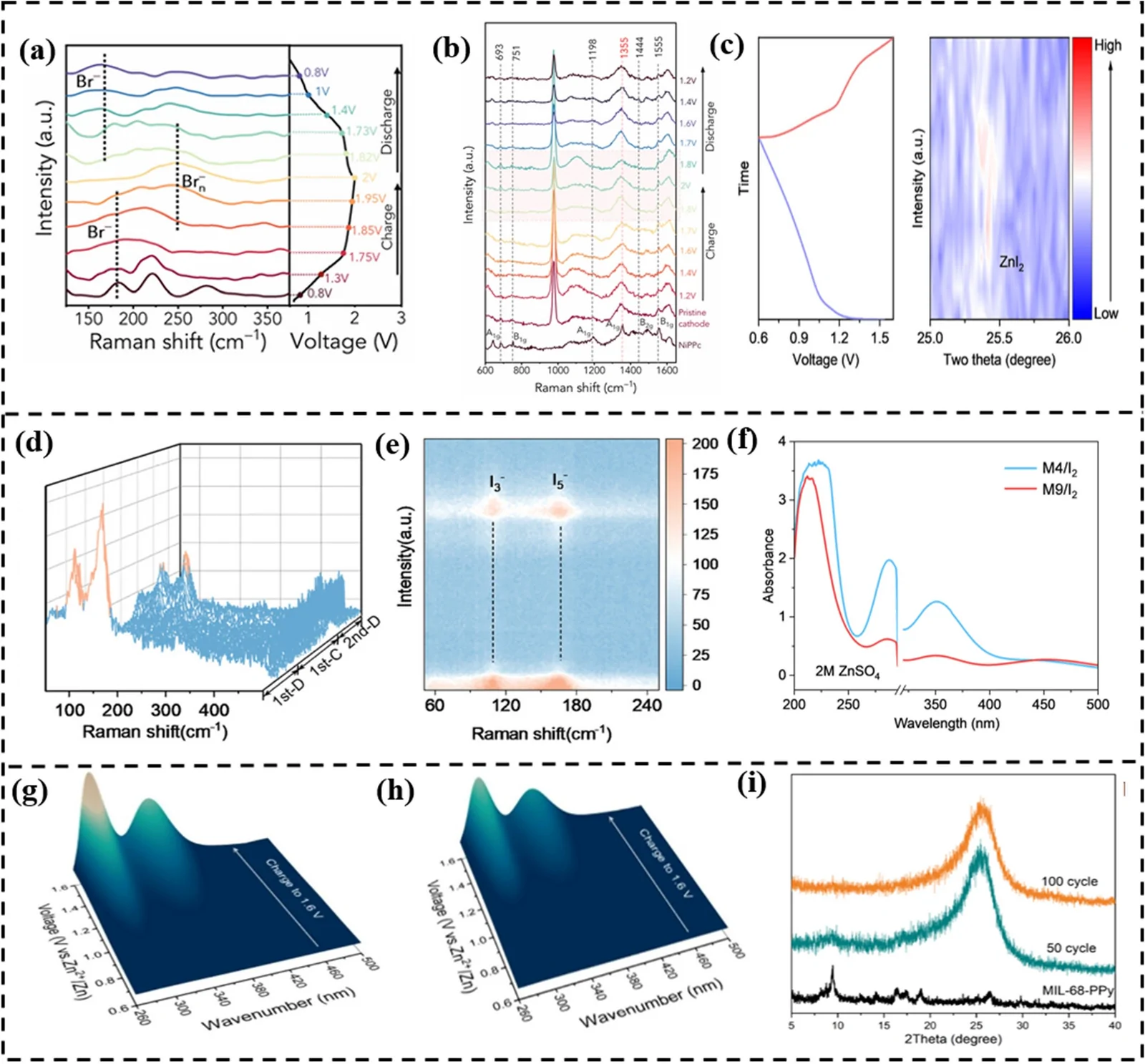
**a** In situ Raman spectra detection of Br species in the range of 125–375 cm^−1^ [50], **b** In situ Raman spectra detection of NiPPc in the range of 600–1650 cm^−1^ [50]. Copyright 2023, Royal Society of Chemistry, **c** in situ XRD spectrum [45]. Copyright 2025, Wiley–VCH, **d**, **e** in situ Raman analysis of I_2_@P2-1000 [96]. Copyright 2024, Wiley–VCH. **f** UV–vis spectra of M4/I_2_ and M9/I_2_ in 2 M ZnSO4 after 96 h [57]. Copyright 2024, Wiley–VCH. **g** In situ UV–vis spectra of I_2_@NC and **h** I_2_@SeSA-NC-900 [51]. Copyright 2025, American Chemical Society. **i** XRD spectrum of I_2_@MIL-68-PPy after 50 and 100 cycles [59]. Copyright 2025, Elsevier

In situ Raman spectroscopy has proved invaluable for elucidating the redox mechanism in MOF-based Zn-halogen batteries. By providing the vibrational modes of halogen species in real time, this technique offers critical insights into the reaction pathways and confinement behavior of MOF-based hosts [57]. For Zn-I_2_, an in situ Raman study of the MOF-based cathode (I_2_@P2-1000), as depicted in Fig. 27d, e, revealed two characteristic vibrational modes at 110 cm^−1^ (I_3_^−^) and 165 cm^−1^ (electron transfer between the carbon substrate and I_2_) that dynamically evolve during cycling. During discharge, the decreasing intensity of these peaks demonstrates the reduction of polyiodides and I_2_ to I^−^ (I_5_^−^→I_3_^−^→I^−^), while the reappearance during charging confirms the highly reversible conversion back to I_2_ [96]. In a Zn-Br_2_ battery employing a MOF-based host NiPPc, the in situ Raman analysis, at the electrode–electrolyte interface, elucidates the complex redox pathway. The pristine Br^−^ peak at 183 cm^−1^ shifts to a broad 170–300 cm^−1^ region during charging, indicating the formation of Br_3_^−^ or Br_n_^−^. The complete disappearance of the Br^−^/Br_3_^−^ peaks at full charge (2.0 V) demonstrates efficient conversion, while their gradual reappearance during discharge confirms the reversibility of the system (Fig. 27a). In addition, Fig. 27b shows the in situ Raman analysis of MOF’s Ni-N_4_ centers via peaks at 1355 and 1555 cm^−1^, revealing how these catalytic sites interact with polybromides while maintaining structural integrity [50].

#### In Situ and Ex Situ UV–VIS Spectroscopy

In situ and ex situ UV–vis spectroscopy has emerged as a powerful tool for investigating the redox chemistry and confinement effects in MOF-based Zn-halogen systems and complements in situ Raman findings [101]. For example, MOF-derived I_2_@Se_SA_-NC-900 showed a reduced I_3_^−^ peak intensity as compared to its undoped I_2_@NC counterpart, showing stronger confinement and a reduced shuttle effect, as indicated by the in situ UV/vis spectra of I_2_@NC and I_2_@Se_SA_-NC-900, as shown in Fig. 27g, h, respectively [51].

Ex situ UV–vis spectroscopy is a widely employed technique for investigating the halogen retention ability of MOF-based hosts after adsorption tests [45,57,101]. For example, iodine adsorption tests were performed on the Fe–N-C porous carbon host, M9/I_2_. Ex situ UV–vis spectra of M9/I_2_ (Fig. 27f) after adsorption tests showed no obvious I_3_^−^ peaks at 288.6 and 353.1 nm, reflecting its superior physicochemical confinement behavior [57].

#### In Situ/Ex Situ XRD

In situ XRD offers dynamic, real-time monitoring of the phase transitions and lattice changes during battery operation. For example, in situ XRD tracking of the Zn-I_2_ battery is shown in Fig. 27c, which reveals the formation of ZnI_2_ during charging that disappears upon discharging, indicating a reversible conversion mechanism. In contrast, the stable peak positions of MOF-derived hosts during cycling confirm their structural robustness [51]. Similarly, ex situ XRD is often employed to examine the crystalline integrity and phase composition of MOF-based material before and after electrochemical testing [59,131]. For example, ex situ XRD analysis was employed on the I_2_@MIL-68-PPy electrode after 50 and 100 electrochemical cycles, where a noticeable broadening of the peaks was observed after repeated cycling, as shown in Fig. 27i. This broadening indicates the structural collapse and amorphization of the material, which is attributed to the repeated intercalation/deintercalation of iodine [59].

#### Ex Situ XPS

Ex situ XPS is a surface-sensitive technique that provides vital insights into the halogen adsorption behavior, redox reaction pathways, and stability of materials [74]. For instance, iodine loading onto Co-SAs@NPC produces a noticeable shift in the Co 2*p*, N 1*s*, and O 1*s* peaks, indicating chemisorption of iodine species on the Co-N_4_ active sites. In addition, high-resolution XPS enables the identification of iodine redox states at various charge/discharge states, providing insights into the redox mechanism and reversibility [74].

#### Ex Situ Morphological Analysis

Ex situ morphological analysis using SEM, TEM, and EDS provides critical insights into the structural evolution and degradation mechanism of MOF-based materials in Zn-halogen batteries. For NiCo-MOF@GF, post-cycling SEM and TEM analysis revealed the electrode surface was fully coated with residual ZnBr_2_ salts and polybromide complexes after 200 cycles, suggesting ongoing halogen redox reactions. However, the sheet-like morphology of NiCo-MOF@GF was preserved after cycling [48]. Ex situ morphological analysis also provides useful insights into Zn deposition behavior [130].

### Conventional Electrochemical Testing

#### Cyclic Voltammetry CV

**Fig. 28:**
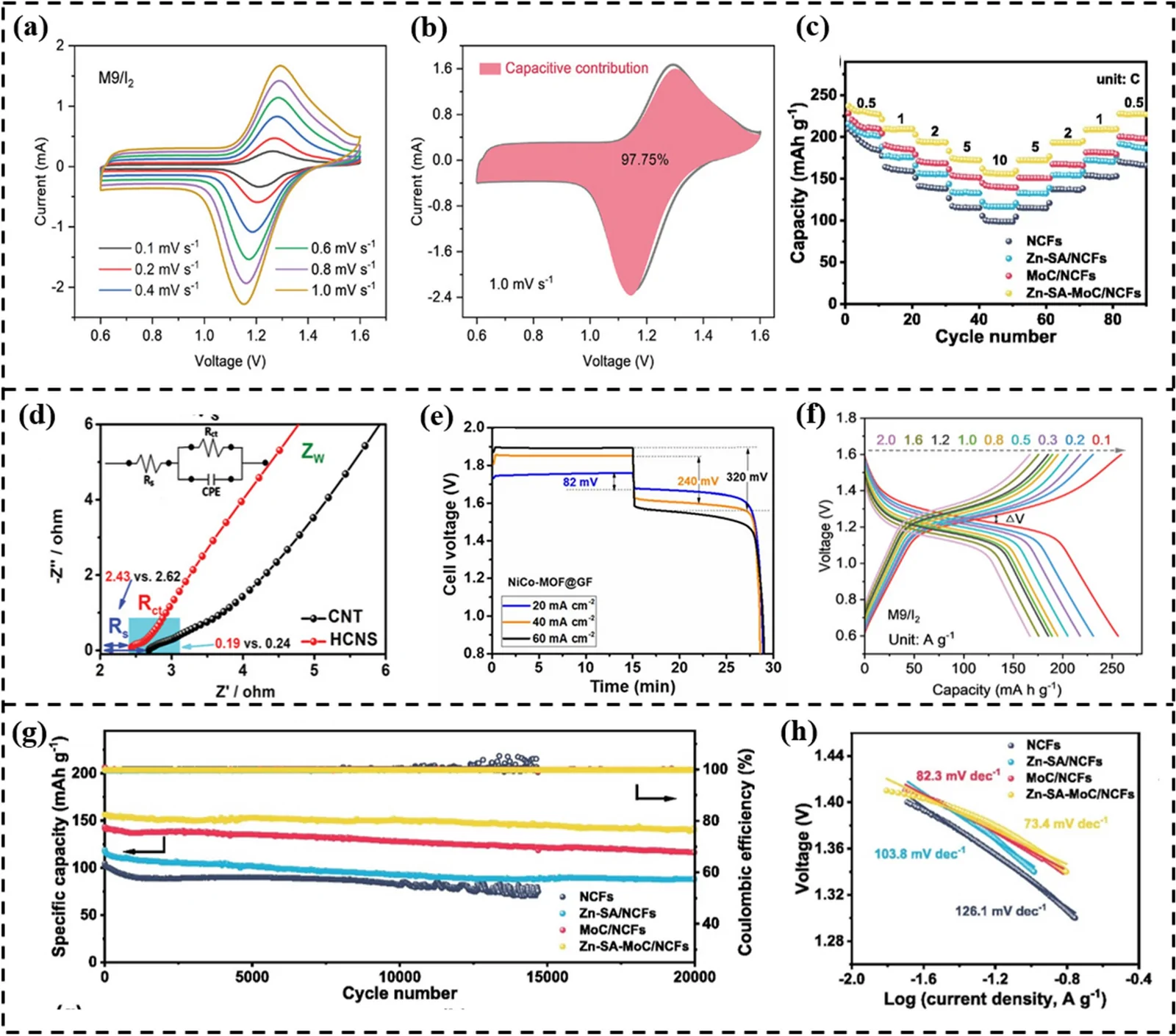
**a** CV curve and **b** capacitive contribution of M9/I_2_ [57]. Copyright 2024, Wiley–VCH. **c** Rate capability test [122]. Copyright 2025, Elsevier. **d** Nyquist plot of HCNS [110]. Copyright 2022, Wiley–VCH. **e** GCD curve of redox flow Zn-Br_2_ battery [48]. **f** GCD curve of static Zn-I_2_ battery [57]. Copyright 2024, Wiley–VCH. **g** Cycling test, **h** Tafel slopes [122]. Copyright 2025, Elsevier

CV is a crucial electrochemical technique that is widely employed to elucidate the redox behavior, reaction kinetics, and charge storage mechanism in MOF-based Zn-halogen batteries. Typically, the redox peaks observed in the CV curves correspond to reversible halogen redox couples, and the peak separation (ΔE) indicates electrochemical reversibility and polarization [110]. MOF-based cathodes, especially those with catalytic sites and N doping, exhibit narrow peak separation and lower Tafel slopes, reflecting enhanced redox kinetics and reduced overpotentials. The Tafel slopes of iodine redox reactions on various catalysts are represented in Fig. 28h, whereas the CV plot of Fe–N–C (M9/I_2_) at various scan rates is shown in Fig. 28a [57,105]. The kinetic behavior can be further quantified as *i* = av^b^, where the b-value (calculated from the log i-log v curve) close to 1 indicates a surface capacitive-controlled process, while lower values indicate diffusion-controlled processes [45]. For example, an optimized MOF cathode, such as M9/I_2_, achieves 97.75% capacitive contribution at 1 mV s^−1^, as shown in Fig. 28b, which represents its superior kinetics [57].

#### Electrochemical Impedance Spectroscopy EIS

EIS is a powerful technique used to analyze the charge transfer resistance and ion diffusion behavior of MOF-based materials in Zn-halogen batteries. The Nyquist plot typically consists of a semicircle in the high-frequency region, which represents charge transfer resistance (*R*_ct_) and a linear line in the low-frequency region associated with Warburg impedance (*Z*_*w*_), indicating ion diffusion [48]. Nyquist plot of MOF-derived material like HCNS is shown in Fig. 28d, which exhibits significantly lower *R*_ct_ (0.19 Ω), *R*_*s*_ (2.43 Ω) and steeper Warburg slopes, suggesting improved conductivity [110]. The equivalent circuit model is used to fit the EIS data and typically includes elements such as solution resistance *R*_s_**,** electrode–electrolyte interfacial resistance R_EL_**,** *R*_ct_, and *Z*_*w*_. Accurate fitting of these components allows the quantification of individual resistive elements [48].

#### Galvanostatic Charge–Discharge GCD

GCD testing is a fundamental electrochemical technique used to evaluate the performance of MOF-based Zn-halogen batteries. This provides critical insights into the specific capacity, energy efficiency, overpotential, and cycling stability [48,74]. In Zn-halogen batteries, the GCD curves typically exhibit flat voltage plateaus corresponding to the redox reaction of halogen species, indicating stable and reversible redox processes [74]. GCD profile of NiCo-MOF@GF at various current densities is shown in Fig. 28e, which was employed as a cathode in a Zn-Br_2_ redox flow battery, whereas Fig. 28f shows the GCD curve of the M9/I_2_ cathode in a static Zn-I_2_ battery [48,57]. Furthermore, long-term GCD cycling provides information about the cycling stability, and analyzing GCD data across various current densities indicates the rate capability of MOF-based materials [57,101]. The cycling stability test of the Zn-SA-MoC/NCFs is shown in Fig. 28g, whereas Fig. 28c shows the rate capability test.

### Conventional Structural and Morphological Characterization

**Fig. 29:**
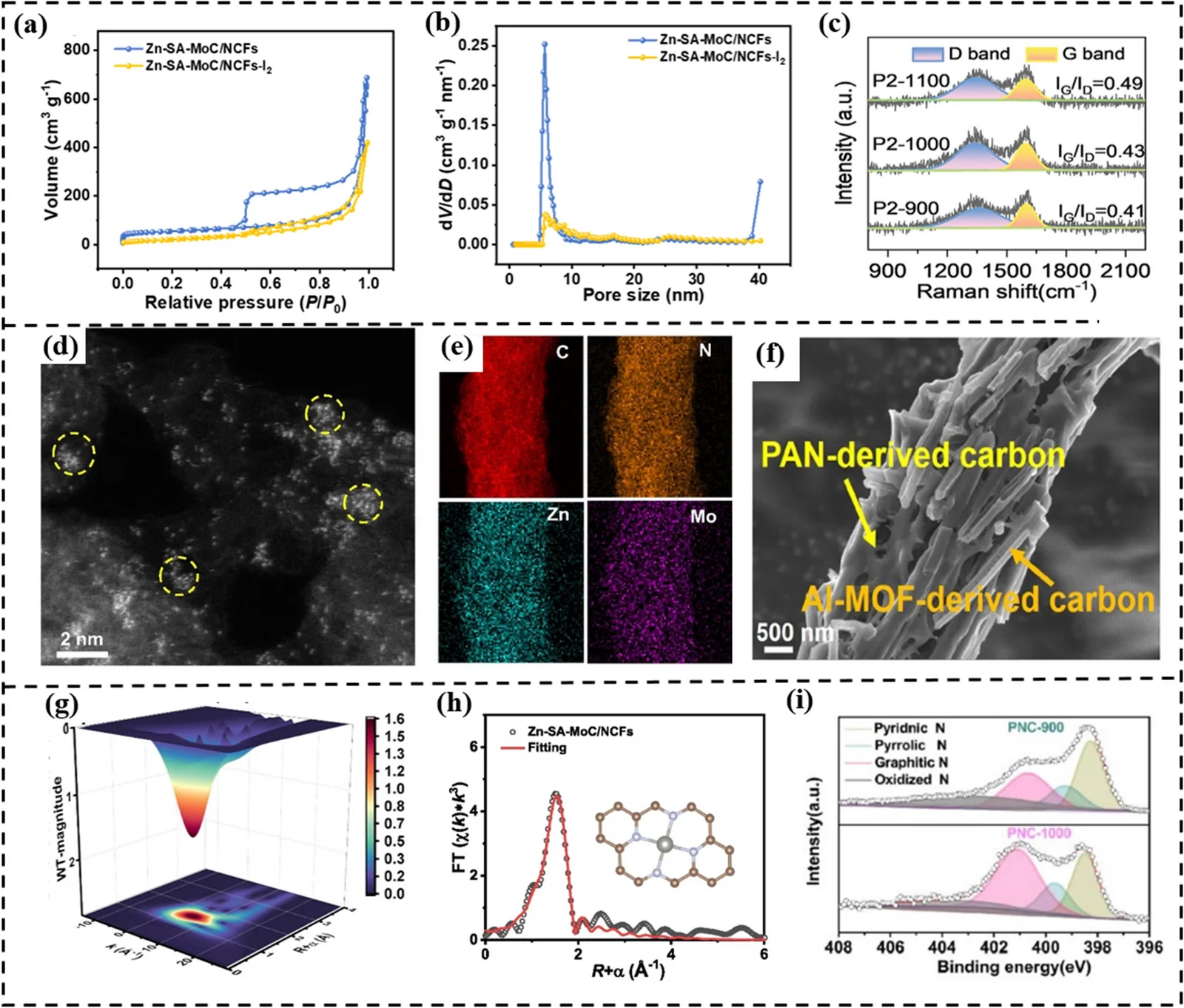
**a** N_2_ adsorption–desorption isotherms and **b** pore size distribution of Zn-SA-MoC/NCFs and Zn-SA-MoC/NCFs-I_2_ [122]. Copyright 2025, Elsevier. **c** Raman spectra of P2-1000 [96]. Copyright 2024, Wiley–VCH. **d** HAADF-STEM of Zn-SA-MoC/NCFs and **e** EDS mapping of C, N, Zn, and Mo [122]. Copyright 2025, Elsevier. **f** SEM image of PAN- and Al-MOF-derived carbon [99]. Copyright 2022, Springer Nature. **g** Wavelet transformation EXAFS plot, and **h** EXAFS fitting R space of Zn-SA-MoC/NCFs [122]. Copyright 2025, Elsevier. **i** XPS spectra of N [106]. Copyright 2022, Elsevier

The structural and morphological characterization of MOF-based materials, including their derivatives, is critical for understanding their role in enhancing the electrochemical performance of Zn-halogen batteries. For instance, XRD is often employed to verify crystallinity and phase integrity before and after thermal treatment. After carbonization, broad diffraction peaks around 25° and 44°, corresponding to the (002) and (101) planes, respectively, confirm the formation of amorphous carbon [74,102]. Raman spectroscopy further reveals the degree of graphitization and defect density by comparing D-band and G-band intensities (I_D_/I_G_), where high values indicate high defects, while low values indicate a higher graphitization degree. Raman spectra and I_D_/I_G_ ratio of P2-900, P2-1000, and P2-1100 electrodes are shown in Fig. 29c [51,96,101,110].

Electron microscopy reveals the surface morphology, microstructure, and structural integrity. SEM imaging reveals the evolution of morphology during synthesis [98,101]. SEM image of PAN-derived carbon and Al-MOF-derived carbon is shown in Fig. 29f [99]. TEM and HR-TEM analyses indicate the formation of pores and an amorphous structure after carbonization [101]. EDS mapping further verifies the homogeneous elemental distribution of key components such as C, N, and metal species throughout the material [50]. XPS provides essential chemical surface analysis of MOF-based Zn-halogen batteries, revealing key information about the elemental composition, bonding states, and oxidation states, which determine metal oxidation states and their coordinative environments [51], nitrogen configurations [99,106], and halogen species [74]. High-resolution XPS spectra of N are shown in Fig. 29i, which indicates the presence of pyridinic-N, pyrrolic-N, graphitic-N, and oxidized-N [106].

N_2_ adsorption–desorption isotherms and pore size distribution analysis confirmed the hierarchical porous structures (micro-, meso-, and macropores), which are essential for electrolyte penetration, halogen confinement, and mitigating shuttle effects. It also provides information about the decrease in surface area after iodine loading. N_2_ adsorption–desorption isotherm is shown in Fig. 29a, whereas Fig. 29b shows the pore size distribution of the Zn-SA-MoC/NCFs. The surface area and pore volume decreased after iodine loading onto Zn-SA-MoC/NCFs-I_2,_ as shown in Fig. 29a, b [57,101,102,122].

High-angular dark-field scanning transmission electron microscopy (HAADF-STEM) and energy-dispersive *X*-ray spectroscopy (EDX) are indispensable for SAC characterization. These techniques reveal atomic-level dispersion of single metal atoms (e.g., Zn, Se, Fe) within nitrogen-doped carbon matrices and confirm uniform elemental distribution throughout the host [51,57]. HAADF-STEM of Zn-SA-MoC/NCFs is shown in Fig. 29d, in which bright spots circled by yellow circles indicate single atoms, and Fig. 29e shows the corresponding EDS mapping [122]. *X*-ray absorption fine structure spectroscopy (XAFS), including *X*-ray absorption near edge structure spectroscopy (XANES) and extended *X*-ray absorption fine structure (EXAFS), is used to determine the local coordination environments [57,122]. Wavelet transformation EXAFS is shown in Fig. 29g, whereas Fig. 29h shows the EXAFS fitting R space of Zn-SA-MoC/NCFs, which reveals the Zn-N_4_ coordination environment [122].

## DFT Insights for MOF-Based Zn-Halogen Batteries

DFT has emerged as an indispensable tool for elucidating atomic-scale mechanisms and for guiding rational material design. DFT plays a crucial role in MOF-based Zn-halogen batteries by providing insights into the interaction of halogens with the host, the reaction mechanism, charge transfer dynamics, and the electronic structure of MOFs [57,107].

### Halogen Interactions

**Fig. 30:**
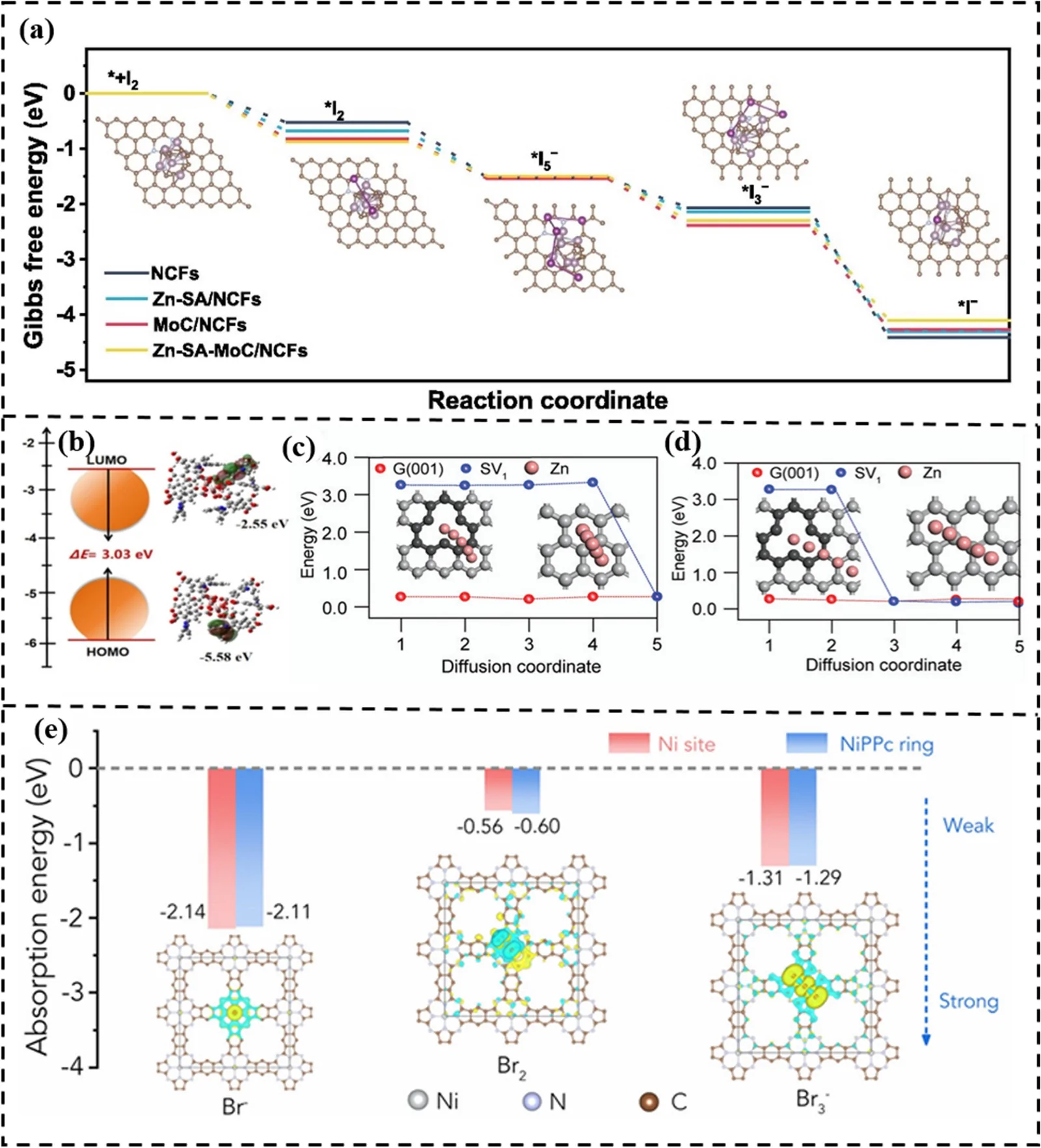
**a** Gibbs free energy diagram for I_2_/I^−^ conversion on Zn-SA-MoC/NCFs [122]. Copyright 2025, Elsevier. **b** HOMO–LUMO of MIL-68-PPy [59]. Copyright 2025, Elsevier. **c** Changes in the energy profiles of Zn on the SV1 defect-containing carbon surface with movement of the adsorbed Zn atom crossing the C–C bond and **d** along the C–C bond [130]. Copyright 2020, Royal Society of Chemistry. **e** Charge density difference diagram of Br species around Ni–N_4_ sites [50]. Copyright 2023, Royal Society of Chemistry

By calculating the adsorption energies between polyhalide ions and the MOF framework, DFT helps identify active sites and heteroatom dopants that maximize polyhalide adsorption and suppress the shuttle effect [51,122]. For example, graphitic-N sites can chemically immobilize iodine species, thereby enabling reversible redox activity. Charge density difference maps after adsorption also provide insights into the charge transfer between polyhaides and heteroatoms, confirming their strong covalent interactions [57,106]. For example, Fig. 30e shows a charge density difference map following Br species adsorption, where electron depletion is represented by blue and electron accumulation is represented in yellow. The pronounced electron accumulation at the interface indicates substantial electron transfer between Ni-N_4_ and Br species [50].

### Mechanistic Understanding

Gibbs free energy calculations can elucidate the catalytic pathways for the Br_2_ and I_2_ reduction reactions, identifying optimal electrocatalysts with lower overpotentials [50,51]. Gibbs free energy diagram of iodine conversion is shown in Fig. 30a, which shows a reduced Gibbs free energy for Zn-SAs-MoC/NCFs, indicating its favorability in iodine conversion [122]. Spin density calculations further highlight the role of transition metals in facilitating electron transfer, explaining enhanced kinetics in Fe or Co-modified hosts [57,74].

### Electronic Structure Analysis

DFT-based frontier molecular orbital (FMO) analysis, which examines the highest occupied molecular orbital (HOMO) and lowest unoccupied molecular orbital (LUMO), provides critical insights into the electronic structure. The HUMO-LUMO band gap is directly related to electronic conductivity; a low band gap indicates better electron mobility and faster kinetics [122]. HOMO–LUMO analysis of MIL-68-PPy shows that integration of polypyrrole in MOF has reduced the band gap to 3.03 eV (Fig. 30b) that balances conductivity and stability [59].

### Understanding Zinc Deposition Mechanism

DFT plays a crucial role in understanding the Zn deposition at the atomic scale. By simulating the adsorption energy and diffusion barriers, DFT revealed how different electrode surfaces influence Zn nucleation and growth [52]. For instance, calculations show that defects such as single vacancies in carbon-based materials significantly enhance the Zn binding strength compared to pristine surfaces, promoting uniform nucleation. Additionally, DFT predicts diffusion barriers that determine whether Zn adatoms remain immobilized or migrate to form dendrites. The adsorption energy changes in the energy profiles of Zn on the single-vacancy defect-containing carbon surface with movement of the adsorbed Zn atom crossing the C–C bond and along the C–C bond are shown in Fig. 30c, d, respectively [130].

## Summary and Future Outlook

**Fig. 31:**
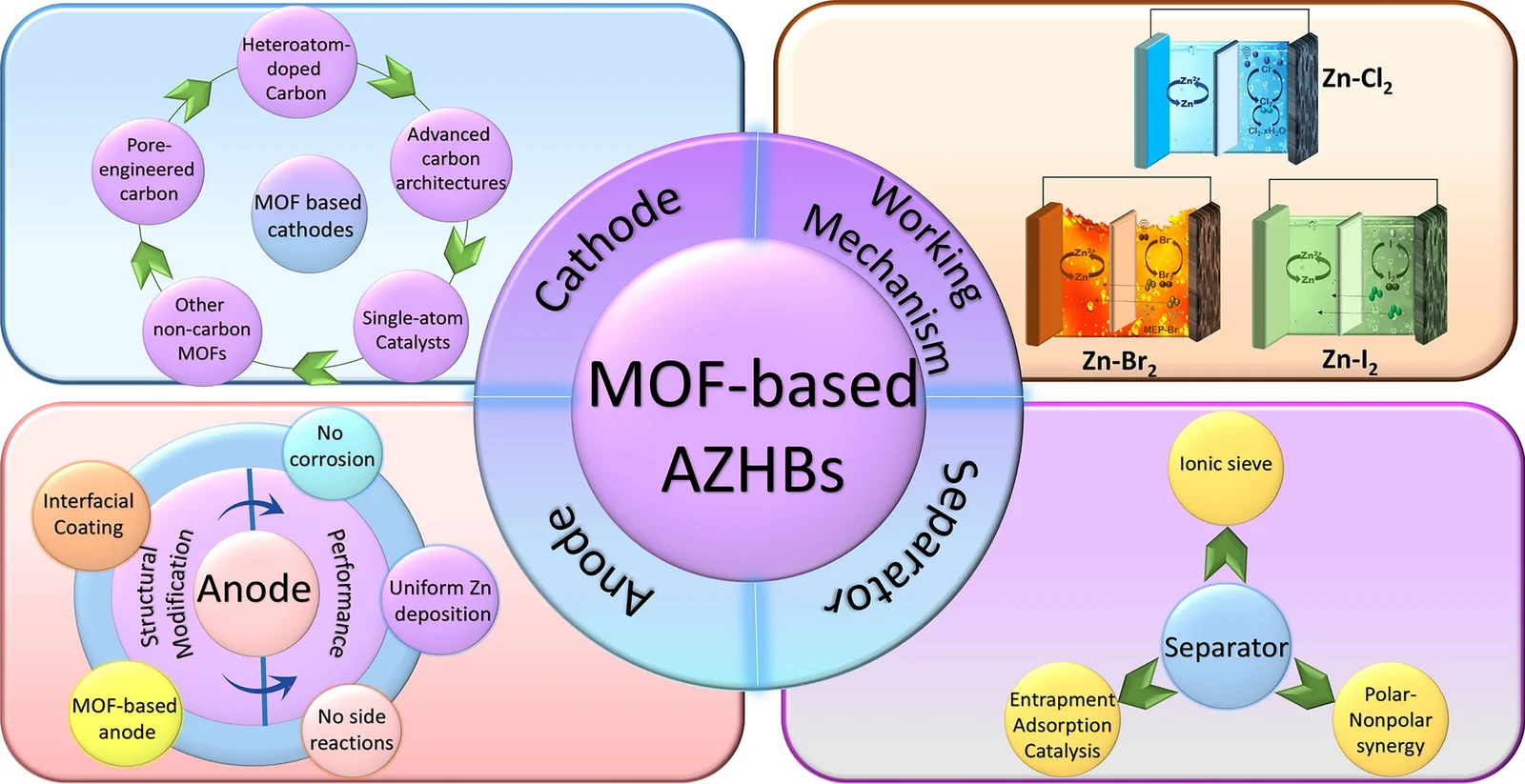
Summary of MOF-based AZHBs

MOFs have demonstrated remarkable potential for revolutionizing Zn-halogen batteries by addressing their most critical challenges. Through their precisely tunable porous structures, MOFs act as superior hosts, effectively confining halogen species and suppressing the shuttle effect, significantly improving the cycling stability. The incorporation of catalytic sites within MOF structures enhances the redox kinetics, reduces polarization, and increases efficiency. MOF-derived carbon materials combine high conductivity with preserved porosity, enabling efficient electron transfer and halogen storage, whereas non-carbon-based MOF hosts have also shown potential for future optimization. Furthermore, MOFs have shown significant potential for minimizing dendrite formation and suppressing corrosion at anodes. In separators, metal–organic frameworks (MOFs) offer the dual benefits of inhibiting the shuttle effect and regulating zinc deposition. These MOF-based innovations have significantly improved the energy density, cycle life, and rate capability of Zn-halogen batteries. The summary of role of MOFs in Zn-halogen batteries is represented in Fig. 31.

However, challenges exist for using MOFs as functional materials in Zn-halogen batteries, including the following:

1. The multi-step, high-temperature synthesis required for high-performance MOF-derived carbons and SACs is energy-intensive and costly, presenting a significant barrier to large-scale manufacturing.
2. Many MOFs are susceptible to pore collapse, lattice distortion, and chemical degradation in the presence of moisture, acidic electrolytes, and highly oxidizing halogen species, leading to irreversible capacity fade.
3. Most reported high performances are achieved at low active mass loadings, failing to demonstrate feasibility in practical, high-energy-density cells.
4. The poor electronic conductivity of most pristine MOFs, stemming from the insulating organic linkers, results in high charge transfer resistance and sluggish reaction kinetics, necessitating additional conductive additives or post-synthetic treatments.
5. While MOFs significantly mitigate the polyhalide shuttle, the physical and chemical confinement is not absolute, allowing gradual active material loss and anode corrosion over thousands of cycles, especially under high rates and loadings.

Future research should focus on the following aspects:

1. The success of advanced MOF designs the way forward, future efforts must focus on designing MOFs that are intrinsically conductive and stable, moving beyond energy-intensive derivatization. This involves exploring redox active or conjugated linkers and hydrolytically robust metal nodes to create frameworks that withstand harsh halogen environments without compromising performance.
2. The demonstration of a four-electron transfer process in Fe SAC-MNC and In-MOF/MPII systems unveils a new frontier. Future efforts must focus on designing MOFs with ultra-stable, conductive frameworks specifically tailored to stabilize the highly reactive I^+^ intermediate.
3. To overcome conductivity limitations and create synergistic effects, composite materials engineering is a key strategy. The enhanced performance and suppressed shuttle effect of MOF/MXene composites in sulfur hosts [149] suggest a direct pathway for suppressing shuttle effect and enhancing conversion kinetics in Zn-halogen systems.
4. The successful demonstration of flexible soft pack batteries using Fe-N-C cathodes proves practical potential. The next step is to bridge the lab-to-industrial gap by developing scalable synthetic routes and integrating MOF components from anodes to separators into commercially viable device architectures.
5. The innovative use of ZIF-62 as a sacrificial additive redefines the role of MOFs from static components to dynamic electrolyte modulators. This “reactive MOF” strategy offers a powerful new approach to mitigating halogen volatility. Future efforts should focus on screening and designing MOF precursors for controlled decomposition, releasing optimized ligands that synergistically enhance halogen sequestration. Concurrently, this concept of MOF-based electrolyte could be extended to develop robust MOF-based solid-state electrolytes, leveraging their molecular-sieving properties to physically eliminate the polyhalide shuttle effect entirely.
6. The extreme corrosiveness of chlorine has rendered Zn-Cl_2_ batteries a virtually unexplored frontier. Future pioneering work should explore ultra-robust MOF platforms (e.g., Zr or Al-based MOFs known for high oxidation resistance) as conductive and catalytic hosts for chlorine sequestration and conversion. Success in this area could unlock a new generation of high-energy-density halogen batteries.
7. The vast chemical space of MOFs is too large for traditional trial and error. Future work should leverage the growing dataset of MOF performances to train machine learning models. These models can predict optimal structures for specific halogens, dramatically accelerating the identification of high-performance candidates like the asymmetric Co-N_3_P_1_ site before synthesis.
8. As the field matures, the environmental impact of MOF synthesis and disposal must be a central concern. Future work should aim to develop green synthesis routes and design MOFs with recyclable components, ensuring the technology’s sustainability.

## References

[1] S. Nyamathulla, C. Dhanamjayulu, A review of battery energy storage systems and advanced battery management system for different applications: challenges and recommendations. J. Energy Storage **86**, 111179 (2024). 10.1016/j.est.2024.111179

[2] Y. Wang, H. Xiang, Y.-Y. Soo, X. Fan, Aging mechanisms, prognostics and management for lithium-ion batteries: recent advances. Renew. Sustain. Energy Rev. **207**, 114915 (2025). 10.1016/j.rser.2024.114915

[3] T. Kim, W. Song, D.-Y. Son, L.K. Ono, Y. Qi, Lithium-ion batteries: outlook on present, future, and hybridized technologies. J. Mater. Chem. A **7**(7), 2942–2964 (2019). 10.1039/c8ta10513h

[4] N. Dong, F. Zhang, H. Pan, Towards the practical application of Zn metal anodes for mild aqueous rechargeable Zn batteries. Chem. Sci. **13**(28), 8243–8252 (2022). 10.1039/d2sc01818g 35919714 10.1039/d2sc01818g PMC9297528

[5] G. Li, L. Sun, S. Zhang, C. Zhang, H. Jin et al., Developing cathode materials for aqueous zinc ion batteries: challenges and practical prospects. Adv. Funct. Mater. **34**(5), 2301291 (2024). 10.1002/adfm.202301291

[6] H. Yan, S. Li, J. Zhong, B. Li, An electrochemical perspective of aqueous zinc metal anode. Nano-Micro Lett. **16**(1), 15 (2023). 10.1007/s40820-023-01227-x 10.1007/s40820-023-01227-x PMC10656387 37975948

[7] C. Zhou, Z. Ding, S. Ying, H. Jiang, Y. Wang et al., Electrode/electrolyte optimization-induced double-layered architecture for high-performance aqueous zinc-(dual) halogen batteries. Nano-Micro Letters **17**(1), 58 (2024). 10.1007/s40820-024-01551-w 39509032 10.1007/s40820-024-01551-w PMC11544112

[8] T. Wang, C. Li, X. Xie, B. Lu, Z. He et al., Anode materials for aqueous zinc ion batteries: mechanisms, properties, and perspectives. ACS Nano **14**(12), 16321–16347 (2020). 10.1021/acsnano.0c07041 33314908 10.1021/acsnano.0c07041

[9] M. Song, H. Tan, D. Chao, H.J. Fan, Recent advances in Zn-ion batteries. Adv. Funct. Mater. **28**(41), 1802564 (2018). 10.1002/adfm.201802564

[10] B. Wang, C. Guan, Q. Zhou, Y. Wang, Y. Zhu et al., Screening anionic groups within zwitterionic additives for eliminating hydrogen evolution and dendrites in aqueous zinc ion batteries. Nano-Micro Lett. **17**(1), 314 (2025). 10.1007/s40820-025-01826-w 10.1007/s40820-025-01826-w PMC12202267 40569324

[11] T. Wang, Y. Zhang, J. You, F. Hu, Recent progress in aqueous zinc-ion batteries: from fundamentalscience to structure design. Chem. Rec. **23**(5), e202200309 (2023). 10.1002/tcr.202200309 36974578 10.1002/tcr.202200309

[12] Y. Yan, Y. Zhang, Y. Wu, Z. Wang, A. Mathur et al., A lasagna-inspired nanoscale ZnO anode design for high-energy rechargeable aqueous batteries. ACS Appl. Energy Mater. **1**(11), 6345–6351 (2018). 10.1021/acsaem.8b01321

[13] Y. Tian, Y. An, C. Wei, B. Xi, S. Xiong et al., Recent advances and perspectives of Zn-metal free “rocking-chair”-type Zn-ion batteries. Adv. Energy Mater. **11**(5), 2002529 (2021). 10.1002/aenm.202002529

[14] J. Hao, S. Zhang, H. Wu, L. Yuan, K. Davey et al., Advanced cathodes for aqueous Zn batteries beyond Zn^2+^ intercalation. Chem. Soc. Rev. **53**(9), 4312–4332 (2024). 10.1039/d3cs00771e 38596903 10.1039/d3cs00771e

[15] D. Chao, W. Zhou, C. Ye, Q. Zhang, Y. Chen et al., An electrolytic Zn–MnO_2_ battery for high-voltage and scalable energy storage. Angew. Chem. Int. Ed. **131**(23), 7905–7910 (2019). 10.1002/ange.201904174 10.1002/anie.201904174 30972886

[16] W. Lv, Z. Shen, X. Li, J. Meng, W. Yang et al., Discovering cathodic biocompatibility for aqueous Zn-MnO_2_ battery: an integrating biomass carbon strategy. Nano-Micro Lett. **16**(1), 109 (2024). 10.1007/s40820-024-01334-3 10.1007/s40820-024-01334-3 PMC10844190 38315253

[17] N. Zhang, Y. Dong, M. Jia, X. Bian, Y. Wang et al., Rechargeable aqueous Zn–V_2_O_5_ battery with high energy density and long cycle life. ACS Energy Lett. **3**(6), 1366–1372 (2018). 10.1021/acsenergylett.8b00565

[18] Z. Fang, C. Liu, X. Li, L. Peng, W. Ding et al., Systematic modification of MoO_3_-based cathode by the intercalation engineering for high-performance aqueous zinc-ion batteries. Adv. Funct. Mater. **33**(7), 2210010 (2023). 10.1002/adfm.202210010

[19] J. Liu, Z. Shen, C.-Z. Lu, Research progress of Prussian blue and its analogues for cathodes of aqueous zinc ion batteries. J. Mater. Chem. A **12**(5), 2647–2672 (2024). 10.1039/D3TA06641J

[20] R. Kumar, J. Shin, L. Yin, J.-M. You, Y.S. Meng et al., All-printed, stretchable Zn-Ag_2_O rechargeable battery *via* hyperelastic binder for self-powering wearable electronics. Adv. Energy Mater. **7**(8), 1602096 (2017). 10.1002/aenm.201602096

[21] R. Chen, P. Shi, Y. Gong, C. Yu, L. Hua et al., Solution-processable design of fiber-shaped wearable Zn// Ni(OH)_2_ battery. Energy Technol. **6**(12), 2326–2332 (2018). 10.1002/ente.201800318

[22] X.-W. Lv, Z. Wang, Z. Lai, Y. Liu, T. Ma et al., Rechargeable zinc–air batteries: advances, challenges, and prospects. Small **20**(4), 2306396 (2024). 10.1002/smll.202306396 10.1002/smll.202306396 37712176

[23] T. Najam, M. Wang, M.S. Javed, S. Ibraheem, Z. Song et al., Nano-engineering of Prussian blue analogues to core-shell architectures: enhanced catalytic activity for zinc-air battery. J. Colloid Interface Sci. **578**, 89–95 (2020). 10.1016/j.jcis.2020.05.071 32512399 10.1016/j.jcis.2020.05.071

[24] T. Najam, S.S. Ahmad Shah, W. Ding, J. Deng, Z. Wei, Enhancing by nano-engineering: hierarchical architectures as oxygen reduction/evolution reactions for zinc-air batteries. J. Power. Sources **438**, 226919 (2019). 10.1016/j.jpowsour.2019.226919

[25] M. Ulaganathan, Zinc–iron (Zn–Fe) redox flow battery single to stack cells: a futuristic solution for high energy storage off-grid applications. Energy Adv. **3**(12), 2861–2876 (2024). 10.1039/D4YA00358F

[26] N.S. Alghamdi, M. Rana, X. Peng, Y. Huang, J. Lee et al., Zinc-bromine rechargeable batteries: from device configuration, electrochemistry, material to performance evaluation. Nano-Micro Lett. **15**(1), 209 (2023). 10.1007/s40820-023-01174-7 10.1007/s40820-023-01174-7 PMC10471567 37650939

[27] J. Zhang, M. Shi, X. Ren, C. Wu, S. Hu et al., Low-cost, high-voltage and durable aqueous zinc-chlorine battery enabled by condensed choline chloride electrolytes. J. Energy Storage **88**, 111604 (2024). 10.1016/j.est.2024.111604

[28] W. Li, H. Xu, S. Ke, H. Zhang, H. Chen et al., Integrating electric ambipolar effect for high-performance zinc bromide batteries. Nano-Micro Lett. **17**(1), 143 (2025). 10.1007/s40820-024-01636-6 10.1007/s40820-024-01636-6 PMC11825431 39945958

[29] W. Shang, Q. Li, F. Jiang, B. Huang, J. Song et al., Boosting Zn||I(2) battery’s performance by coating a zeolite-based cation-exchange protecting layer. Nano-Micro Lett. **14**(1), 82 (2022). 10.1007/s40820-022-00825-5 10.1007/s40820-022-00825-5 PMC8956761 35334003

[30] Y. Zou, T. Liu, Q. Du, Y. Li, H. Yi et al., A four-electron Zn-I_2_ aqueous battery enabled by reversible I^-^/I_2_/I^+^ conversion. Nat. Commun. **12**(1), 170 (2021). 10.1038/s41467-020-20331-9 33419999 10.1038/s41467-020-20331-9 PMC7794333

[31] X. Kong, J. Zhang, X. Zhang, Z. Wang, D. Wang, Boosting reversible four-electron redox in aqueous Zn-iodine batteries with two halogen ionic additives and a N, F codoped carbon cathode. ACS Appl. Energy Mater. **8**(1), 601–610 (2025). 10.1021/acsaem.4c02898

[32] J. Guo, G. Ma, G. Liu, C. Dai, Z. Lin, Ti_2_CT_x_ MXene cathode host for enhanced zinc-bromine battery performance. Adv. Energy Mater. **14**(20), 2304516 (2024). 10.1002/aenm.202304516

[33] N. Chen, W. Wang, Y. Ma, M. Chuai, X. Zheng et al., Aqueous zinc-chlorine battery modulated by a MnO_2_ redox adsorbent. Small Methods **8**(6), e2201553 (2024). 10.1002/smtd.202201553 37086122 10.1002/smtd.202201553

[34] M. Rana, N. Alghamdi, X. Peng, Y. Huang, B. Wang et al., Scientific issues of zinc-bromine flow batteries and mitigation strategies. Exploration **3**(6), 20220073 (2023). 10.1002/EXP.20220073 38264684 10.1002/EXP.20220073 PMC10742200

[35] A. Mahmood, Z. Zheng, Y. Chen, Zinc-bromine batteries: challenges, prospective solutions, and future. Adv. Sci. **11**(3), 2305561 (2024). 10.1002/advs.202305561 10.1002/advs.202305561 PMC10797452 37988707

[36] Y. Zhang, L. Wang, Q. Li, B. Hu, J. Kang et al., Iodine promoted ultralow Zn nucleation overpotential and Zn-rich cathode for low-cost, fast-production and high-energy density anode-free Zn-iodine batteries. Nano-Micro Lett. **14**(1), 208 (2022). 10.1007/s40820-022-00948-9 10.1007/s40820-022-00948-9 PMC9606174 36289121

[37] D.-Q. Cai, H. Xu, T. Xue, J.-L. Yang, H.J. Fan, A synchronous strategy to Zn-iodine battery by polycationic long-chain molecules. Nano-Micro Lett. **18**(1), 3 (2025). 10.1007/s40820-025-01854-6 10.1007/s40820-025-01854-6 PMC12271045 40676288

[38] Y. Sui, M. Lei, M. Yu, A. Scida, S.K. Sandstrom et al., Reversible Cl_2_/Cl^–^ redox for low-temperature aqueous batteries. ACS Energy Lett. **8**(2), 988–994 (2023). 10.1021/acsenergylett.2c02757

[39] C. Wang, G. Gao, Y. Su, J. Xie, D. He et al., High-voltage and dendrite-free zinc-iodine flow battery. Nat. Commun. **15**(1), 6234 (2024). 10.1038/s41467-024-50543-2 39043688 10.1038/s41467-024-50543-2 PMC11266666

[40] H. Deng, X. Wang, Z. Wei, W. Liao, S. Li et al., Improved static membrane-free zinc-bromine batteries by an efficient bromine complexing agent. J. Energy Storage **81**, 110449 (2024). 10.1016/j.est.2024.110449

[41] L. Gao, Z. Li, Y. Zou, S. Yin, P. Peng et al., A high-performance aqueous zinc-bromine static battery. iScience **23**(8), 101348 (2020). 10.1016/j.isci.2020.101348 32711343 10.1016/j.isci.2020.101348 PMC7387827

[42] H. Yang, Y. Qiao, Z. Chang, H. Deng, P. He et al., A metal-organic framework as a multifunctional ionic sieve membrane for long-life aqueous zinc-iodide batteries. Adv. Mater. **32**(38), e2004240 (2020). 10.1002/adma.202004240 32797719 10.1002/adma.202004240

[43] Z. Yan, Q.-H. Yang, C. Yang, Elemental halogen cathodes for aqueous zinc batteries: mechanisms, challenges and strategies. J. Mater. Chem. A **12**(37), 24746–24760 (2024). 10.1039/D4TA05108D

[44] L. Yan, T. Liu, X. Zeng, L. Sun, X. Meng et al., Multifunctional porous carbon strategy assisting high-performance aqueous zinc-iodine battery. Carbon **187**, 145–152 (2022). 10.1016/j.carbon.2021.11.007

[45] Y. Li, X. Guo, S. Wang, W. Sun, D. Yu et al., Nano/micro metal-organic framework-derived porous carbon with rich nitrogen sites as efficient iodine hosts for aqueous zinc-iodine batteries. Adv. Sci. **12**(26), 2502563 (2025). 10.1002/advs.202502563 10.1002/advs.202502563 PMC12245103 40231444

[46] H. Xing, Y. Han, X. Huang, C. Zhang, M. Lyu et al., Recent progress of low-dimensional metal-organic frameworks for aqueous zinc-based batteries. Small **20**(36), e2402998 (2024). 10.1002/smll.202402998 38716678 10.1002/smll.202402998

[47] N. Sun, S.S. Ahmad Shah, Z. Lin, Y.-Z. Zheng, L. Jiao et al., MOF-based electrocatalysts: an overview from the perspective of structural design. Chem. Rev. **125**(5), 2703–2792 (2025). 10.1021/acs.chemrev.4c00664 40070208 10.1021/acs.chemrev.4c00664

[48] R. Naresh, K. Satchidhanandam, K.R. Ilancheran, B. Ambrose, M. Kathiresan et al., Bimetallic metal–organic framework: an efficient electrocatalyst for bromine-based flow batteries. J. Mater. Chem. A **12**(24), 14669–14678 (2024). 10.1039/d4ta02590c

[49] J. Li, Z. Xu, M. Wu, Reaction kinetics and mass transfer synergistically enhanced electrodes for high-performance zinc-bromine flow batteries. ACS Appl. Mater. Interfaces **17**(17), 25206–25215 (2025). 10.1021/acsami.4c22329 40248878 10.1021/acsami.4c22329 PMC12051167

[50] H. Wei, G. Qu, X. Zhang, B. Ren, S. Li et al., Boosting aqueous non-flow zinc–bromine batteries with a two-dimensional metal–organic framework host: an adsorption-catalysis approach. Energy Environ. Sci. **16**(9), 4073–4083 (2023). 10.1039/D3EE01639K

[51] Q. Liu, S. Wang, J. Lang, J. Wang, J. Zhan et al., Atomic synergy catalysis enables high-performing aqueous zinc-iodine batteries. Nano Lett. **25**(16), 6661–6669 (2025). 10.1021/acs.nanolett.5c00279 40227860 10.1021/acs.nanolett.5c00279

[52] J. Yang, Q. Dai, S. Hou, C. Han, L. Zhao, Anti-self-discharge capability of Zn-halogen batteries through an entrapment-adsorption-catalysis strategy built upon separator. Adv. Mater. **37**(11), 2418258 (2025). 10.1002/adma.202418258 10.1002/adma.202418258 39906923

[53] J. Liu, S. Chen, W. Shang, J. Ma, J. Zhang, *In situ* formation of 3D ZIF-8/MXene composite coating for high-performance zinc-iodine batteries. Adv. Funct. Mater. **35**(19), 2422081 (2025). 10.1002/adfm.202422081

[54] M. Han, D. Chen, Q. Lu, G. Fang, Aqueous rechargeable Zn-iodine batteries: issues, strategies and perspectives. Small **20**(18), e2310293 (2024). 10.1002/smll.202310293 38072631 10.1002/smll.202310293

[55] L. She, H. Cheng, Z. Yuan, Z. Shen, Q. Wu et al., Rechargeable aqueous zinc–halogen batteries: fundamental mechanisms, research issues, and future perspectives. Adv. Sci. **11**(8), 2305061 (2024). 10.1002/advs.202305061 10.1002/advs.202305061 PMC10953720 37939285

[56] D. Liu, Z. Wang, D. Zhao, S. Guo, L. Zhang et al., Design strategies and advanced methods for cathode engineering in aqueous zinc-iodine batteries. Small Methods **9**(9), e01287 (2025). 10.1002/smtd.202501287 40888395 10.1002/smtd.202501287

[57] X. Guo, H. Xu, Y. Tang, Z. Yang, F. Dou et al., Confining iodine into metal-organic framework derived metal-nitrogen-carbon for long-life aqueous zinc-iodine batteries. Adv. Mater. **36**(38), 2408317 (2024). 10.1002/adma.202408317 10.1002/adma.202408317 39081106

[58] B. Li, Z. Nie, M. Vijayakumar, G. Li, J. Liu et al., Ambipolar zinc-polyiodide electrolyte for a high-energy density aqueous redox flow battery. Nat. Commun. **6**, 6303 (2015). 10.1038/ncomms7303 25709083 10.1038/ncomms7303 PMC4346617

[59] G. Guo, Q. Dai, W. Li, S. Ke, H. Chen et al., Intercatenation weaves MOFs with conductive networks as iodine hosts for zinc-iodine batteries. Chem. Eng. J. **514**, 163100 (2025). 10.1016/j.cej.2025.163100

[60] Z. Li, X. Wu, X. Yu, S. Zhou, Y. Qiao et al., Long-life aqueous Zn–I_2_ battery enabled by a low-cost multifunctional zeolite membrane separator. Nano Lett. **22**(6), 2538–2546 (2022). 10.1021/acs.nanolett.2c00460 35266715 10.1021/acs.nanolett.2c00460

[61] D. Qi, H. Jiang, X. Chen, Y. Wang, H. Zhang et al., Design strategies, challenges, and prospects of nanomaterials for aqueous Zn–iodine batteries. ACS Nano (2025). 10.1021/acsnano.5c06585 10.1021/acsnano.5c06585 40590558

[62] Z. Wang, X. Meng, K. Chen, S. Mitra, High capacity aqueous periodate batteries featuring a nine-electron transfer process. Energy Storage Mater. **19**, 206–211 (2019). 10.1016/j.ensm.2019.02.021 31363505 10.1016/j.ensm.2019.02.021 PMC6666422

[63] F. Wang, J. Tseng, Z. Liu, P. Zhang, G. Wang et al., A stimulus-responsive zinc-iodine battery with smart overcharge self-protection function. Adv. Mater. **32**(16), e2000287 (2020). 10.1002/adma.202000287 32134521 10.1002/adma.202000287

[64] A. Khor, P. Leung, M.R. Mohamed, C. Flox, Q. Xu et al., Review of zinc-based hybrid flow batteries: from fundamentals to applications. Mater. Today Energy **8**, 80–108 (2018). 10.1016/j.mtener.2017.12.012

[65] X. Li, T. Li, P. Xu, C. Xie, Y. Zhang, A complexing agent to enable a wide-temperature range bromine-based flow battery for stationary energy storage. Adv. Funct. Mater. **31**(22), 2100133 (2021). 10.1002/adfm.202100133

[66] Y.-H. Lee, K. Shin, J. Baek, H.-T. Kim, Boosting the kinetics of bromine cathode in Zn–Br flow battery by enhancing the electrode adsorption of the droplet of bromine sequestration agent/polybromides complex. J. Power. Sources **620**, 235219 (2024). 10.1016/j.jpowsour.2024.235219

[67] Y. Wu, P.-W. Huang, J.D. Howe, Y. Yan, J. Martinez et al., In operando visualization of the electrochemical formation of liquid polybromide microdroplets. Angew. Chem. Int. Ed. **58**(43), 15228–15234 (2019). 10.1002/anie.201906980 10.1002/anie.201906980 31412156

[68] J. Li, Z. Xu, M. Wu, Halogen enabled aqueous flow cells for large-scale energy storage: current status and perspectives. J. Power. Sources **581**, 233477 (2023). 10.1016/j.jpowsour.2023.233477

[69] H. Chen, X. Li, K. Fang, H. Wang, J. Ning et al., Aqueous zinc-iodine batteries: from electrochemistry to energy storage mechanism. Adv. Energy Mater. **13**(41), 2302187 (2023). 10.1002/aenm.202302187

[70] S. Biswas, A. Senju, R. Mohr, T. Hodson, N. Karthikeyan et al., Minimal architecture zinc–bromine battery for low cost electrochemical energy storage. Energy Environ. Sci. **10**(1), 114–120 (2017). 10.1039/c6ee02782b

[71] Y. Huang, L. Lin, C. Zhang, L. Liu, Y. Li et al., Recent advances and strategies toward polysulfides shuttle inhibition for high‐performance Li–S batteries. Adv. Sci. **9**(12), 2106004 (2022). 10.1002/advs.202106004 10.1002/advs.202106004 PMC9036004 35233996

[72] D. Lin, Y. Li, Recent advances of aqueous rechargeable zinc-iodine batteries: challenges, solutions, and prospects. Adv. Mater. **34**(23), 2108856 (2022). 10.1002/adma.202108856 10.1002/adma.202108856 35119150

[73] Z. Bai, G. Wang, H. Liu, Y. Lou, N. Wang et al., Advancements in aqueous zinc–iodine batteries: a review. Chem. Sci. **15**(9), 3071–3092 (2024). 10.1039/d3sc06150g 38425533 10.1039/d3sc06150g PMC10901483

[74] J. Sun, Z. Wang, J. Zhang, D. Wang, Shuttle-free zinc–iodine batteries enabled by a cobalt single atom anchored on N-doped porous carbon host with ultra-high specific surface area. J. Energy Storage **90**, 111716 (2024). 10.1016/j.est.2024.111716

[75] Z. Xu, Q. Fan, Y. Li, J. Wang, P.D. Lund, Review of zinc dendrite formation in zinc bromine redox flow battery. Renew. Sustain. Energy Rev. **127**, 109838 (2020). 10.1016/j.rser.2020.109838

[76] P. Liang, J. Yi, X. Liu, K. Wu, Z. Wang et al., Highly reversible Zn anode enabled by controllable formation of nucleation sites for Zn‐based batteries. Adv. Funct. Mater. **30**(13), 1908528 (2020). 10.1002/adfm.201908528

[77] V. Yufit, F. Tariq, D.S. Eastwood, M. Biton, B. Wu et al., *Operando* visualization and multi-scale tomography studies of dendrite formation and dissolution in zinc batteries. Joule **3**(2), 485–502 (2019). 10.1016/j.joule.2018.11.002

[78] C. Xie, H. Zhang, W. Xu, W. Wang, X. Li, A long cycle life, self-healing zinc–iodine flow battery with high power density. Angew. Chem. Int. Ed. **57**(35), 11171–11176 (2018). 10.1002/anie.201803122 10.1002/anie.201803122 29717533

[79] W. Huang, L. Wang, Q. Zhu, P. Zhang, X. Pu et al., Alloying effects on inhibiting hydrogen evolution of Zn metal anode in rechargeable aqueous batteries. Mater. Today Commun. **33**, 104576 (2022). 10.1016/j.mtcomm.2022.104576

[80] L. Cao, D. Li, E. Hu, J. Xu, T. Deng et al., Solvation structure design for aqueous Zn metal batteries. J. Am. Chem. Soc. **142**(51), 21404–21409 (2020). 10.1021/jacs.0c09794 33290658 10.1021/jacs.0c09794

[81] Z. Yi, G. Chen, F. Hou, L. Wang, J. Liang, Strategies for the stabilization of Zn metal anodes for Zn-ion batteries. Adv. Energy Mater. **11**(1), 2003065 (2021). 10.1002/aenm.202003065

[82] W. Du, E.H. Ang, Y. Yang, Y. Zhang, M. Ye et al., Challenges in the material and structural design of zinc anode towards high-performance aqueous zinc-ion batteries. Energy Environ. Sci. **13**(10), 3330–3360 (2020). 10.1039/D0EE02079F

[83] Q. Li, Y. Zhao, F. Mo, D. Wang, Q. Yang et al., Dendrites issues and advances in Zn anode for aqueous rechargeable Zn-based batteries. EcoMat **2**(3), e12035 (2020). 10.1002/eom2.12035

[84] C. Li, X. Xie, S. Liang, J. Zhou, Issues and future perspective on zinc metal anode for rechargeable aqueous zinc-ion batteries. Energy Environ. Mater. **3**(2), 146–159 (2020). 10.1002/eem2.12067

[85] J. Wang, Y. Yang, Y. Zhang, Y. Li, R. Sun et al., Strategies towards the challenges of zinc metal anode in rechargeable aqueous zinc ion batteries. Energy Storage Mater. **35**, 19–46 (2021). 10.1016/j.ensm.2020.10.027

[86] W. Zhang, Y. Liu, X. Luo, R. Wang, K. Zhou et al., Multi-solvent synergy strategy unlocks anti-corrosion and high reversibility of zinc anodes: paving the way for robust and temperature-resilient zinc-iodine batteries. Adv. Funct. Mater. **35**(51), e12633 (2025). 10.1002/adfm.202512633

[87] H. Chen, L. Zhou, Y. Sun, T. Zhang, H. Wang et al., Bio-inspired biomass hydrogel interface with ion-selective responsive sieving mechanism for corrosion-resistant and dendrite-free zinc-iodine batteries. Energy Storage Mater. **76**, 104113 (2025). 10.1016/j.ensm.2025.104113

[88] D. Han, S. Shanmugam, Recent advances in the hybrid cathode for rechargeable zinc-bromine redox batteries. Curr. Opin. Electrochem. **45**, 101485 (2024). 10.1016/j.coelec.2024.101485

[89] X. Li, W. Xu, C. Zhi, Halogen-powered static conversion chemistry. Nat. Rev. Chem. **8**(5), 359–375 (2024). 10.1038/s41570-024-00597-z 38671189 10.1038/s41570-024-00597-z

[90] Z. Xue, Z. Gao, X. Zhao, Halogen storage electrode materials for rechargeable batteries. Energy Environ. Mater. **5**(4), 1155–1179 (2022). 10.1002/eem2.12442

[91] W. Gao, S. Cheng, Y. Zhang, E. Xie, J. Fu, Efficient charge storage in zinc–iodine batteries based on pre‐embedded iodine‐ions with reduced electrochemical reaction barrier and suppression of polyiodide self‐shuttle effect. Adv. Funct. Mater. **33**(17), 2211979 (2023). 10.1002/adfm.202211979

[92] Q. Jin, K. Zhao, L. Wu, L. Li, L. Kong et al., Enhancing Li cycling coulombic efficiency while mitigating “shuttle effect” of Li-S battery through sustained release of LiNO_3_. J. Energy Chem. **84**, 22–29 (2023). 10.1016/j.jechem.2023.05.020

[93] S. Li, Y. Nie, Y. Wang, G. Feng, Q. Li et al., Quantum size effect synergizes space-limited domain action for advanced aqueous zinc-iodine batteries. Adv. Mater. (2025). 10.1002/adma.202514577 10.1002/adma.202514577 PMC12810656 41185985

[94] Q. Zhang, S. Jiang, T. Lv, Y. Peng, H. Pang, Application of conductive MOF in zinc‐based batteries. Adv. Mater. **35**(48), 2305532 (2023). 10.1002/adma.202305532 10.1002/adma.202305532 37382197

[95] L. Zhang, Y. Hou, The rise and development of MOF‐based materials for metal‐chalcogen batteries: current status, challenges, and prospects. Adv. Energy Mater. **13**(20), 2204378 (2023). 10.1002/aenm.202204378

[96] N. Li, Z. Yang, Y. Li, D. Yu, T. Pan et al., Size confinement strategy effect enables advanced aqueous zinc–iodine batteries. Adv. Energy Mater. **14**(44), 2402846 (2024). 10.1002/aenm.202402846

[97] J. Xu, J. Wang, L. Ge, J. Sun, W. Ma et al., ZIF-8 derived porous carbon to mitigate shuttle effect for high performance aqueous zinc-iodine batteries. J. Colloid Interface Sci. **610**, 98–105 (2022). 10.1016/j.jcis.2021.12.043 34922086 10.1016/j.jcis.2021.12.043

[98] J. Ye, W. Tian, Y. Du, J. Ji, Defect-engineered ZIF-derived carbon hosts for long-life aqueous zinc-iodine batteries. Adv. Funct. Mater. **35**(47), 2509582 (2025). 10.1002/adfm.202509582

[99] Y. He, M. Liu, S. Chen, J. Zhang, Shapeable carbon fiber networks with hierarchical porous structure for high-performance Zn-I_2_ batteries. Sci. China Chem. **65**(2), 391–398 (2022). 10.1007/s11426-021-1177-1

[100] C. Wang, Q. Lai, K. Feng, P. Xu, X. Li et al., From zeolite-type metal organic framework to porous nano-sheet carbon: high activity positive electrode material for bromine-based flow batteries. Nano Energy **44**, 240–247 (2018). 10.1016/j.nanoen.2017.12.007

[101] Y. Wang, X. Zhang, X. Li, Y. Jiang, T. Shen et al., Entrapping polyiodide by using highly N, P co-doping porous carbon framework towards high performance zinc-iodine batteries. Diamond Relat. Mater. **150**, 111685 (2024). 10.1016/j.diamond.2024.111685

[102] J. Sun, H. Ma, D. Wang, Heavily heteroatoms doped carbons with tunable microstructure as the iodine hosts for rechargeable zinc-iodine aqueous batteries. J. Alloys Compd. **947**, 169696 (2023). 10.1016/j.jallcom.2023.169696

[103] S. Chai, J. Yao, Y. Wang, J. Zhu, J. Jiang, Mediating iodine cathodes with robust directional halogen bond interactions for highly stable rechargeable Zn-I_2_ batteries. Chem. Eng. J. **439**, 135676 (2022). 10.1016/j.cej.2022.135676

[104] S. Niu, B. Zhao, D. Liu, High-performance Zn–I_2_ batteries enabled by a metal-free defect-rich carbon cathode catalyst. ACS Appl. Mater. Interfaces **15**(21), 25558–25566 (2023). 10.1021/acsami.3c03134 37198728 10.1021/acsami.3c03134

[105] X. Yang, H. Fan, F. Hu, S. Chen, K. Yan et al., Aqueous zinc batteries with ultra-fast redox kinetics and high iodine utilization enabled by iron single atom catalysts. Nano-Micro Lett. **15**(1), 126 (2023). 10.1007/s40820-023-01093-7 10.1007/s40820-023-01093-7 PMC10199998 37209237

[106] T. Liu, H. Wang, C. Lei, Y. Mao, H. Wang et al., Recognition of the catalytic activities of graphitic N for zinc-iodine batteries. Energy Storage Mater. **53**, 544–551 (2022). 10.1016/j.ensm.2022.09.028

[107] J. Yang, Y. Kang, F. Meng, W. Meng, G. Chen et al., Theoretical calculation-driven rational screening of d-block single-atom electrocatalysts based on d–p orbital hybridization for durable aqueous zinc–iodine batteries. Energy Environ. Sci. **18**(1), 236–245 (2025). 10.1039/D4EE04119D

[108] S.S. Ahmad Shah, T. Najam, M.S. Javed, M.S. Bashir, M.A. Nazir et al., Recent advances in synthesis and applications of single-atom catalysts for rechargeable batteries. Chem. Rec. **22**(7), e202100280 (2022). 10.1002/tcr.202100280 34921492 10.1002/tcr.202100280

[109] H. Li, X. Kang, M. Zhu, Nanocluster-based aggregates: assembled forms, driving forces, and structure-related properties. Coord. Chem. Rev. **539**, 216738 (2025). 10.1016/j.ccr.2025.216738

[110] L. Chai, X. Wang, Y. Hu, X. Li, S. Huang et al., In-MOF-derived hierarchically hollow carbon nanostraws for advanced zinc-iodine batteries. Adv. Sci. **9**(33), 2105063 (2022). 10.1002/advs.202105063 10.1002/advs.202105063 PMC9685461 36181364

[111] X. Wang, Q. Zhang, S. Chu, T. Qin, Q. Liu et al., Fe/N co-doped micro-mesoporous carbon nanofibers as high-performance catalysts for zinc-bromine flow batteries. Prog. Natural Sci. Mater. Int. **35**(5), 955–962 (2025). 10.1016/j.pnsc.2025.07.008

[112] B. Li, J. Liu, Z. Nie, W. Wang, D. Reed et al., Metal–organic frameworks as highly active electrocatalysts for high-energy density, aqueous zinc-polyiodide redox flow batteries. Nano Lett. **16**(7), 4335–4340 (2016). 10.1021/acs.nanolett.6b01426 27267589 10.1021/acs.nanolett.6b01426

[113] H.K. Machhi, K.K. Sonigara, S.N. Bariya, H.P. Soni, S.S. Soni, Hierarchically porous metal–organic gel hosting catholyte for limiting iodine diffusion and self-discharge control in sustainable aqueous zinc–I_2_ batteries. ACS Appl. Mater. Interfaces **13**(18), 21426–21435 (2021). 10.1021/acsami.1c03812 33938731 10.1021/acsami.1c03812

[114] J. He, Y. Mu, B. Wu, F. Wu, R. Liao et al., Synergistic effects of Lewis acid–base and Coulombic interactions for high-performance Zn–I_2_ batteries. Energy Environ. Sci. **17**(1), 323–331 (2024). 10.1039/D3EE03297C

[115] X. Pan, K. Song, Y. Zhu, M. Yang, M. Ren et al., Functional nanoarchitectonics with metal-organic framework derived porous carbon as an efficient iodine host for high performance aqueous zinc-iodine batteries. J. Electroanal. Chem. **1000**, 119645 (2026). 10.1016/j.jelechem.2025.119645

[116] Y. Hou, F. Kong, Z. Wang, M. Ren, C. Qiao et al., High performance rechargeable aqueous zinc-iodine batteries *via* a double iodine species fixation strategy with mesoporous carbon and modified separator. J. Colloid Interface Sci. **629**, 279–287 (2023). 10.1016/j.jcis.2022.09.079 36155923 10.1016/j.jcis.2022.09.079

[117] S. Shin, D. Jung, J. Chae, J. Chang, Stochastic electrochemical analysis of electrochemically generated ethylpyridinium polybromide droplets: evidence of Br^−^/Br_3_^−^/Br_2_ electro-oxidation in quaternary ammonium polybromide. J. Electroanal. Chem. **802**, 123–130 (2017). 10.1016/j.jelechem.2017.08.021

[118] W. Han, J. Zhao, X. Li, Long cycle life Zn-I_2_ batteries: utilizing Co/N Co-doped carbon matrix derived from zeolitic imidazolate framework-67 as a bifunctional iodine host. J. Alloys Compd. **1008**, 176647 (2024). 10.1016/j.jallcom.2024.176647

[119] S. Chen, Y. He, S. Ding, J. Zhang, *In situ* formation of tungsten nitride among porous carbon polyhedra for high performance zinc–iodine batteries. J. Phys. Chem. C **127**(16), 7609–7617 (2023). 10.1021/acs.jpcc.3c00678

[120] Y. He, M. Liu, J. Zhang, Rational modulation of carbon fibers for high-performance zinc–iodine batteries. Adv. Sustain. Syst. **4**(11), 2000138 (2020). 10.1002/adsu.202000138

[121] S.S. Ahmad Shah, T. Najam, M.S. Bashir, L. Peng, M.A. Nazir et al., Single-atom catalysts for next-generation rechargeable batteries and fuel cells. Energy Storage Mater. **45**, 301–322 (2022). 10.1016/j.ensm.2021.11.049

[122] S. Chen, J. Ma, Q. Chen, W. Shang, J. Liu et al., Exploring interfacial electrocatalysis for iodine redox conversion in zinc-iodine battery. Sci. Bull. **70**(4), 546–555 (2025). 10.1016/j.scib.2024.11.042 10.1016/j.scib.2024.11.042 39694795

[123] J. Lee, W. Lee, S. Back, S.Y. Yi, S. Lee et al., Activating iodine redox by enabling single-atom coordination to dormant nitrogen sites to realize durable zinc–iodine batteries. EES Catal. **2**(1), 276–285 (2024). 10.1039/D3EY00228D

[124] X. Guo, H. Xu, Z. Qiu, Q. Li, N. Li et al., Heteroatom-modulated asymmetric cobalt single-atom catalysts on MOF-derived carbon enabling durable zinc-iodine batteries. Adv. Mater. **37**(45), e14035 (2025). 10.1002/adma.202514035 40847723 10.1002/adma.202514035

[125] T. Hu, Y. Zhao, Y. Yang, H. Lv, R. Zhong et al., Development of inverse-opal-structured charge-deficient Co_9_S_8_@nitrogen-doped-carbon to catalytically enable high energy and high power for the two-electron transfer I^+^/I^−^ electrode. Adv. Mater. **36**(18), 2312246 (2024). 10.1002/adma.202312246 10.1002/adma.202312246 38266255

[126] W. Du, L. Miao, Z. Song, X. Zheng, C. Hu et al., Organic iodine electrolyte starting triple I^+^ storage in in-based metal-organic frameworks for high-capacity aqueous Zn-I_2_ batteries. Chem. Eng. J. **484**, 149535 (2024). 10.1016/j.cej.2024.149535

[127] S. Shoaib Ahmad Shah, M. Altaf Nazir, A. Mahmood, M. Sohail, A. Ur Rehman et al., Synthesis of electrical conductive metal-organic frameworks for electrochemical applications. Chem. Rec. **24**(1), e202300141 (2024). 10.1002/tcr.202300141 37724006 10.1002/tcr.202300141

[128] J.-H. Lee, Y. Byun, G.H. Jeong, C. Choi, J. Kwen et al., High-energy efficiency membraneless flowless Zn–Br battery: utilizing the electrochemical–chemical growth of polybromides. Adv. Mater. **31**(52), 1904524 (2019). 10.1002/adma.201904524 10.1002/adma.201904524 31650656

[129] Z. Wang, J. Huang, Z. Guo, X. Dong, Y. Liu et al., A metal-organic framework host for highly reversible dendrite-free zinc metal anodes. Joule **3**(5), 1289–1300 (2019). 10.1016/j.joule.2019.02.012

[130] J.-H. Lee, R. Kim, S. Kim, J. Heo, H. Kwon et al., Dendrite-free Zn electrodeposition triggered by interatomic orbital hybridization of Zn and single vacancy carbon defects for aqueous Zn-based flow batteries. Energy Environ. Sci. **13**(9), 2839–2848 (2020). 10.1039/D0EE00723D

[131] J. Wu, Q. Dai, H. Zhang, X. Li, A defect-free MOF composite membrane prepared *via in situ* binder-controlled restrained second-growth method for energy storage device. Energy Storage Mater. **35**, 687–694 (2021). 10.1016/j.ensm.2020.11.040

[132] P. Yang, K. Zhang, S. Liu, W. Zhuang, Z. Shao et al., Ionic selective separator design enables long-life zinc–iodine batteries *via* synergistic anode stabilization and polyiodide shuttle suppression. Adv. Funct. Mater. **34**(52), 2410712 (2024). 10.1002/adfm.202410712

[133] D. Han, K. Shin, H.-T. Kim, S. Shanmugam, Functionalized metal–organic framework modified membranes with ultralong cyclability and superior capacity for zinc/bromine flowless batteries. J. Mater. Chem. A **12**(23), 13970–13979 (2024). 10.1039/D4TA01005A

[134] L. Zhu, X. Guan, Z. Zhang, Z. Yuan, C. Zhang et al., Polar-nonpolar synergy toward high-performance aqueous zinc-iodine batteries. Small **21**(13), 2500223 (2025). 10.1002/smll.202500223 39981799 10.1002/smll.202500223 PMC11962694

[135] L. Wang, J. Guan, N. Li, J. Li, T. Duan et al., Amine-functionalized MIL-125 separator and MOF-derived carbon host for high-performance aqueous zinc-iodine batteries. Adv. Energy Mater. **15**(45), e04201 (2025). 10.1002/aenm.202504201

[136] W. He, S. Zuo, X. Xu, L. Zeng, L. Liu et al., Challenges and strategies of zinc anode for aqueous zinc-ion batteries. Mater. Chem. Front. **5**(5), 2201–2217 (2021). 10.1039/D0QM00693A

[137] S. So, Y.N. Ahn, J. Ko, I.T. Kim, J. Hur, Uniform and oriented zinc deposition induced by artificial Nb_2_O_5_ layer for highly reversible Zn anode in aqueous zinc ion batteries. Energy Storage Mater. **52**, 40–51 (2022). 10.1016/j.ensm.2022.07.036

[138] H. Liu, J.-G. Wang, W. Hua, L. Ren, H. Sun et al., Navigating fast and uniform zinc deposition via a versatile metal–organic complex interphase. Energy Environ. Sci. **15**(5), 1872–1881 (2022). 10.1039/D2EE00209D

[139] H. Wang, H. Li, Y. Tang, Z. Xu, K. Wang et al., Stabilizing Zn anode interface by simultaneously manipulating the thermodynamics of Zn nucleation and overpotential of hydrogen evolution. Adv. Funct. Mater. **32**(48), 2207898 (2022). 10.1002/adfm.202207898

[140] A. Chen, C. Zhao, J. Gao, Z. Guo, X. Lu et al., Multifunctional SEI-like structure coating stabilizing Zn anodes at a large current and capacity. Energy Environ. Sci. **16**(1), 275–284 (2023). 10.1039/D2EE02931F

[141] Z. Guo, L. Fan, C. Zhao, A. Chen, N. Liu et al., A dynamic and self‐adapting interface coating for stable Zn‐metal anodes. Adv. Mater. **34**(2), 2105133 (2022). 10.1002/adma.202105133 10.1002/adma.202105133 34676914

[142] W. Zhang, W. Qi, K. Yang, Y. Hu, F. Jiang et al., Boosting tough metal Zn anode by MOF layer for high-performance zinc-ion batteries. Energy Storage Mater. **71**, 103616 (2024). 10.1016/j.ensm.2024.103616

[143] Q. Cao, H. Gao, Y. Gao, J. Yang, C. Li et al., Regulating dendrite-free zinc deposition by 3D zincopilic nitrogen-doped vertical graphene for high-performance flexible Zn-ion batteries. Adv. Funct. Mater. **31**(37), 2103922 (2021). 10.1002/adfm.202103922

[144] Q. Nian, X. Yang, H. Hong, P. Chen, Y. Zhao et al., Advancements in separator materials for aqueous zinc batteries. Nanoscale Horiz. **10**(9), 1932–1955 (2025). 10.1039/D5NH00172B 40620137 10.1039/d5nh00172b

[145] C.-Y. Liu, Y.-D. Wang, H. Liu, Q. Chen, X. Jiang et al., Channel engineering strategy of precisely modified MOF/nanofiber composite separator for advanced aqueous zinc ion batteries. Compos. Part B Eng. **272**, 111227 (2024). 10.1016/j.compositesb.2024.111227

[146] T. Yuan, S. Qi, L. Ye, Y. Zhao, Y. Jiang et al., Metal-organic frameworks-based materials: a feasible path for redox flow battery. Coord. Chem. Rev. **531**, 216503 (2025). 10.1016/j.ccr.2025.216503

[147] H. Di, Y. An, J. Yang, D. Luan, X.W. Lou, Fluorine modified zeolitic imidazolate framework enables long-life Zn–I_2_ batteries by suppression of polyiodide shuttle. Angew. Chem. Int. Ed. **64**(43), e202513312 (2025). 10.1002/anie.202513312 10.1002/anie.202513312 PMC12535394 40899615

[148] H. Zheng, J. Ding, J. Huang, J. Wang, H. Zhang et al., Metal-organic frameworks facilitating complexation for long-cycle zinc-bromine flow batteries. Adv. Funct. Mater. (2025). 10.1002/adfm.202514730

[149] X.-D. Zhou, X.-X. Wu, H.-J. Fu, P.-X. Lei, N. Li et al., Self-assembled Co-MOF@MXene heterostructures as bifunctional electrocatalysts for high-performance lithium-sulfur batteries. Mater. Today Energy **52**, 101950 (2025). 10.1016/j.mtener.2025.101950

